# Circulating and Tissue-Resident CD4^+^ T Cells With Reactivity to Intestinal Microbiota Are Abundant in Healthy Individuals and Function Is Altered During Inflammation

**DOI:** 10.1053/j.gastro.2017.07.047

**Published:** 2017-11

**Authors:** Ahmed N. Hegazy, Nathaniel R. West, Michael J.T. Stubbington, Emily Wendt, Kim I.M. Suijker, Angeliki Datsi, Sebastien This, Camille Danne, Suzanne Campion, Sylvia H. Duncan, Benjamin M.J. Owens, Holm H. Uhlig, Andrew McMichael, Oxford IBD Cohort Investigators, Andreas Bergthaler, Sarah A. Teichmann, Satish Keshav, Fiona Powrie

**Affiliations:** 1 Translational Gastroenterology Unit, Nuffield Department of Clinical Medicine, Experimental Medicine Division, John Radcliffe Hospital, University of Oxford, United Kingdom; 2 Kennedy Institute of Rheumatology, Nuffield Department of Orthopaedics, Rheumatology and Musculoskeletal Sciences, University of Oxford, United Kingdom; 3 European Molecular Biology Laboratory-European Bioinformatics Institute, Hinxton, United Kingdom; 4 Wellcome Trust Sanger Institute, Wellcome Trust Genome Campus, Hinxton, Cambridge, United Kingdom; 5 Nuffield Department of Medicine Research Building, University of Oxford, Oxford, United Kingdom; 6 Microbial Ecology Group, Rowett Institute of Nutrition and Health, University of Aberdeen, Aberdeen, United Kingdom; 7 Department of Paediatrics, University of Oxford, Oxford, United Kingdom; 8 CeMM Research Center for Molecular Medicine of the Austrian Academy of Sciences, Vienna, Austria

**Keywords:** Immune Regulation, Microbiota, Cytokines, Tissue-resident Memory T cells, CFSE, carboxy-fluorescein succinimidyl ester, IBD, inflammatory bowel disease, ICOS, inducible T-cell costimulator, IFN, interferon, IL, interleukin, LPMC, Lamina propria mononuclear cell, PBMC, peripheral blood mononuclear cell, SEB, *Staphylococcus* enterotoxin B, TCR, T-cell receptor, Th, T helper, TNF, tumor necrosis factor

## Abstract

**Background & Aims:**

Interactions between commensal microbes and the immune system are tightly regulated and maintain intestinal homeostasis, but little is known about these interactions in humans. We investigated responses of human CD4^+^ T cells to the intestinal microbiota. We measured the abundance of T cells in circulation and intestinal tissues that respond to intestinal microbes and determined their clonal diversity. We also assessed their functional phenotypes and effects on intestinal resident cell populations, and studied alterations in microbe-reactive T cells in patients with chronic intestinal inflammation.

**Methods:**

We collected samples of peripheral blood mononuclear cells and intestinal tissues from healthy individuals (controls, n = 13−30) and patients with inflammatory bowel diseases (n = 119; 59 with ulcerative colitis and 60 with Crohn’s disease). We used 2 independent assays (CD154 detection and carboxy-fluorescein succinimidyl ester dilution assays) and 9 intestinal bacterial species (*Escherichia coli, Lactobacillus acidophilus, Bifidobacterium animalis* subsp *lactis, Faecalibacterium prausnitzii, Bacteroides vulgatus, Roseburia intestinalis, Ruminococcus obeum, Salmonella typhimurium,* and *Clostridium difficile*) to quantify, expand, and characterize microbe-reactive CD4^+^ T cells. We sequenced T-cell receptor Vβ genes in expanded microbe-reactive T-cell lines to determine their clonal diversity. We examined the effects of microbe-reactive CD4^+^ T cells on intestinal stromal and epithelial cell lines. Cytokines, chemokines, and gene expression patterns were measured by flow cytometry and quantitative polymerase chain reaction.

**Results:**

Circulating and gut-resident CD4^+^ T cells from controls responded to bacteria at frequencies of 40−4000 per million for each bacterial species tested. Microbiota-reactive CD4^+^ T cells were mainly of a memory phenotype, present in peripheral blood mononuclear cells and intestinal tissue, and had a diverse T-cell receptor Vβ repertoire. These cells were functionally heterogeneous, produced barrier-protective cytokines, and stimulated intestinal stromal and epithelial cells via interleukin 17A, interferon gamma, and tumor necrosis factor. In patients with inflammatory bowel diseases, microbiota-reactive CD4^+^ T cells were reduced in the blood compared with intestine; T-cell responses that we detected had an increased frequency of interleukin 17A production compared with responses of T cells from blood or intestinal tissues of controls.

**Conclusions:**

In an analysis of peripheral blood mononuclear cells and intestinal tissues from patients with inflammatory bowel diseases vs controls, we found that reactivity to intestinal bacteria is a normal property of the human CD4^+^ T-cell repertoire, and does not necessarily indicate disrupted interactions between immune cells and the commensal microbiota. T-cell responses to commensals might support intestinal homeostasis, by producing barrier-protective cytokines and providing a large pool of T cells that react to pathogens.

See Covering the Cover synopsis on page 1175.

**Editor's Notes**

**Background and Context**

CD4^+^ T cell responses to intestinal bacteria are known to occur, however these responses remain poorly characterized in humans.

**New Findings**

Microbiota-reactive CD4^+^ T cells are prevalent and normal constituents of the human immune system that are functionally altered during IBD pathogenesis.

**Limitations**

The functional relevance of the detected T-cell responses in humans remains to be elucidated.

**Impact**

T-cell responses to commensals might support intestinal homeostasis by producing barrier-protective cytokines and providing a large pool of T cells with potential cross-reactivity to pathogens.

Vast numbers of microbes populate the gastrointestinal tract and contribute to digestion, epithelial barrier integrity, and development of appropriately educated mucosal immunity.^1^ Intestinal immune responses are tightly regulated to allow protective immunity against pathogens, while limiting responses to dietary antigens and innocuous microbes. The “mucosal firewall” prevents systemic dissemination of microbes by confining microbial antigens to the gut-associated lymphoid tissue.^2^ In the gut-associated lymphoid tissue, dendritic cells drive regulatory T-cell differentiation in response to dietary antigens and commensal bacteria.^3^ Nevertheless, vast numbers of potentially commensal-reactive effector and memory T cells populate intestinal mucosae.^4^ Recent evidence suggests that in mice, tolerance to commensal-derived antigens may be lost during pathogen-induced epithelial damage and subsequent transient exposure to commensals.^1^, ^5^ In humans, circulating memory T cells recognize peptides derived from gut bacteria and can cross-react to pathogens, which can confer immunologic advantage during subsequent new infections.^6^, ^7^ Although this process can be beneficial during homeostasis, deranged responses to commensals may promote inflammatory conditions, such as inflammatory bowel diseases (IBDs).

IBDs (including Crohn’s disease and ulcerative colitis) result from a prolonged disturbance of gut homeostasis, the precise etiology of which is uncertain. One hypothesis is that, in genetically susceptible individuals, IBD may be triggered by intestinal dysbiosis that promotes aberrant immune stimulation.^8^ Indeed, in mouse models of colitis, intestinal microbiota promote inflammation in part by stimulating microbiota-reactive CD4^+^ T cells.^5^, ^9^ Whether this drives IBD in humans, however, remains unknown.

Although CD4^+^ T-cell responses to intestinal bacteria are known to occur in humans,^10^, ^11^, ^12^ several aspects of this topic are largely uncharacterized, including the frequency of human T cells in the gut and periphery that are reactive to phylogenetically distinct intestinal microbes; the T-cell receptor (TCR) diversity and clonotype sharing of these T cells; the functional phenotype of gut microbe-reactive T cells and their impact on tissue-resident cell populations; and how microbe-reactive T cells change during chronic intestinal inflammation. To address this knowledge gap, we extensively characterized CD4^+^ T-cell responses to intestinal microbiota in healthy individuals and IBD patients.

Using 2 independent assays, we observed that for almost all enteric bacteria examined, bacteria-reactive CD4^+^ T cells were present at a frequency of 40−500 per million CD4^+^ T cells in adult peripheral blood. Bacteria-reactive T cells were also prevalent in the gut mucosa, with prominent enrichment for proteobacteria reactivity. Microbiota-responsive T cells showed a diverse TCR Vβ repertoire and potently stimulated inflammatory responses by intestinal epithelial and stromal cells. Intriguingly, T cells from IBD patients displayed a normal spectrum of microbial responses, but expressed high amounts of interleukin (IL) 17A, consistent with increased amounts of T-helper (Th) 17-polarizing cytokines in inflamed intestinal tissue. Collectively, these data demonstrate that microbiota-reactive CD4^+^ T cells are prevalent and normal constituents of the human immune system that are functionally altered during IBD pathogenesis.

## Materials and Methods

### Human Samples and Cell Isolation

Leukoreduction chambers from healthy individuals were obtained from the National Blood Service (Bristol, UK). Peripheral EDTA blood samples were obtained from IBD patients attending the John Radcliffe Hospital Gastroenterology unit or from healthy in-house volunteers. IBD patients (n = 119; ulcerative colitis, n = 59; Crohn’s disease, n = 60) diagnosed by endoscopic, histologic, and radiologic criteria were recruited for the study. Healthy volunteers (n = 30) without any known underlying acute or chronic pathologic condition served as control donors. All donors provided informed written consent. The National Health Service Research Ethics System provided ethical approval (reference numbers 09/H0606/5 for IBD patients and 11/YH/0020 for controls; OCHRe ref 15/A237 for cord blood samples). For details regarding cell isolation, see Supplementary Experimental Procedures.

### CD154-Based Detection of Antigen-Specific T Cells

CD154 detection was done as described previously.^13^, ^14^ Briefly, cells were plated at 5 × 10^6^/cm^2^ for 7−12 hours with heat-inactivated bacteria. Brefeldin A (5 μg/mL; Sigma Aldrich, St Louis, MO) was added at 2 hours. After 8−12 hours, cells were harvested and treated as described in the section on intracellular cytokine, CD154, and transcription factor staining. For magnetic cell separation enrichment of CD4^+^CD154^+^ T cells, see Supplementary Experimental Procedures.

### Antigen-Specific Recall Response (Carboxy-Fluorescein Succinimidyl Ester Dilution Assay) and T-Cell Culture

Memory CD4^+^ CD45RO^+^ CD45RA^−^ T cells were enriched from peripheral blood mononuclear cells (PBMCs) with untouched memory CD4^+^ T cell enrichment kit (Miltenyi Biotec, Bergisch Gladbach, Germany), sorted to >97% purity on a FACS ARIA III (BD, San Jose, CA) using CD45RA and CD45RO expression, and were labeled with carboxy-fluorescein succinimidyl ester (CFSE) or violet proliferation dye (Invitrogen, Carlsbad, CA). CD14^+^ monocytes were isolated from PBMC using anti-CD14 microbeads (Miltenyi Biotec), irradiated (45 Gy) and then pre-incubated for 3 hours with bacterial lysates before T-cell co-culture. T cells were co-cultured with the irradiated autologous monocytes at a ratio of 2:1 for 5−7 days. Cells were cultured in RPMI-1640 supplemented with 2 mM glutamine, 1% (v/v) non-essential amino acids, 1% (v/v) sodium pyruvate, penicillin (50 U/mL), streptomycin (50 mg/mL; all from Invitrogen), and 5% (v/v) human serum (National Blood Service, Bristol, UK). CD14^+^ monocytes were irradiated (45 Gy) and then pre-incubated for 3 hours with bacterial lysates before T-cell co-culture. For major histocompatibility complex II blockade, 10 μg/mL of a pan-HLA class-II blocking antibody (HLA-DR, DP, DQ [Tü39]) was added 30 minutes before T-cell co-culture. T-cell lines were generated by sorting CFSE^low^ inducible T-cell costimulator (ICOS)^high^ CD4^+^ T cells after 7 days of stimulation and expanding them with IL2 (300 U/mL) and anti-CD3/CD28 beads (beads/T cell ratio, 1:4, Dynals) for 10−14 days. Supernatants were collected from 1 × 10^6^ CD4^+^ T cells stimulated for 24 hours with phorbol myristate acetate (5 ng/mL) and ionomycin (500 ng/mL; Sigma).

### Flow Cytometry and Cell Sorting

PBMCs and lamina propria mononuclear cells were stained according to standard protocols. For details, see Supplementary Experimental Procedures.

### Intracellular Cytokine, CD154, and Transcription Factor Staining

For intracellular cytokine staining, cells were stained with fixable viability dye eFluor 780 (eBioscience, San Diego, CA) and surface markers, fixed with 2% formaldehyde (Merck, Kenilworth, NJ), and stained for cytokines in magnetic cell separation buffer containing 0.05% saponin (Sigma-Aldrich). Transcription factor expression was analyzed using the FoxP3 staining buffer set (eBioscience) according to manufacturer’s instructions.

### Stimulation of Intestinal Cell Lines

CCD18Co (non-transformed human colon fibroblasts; ATCC, Manassas, VA), and LIM1863 (human colon epithelial cells; a kind gift of Dr Robert Whitehead, Ludwig Institute for Cancer Research) cells were cultured in humidified incubators with 5% CO_2_ at 37°C in Dulbecco’s modified Eagle medium or RPMI media (Sigma) with 10% fetal calf serum (Sigma) and 100 U penicillin/0.1 mg/mL streptomycin. Cells were stimulated with 5% T-cell supernatants for 24 hours. Cytokine neutralization was achieved by supernatant pre-incubation for 1−2 hours with 10 μg/mL anti−IL17A (eBio64CAP17), anti−interferon (IFN) gamma (B27), anti−tumor necrosis factor (TNF)−α (Remicade; Janssen Biotech, Horsham, PA), and anti−IL22 (IL22JOP).

### Statistics

Statistical analyses were performed with GraphPad Prism, version 6.0 for Macintosh (GraphPad Software, La Jolla, CA). Statistically significant *P* values were indicated as follows: NS, not significant; ^∗^*P* ≤ .05; ^∗∗^*P* ≤ .01; ^∗∗∗^*P* ≤ .001; ^∗∗∗∗^*P* ≤ .0001. Tests are specified in figure legends.

## Results

### Healthy Adults Possess Circulating Memory CD4^+^ T Cells That Are Reactive to Intestinal Microbiota

**Figure 1:**
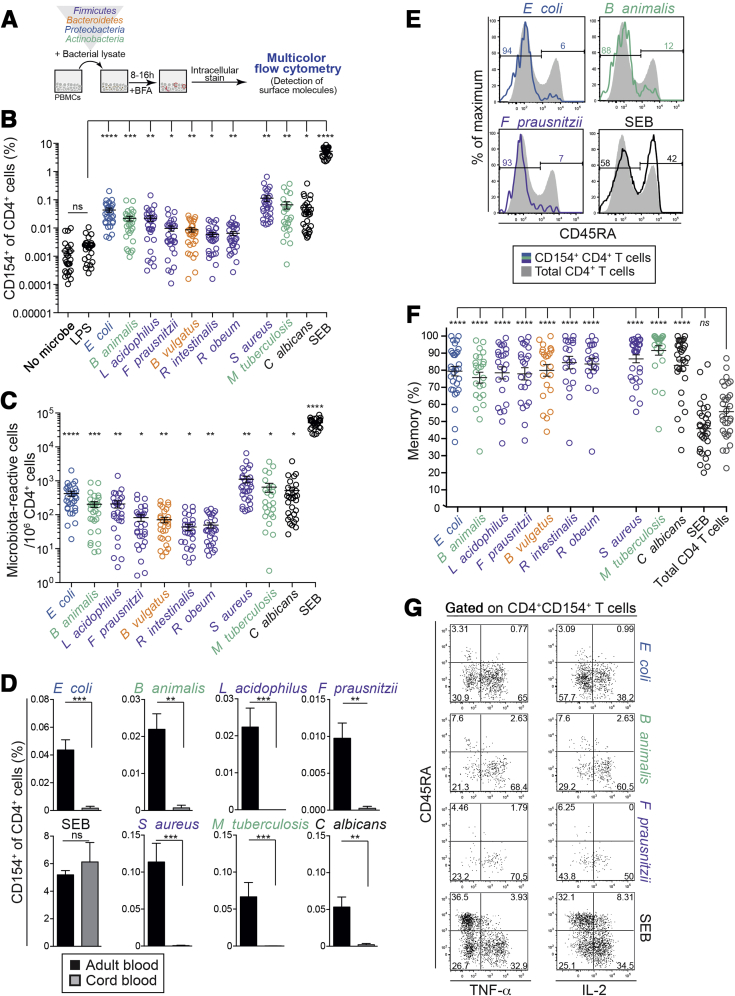
Healthy adults possess circulating memory CD4^+^ T cells that are reactive to intestinal microbiota. PBMCs were stimulated with heat-inactivated bacteria for 8−12 hours, and bacteria-reactive CD4^+^ T cells were detected by intracellular CD154 expression. (*A*) Experimental setup. (*B*) CD154^+^ frequencies among peripheral CD4^+^ T cells in adults after short-term stimulation with heat-inactivated bacteria (n = 30 independent donors). (*C*) Estimated microbiota-reactive cells per million CD4^+^ T cells in adult blood (n = 30). The numbers of microbiota-reactive T cells were calculated based on the frequencies of CD4^+^CD154^+^ T cells. Background (no microbe stimulation) was subtracted from bacterial stimulations. Significance calculated in relation to lipopolysaccharide stimulation. (*D*) Frequencies (±SEM) of CD154^+^ cells among CD4^+^ T cells in adult (n = 30) or cord blood (n = 3) after short-term stimulation with heat-inactivated bacteria. (*E*) Expression of CD45RA on CD4^+^CD154^+^ T cells, showing 4 representative stimulations of the same donor. (*F*) Mean (±SEM) frequencies of memory cells within CD4^+^CD154^+^ T cells. Each symbol represents an antigen-reactive population from one individual (n = 19−30). (*G*) Expression of CD45RA, TNF-α, and IL2 by CD4^+^CD154^+^ T cells after short-term PBMC stimulation with bacterial lysates. Statistics: (*B*, *C*, *F*) 1-way analysis of variance with Sidak’s multiple comparison test; (*D*) Mann-Whitney test.

**Supplementary Figure 1:**
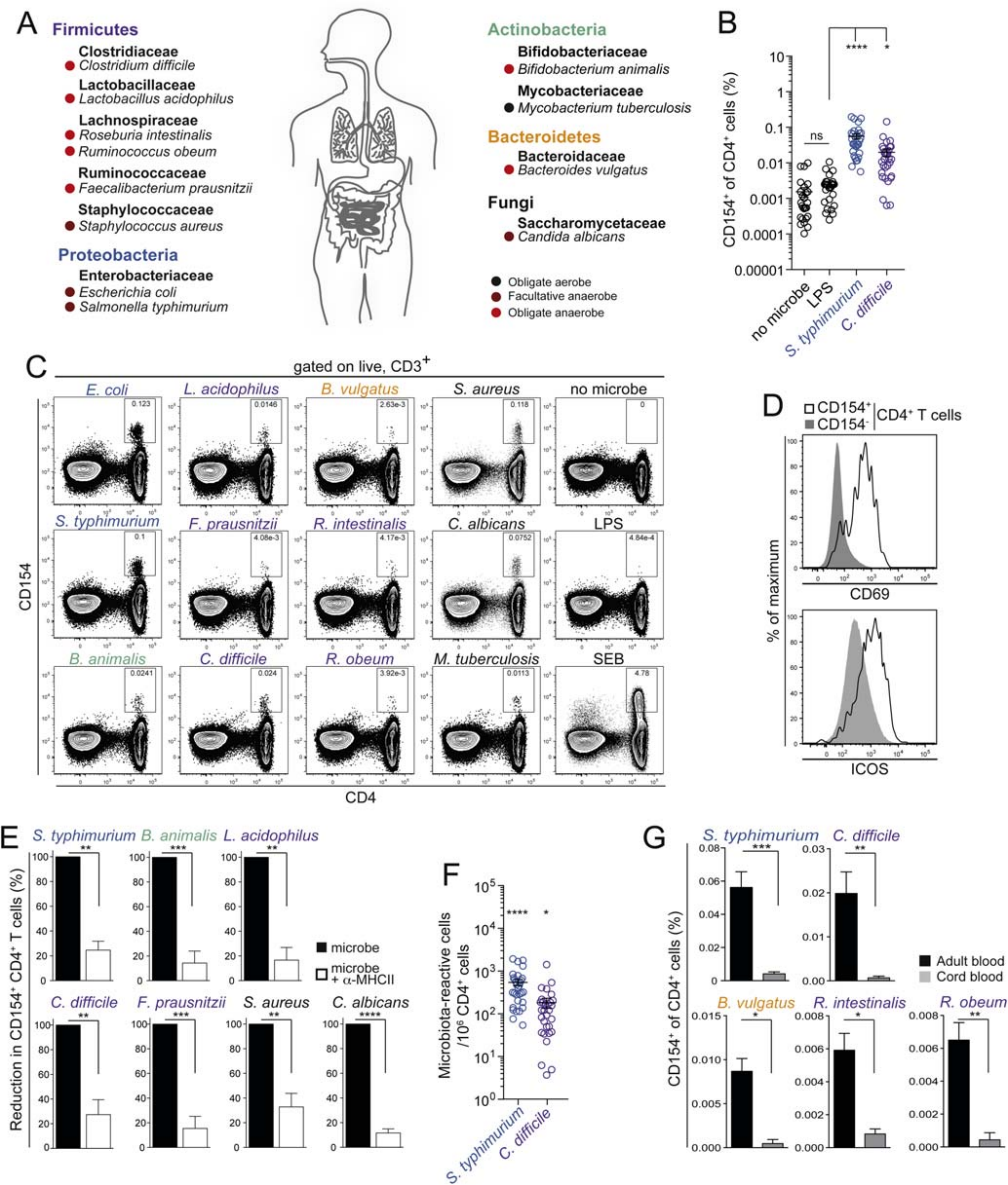
Bacterial strains used in the study and detection of enteric bacteria-specific memory CD4^+^ T cells (related to Figure 1). (*A*) Bacterial species are listed according to their family and phylum classification. *Colored circles* relate the bacteria to their gaseous requirement. (*B*) Frequencies of CD154^+^ cells among peripheral CD4^+^ T cells in adults after short-term stimulation with heat-inactivated bacteria. *Dots* represent different donors (n = 30). (*C*) Representative flow cytometric plots of different stimulations showing CD154 expression against CD4 within CD3^+^ cells after short-term stimulation with heat-inactivated bacteria. Frequencies of CD154 within CD4^+^ T cells are depicted. (*D*) Representative plot of CD69 and ICOS expression on CD4^+^CD154^+^ T cells after short-term stimulation with heat-inactivated *S typhimurium.* (*E*) PBMCs were stimulated with the indicated heat-inactivated bacteria in presence or absence of major histocompatibility complex II (MHCII) blocking antibodies and analyzed for CD154 expression. Percentage of reduction in CD154^+^ frequencies after MHCII blockade compared to control treated is depicted. Frequencies (±SEM) of 3 independent experiments are depicted (n = 5−6 independent donors). (*F*) Estimated microbiota-reactive cells per million CD4^+^ T cells in adult blood (n = 30). The numbers of microbiota-reactive T cells were calculated based on the frequencies of CD4^+^CD154^+^ T cells. Background (no microbe stimulation) was subtracted from bacterial stimulations. Significance calculated in relation to lipopolysaccharide (LPS) stimulation. (*G*) Frequencies ± SEM of CD154^+^ cells among CD4^+^ T cells in adult (n = 30) or cord blood (n = 3) after short-term stimulation with heat-inactivated bacteria. Statistics: (*E*, *G*) Mann-Whitney test; (*C*, *F*) 1-way analysis of variance with Sidak’s multiple comparison test; ns, not significant; ^∗^*P* ≤ .05; ^∗∗∗^*P* ≤ .01; ^∗∗∗^*P* ≤ .001; ^∗∗∗∗^*P* ≤ .0001.

CD154 (also known as CD40 ligand) is rapidly up-regulated by CD4^+^ T cells after antigen stimulation, irrespective of their differentiation phenotype, major histocompatibility complex alleles, or the precise nature of the antigenic epitope.^13^, ^14^ We therefore used CD154 to detect naïve and memory CD4^+^ T-cell responses among PBMCs after stimulation with heat-inactivated bacteria (Figure 1*A*). Seven aerobic and anaerobic intestinal bacterial species representing the 4 dominant gut phyla were chosen: *Escherichia coli*, *Lactobacillus acidophilus*, *Bifidobacterium animalis subsp. lactis*, *Faecalibacterium prausnitzii*, *Bacteroides vulgatus*, *Roseburia intestinalis*, and *Ruminococcus obeum* (Supplementary Figure 1*A* and Supplementary Table 1). These bacteria are common in the healthy intestine, but are altered in relative abundance during inflammation.^15^, ^16^ Furthermore, we analyzed responses to *Salmonella typhimurium* and *Clostridium difficile* due to their association with IBD.^17^, ^18^ T-cell responses to these bacteria were compared with those against well-characterized barrier surface-related microbes that drive robust Th17 responses (*Staphylococcus aureus* and *Candida albicans*) or strong Th1 responses (*Mycobacterium tuberculosis*).^19^, ^20^ The presence of a large pool of *M tuberculosis*-reactive memory Th1 cells in non-exposed individuals has been documented previously. The responses in healthy controls are directed toward non-tuberculous mycobacteria rather than toward *M tuberculosis.*^19^, ^21^, ^22^ The superantigen *Staphylococcus* enterotoxin B (SEB) was used as a positive stimulation control.

Stimulation with enteric bacteria reproducibly induced detectable numbers of CD4^+^CD154^+^ T cells in the peripheral blood. CD4^+^ T cells reactive to *S aureus*, *C albicans*, and *M tuberculosis* were generally more abundant (Figure 1*B* and Supplementary Figure 1*B* and *C*).

Activation markers CD69 and ICOS were up-regulated on activated antigen-reactive CD4^+^CD154^+^ T cells (Supplementary Figure 1*D*). Responses were major histocompatibility complex II−dependent (Supplementary Figure 1*E*), and lipopolysaccharide failed to induce CD154 expression, confirming that CD154 up-regulation was not a consequence of non-specific microbial responses (Figure 1*B* and Supplementary Figure 1*C*). Based on CD154^+^ cell frequencies, we calculated that enteric bacteria-reactive CD4^+^ T cells were present at precursor frequencies of 40−500 cells per 10^6^ circulating CD4^+^ T cells for almost all enteric bacteria surveyed (Figure 1*C* and Supplementary Figure 1*F*).

The newborn gut is primarily colonized with maternal vaginal and fecal bacteria after birth.^23^ To understand whether T-cell reactivity to microbes develops after birth, we compared CD154 expression in umbilical cord blood with adult blood after enteric bacteria stimulation. As expected, appreciable responses to microbiota were observed only in adult blood. However, CD4^+^CD154^+^ T-cell frequencies after SEB stimulation were similar between adult and cord blood (Figure 1*D* and Supplementary Figure 1*G*).

**Supplementary Figure 2:**
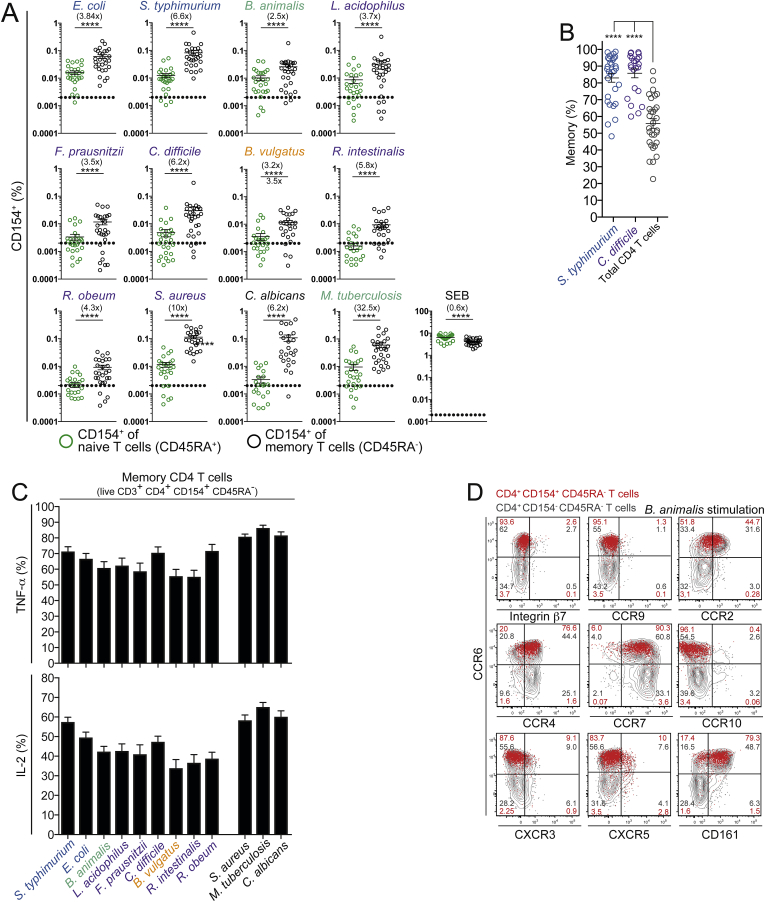
Detection and characterization of microbiota-specific memory CD4^+^ T cells (related to Figure 1 and 2). (*A*) Frequencies of CD154^+^ cells among naïve and memory peripheral CD4^+^ T cells in adults after short-term stimulation with heat-inactivated bacteria. *Dots* represent different donors (n = 30). (*B*) Percentage of memory cells within CD4^+^CD154^+^ T cells. Each *symbol* represents an antigen-reactive population from 1 individual; the *line* indicates the mean of experiments performed independently with blood obtained at different times (n = 19−30). Significance calculated in relation to total CD4^+^ T cells. (*C*) Percentages of cytokine-expressing cells among memory CD4^+^CD45RA^−^CD154^+^ T cells are shown. Frequencies (±SEM) of 27 independent donors are depicted. (*D*) Surface marker and chemokine receptor expression frequencies among CD4^+^ CD45RA^−^ CD154^+^ and CD4^+^ CD45RA^−^ CD154^−^ T cells after short-term stimulation with *B animalis*. Frequencies within CD4^+^ CD45RA^−^CD154^+^ or CD4^+^ CD45RA^−^ CD154^−^ T cells are depicted. Statistics: (*A*) Mann-Whitney tests; (*B*) 1-way analysis of variance with Sidak’s multiple comparison test; ns, not significant; ^∗^*P* ≤ .05; ^∗∗^*P* ≤ .01; ^∗∗∗^*P* ≤ .001; ^∗∗∗∗^*P* ≤ .0001.

Human memory T cells down-regulate the naïve marker CD45RA and produce cytokines more efficiently than naïve T cells.^24^, ^25^ In healthy individuals, the majority of bacteria-reactive CD4^+^ T cells had a memory phenotype (>80%, on average), indicating that they had been primed in vivo (Figure 1*E* and *F* and Supplementary Figure 2*A* and *B*). Microbiota-reactive CD4^+^CD154^+^CD45RA^−^ T cells expressed high amounts of TNF-α and IL2 when compared to CD4^+^CD154^+^CD45RA^+^ T cells (Figure 1*G* and Supplementary Figure 2*C*). Therefore, the circulating pool of memory CD4^+^ T cells contains numerous microbiota-reactive cells that arise after birth and produce cytokines, including TNF-α and IL2.

### Circulating Microbiota-Reactive CD4^+^ T Cells Express Surface Molecules That Permit Mucosal Trafficking

**Figure 2:**
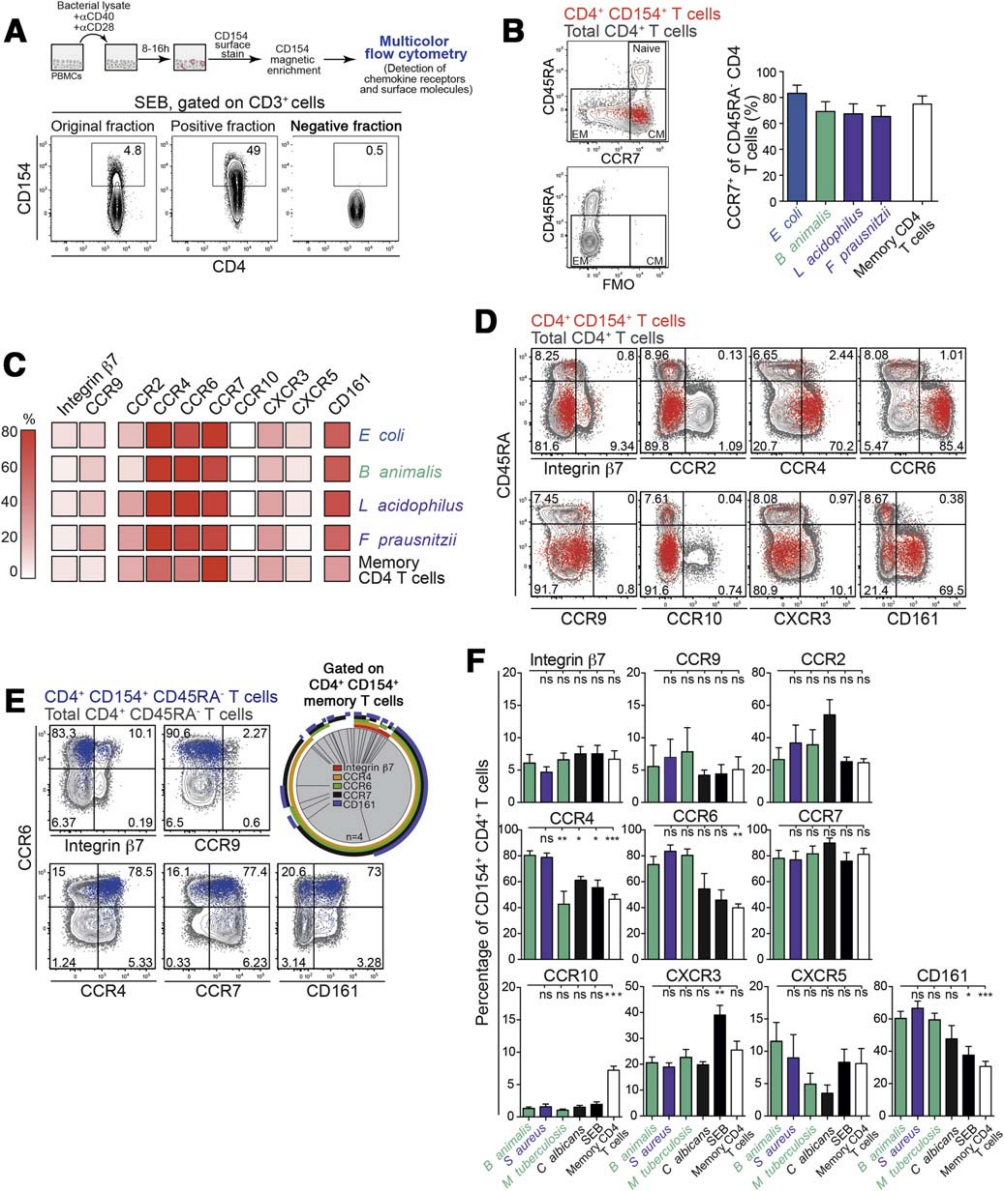
Circulating microbiota-reactive CD4^+^ T cells express several surface molecules that promote mucosal trafficking. CD4^+^CD154^+^ T cells were analyzed by flow cytometry after magnetic enrichment following short-term PBMC stimulation with heat-inactivated bacteria. (*A*) Experimental setup and enrichment efficiency after stimulation with SEB. (*B*) Representative CD45RA and CCR7 expression on enriched CD4^+^CD154^+^ T cells. Central memory T-cell frequencies are shown (*right bar graph*). Frequencies (±SEM) of 4 independent experiments are depicted with n = 8 independent donors. (*C*) *Heat map* depicting mean frequencies of surface marker and chemokine receptor expression on enriched CD4^+^CD154^+^ T cells (n = 5−8 independent donors). (*D*) Surface marker and chemokine receptor expression frequencies among CD4^+^CD154^+^ T cells after short-term stimulation with *B animalis*. Data representative of 5−8 independent donors. (*E*) Co-expression of surface molecules with CCR6 after short-term stimulation of CD4^+^CD154^+^ CD45RA^−^ T cells with *B animalis* (*left panel*). Boolean gating analysis shows each possible combination of CCR7, CCR4, CCR6, and CD161 expression (*right panel*). (*F*) Surface marker and chemokine receptor expression frequencies among total memory CD4^+^ T cells and CD4^+^CD154^+^ T cells after short-term stimulation with *B animalis, S aureus, M tuberculosis, C albicans,* or SEB*.* Frequencies (±SEM) of 4 independent experiments are depicted with n = 5−8 independent donors. Statistics: (*F*) 1-way analysis of variance with Sidak’s multiple comparison test.

Memory T cells express numerous adhesion molecules and chemokine receptors to access different tissues under steady-state and inflammatory conditions.^4^, ^26^, ^27^ For example, α4β7 integrin and chemokine receptor (CCR) 9 regulate T-cell migration to distinct parts of the gut. Blockade of α4β7 integrin has shown clinical efficacy for treating IBD, whereas CCR9 blockade yielded mixed results.^28^, ^29^ To identify homing receptors expressed by bacteria-reactive CD4^+^ T cells, we enriched CD4^+^CD154^+^ T cells using magnetic beads to visualize rare enteric-bacteria-reactive T cells, and analyzed them by flow cytometry (Figure 2*A*). Microbiota-reactive T cells had a central memory phenotype, with >60% expressing high levels of CCR7 (Figure 2*B*). Furthermore, 5%−10% of CD4^+^CD154^+^ T cells expressed the gut-homing surface markers integrin β7 and CCR9 (Figure 2*C* and *D*). Relative to total memory CD4^+^ T cells and CD4^+^CD154^−^ T cells, enteric bacteria-reactive T cells had high expression of the mucosa-homing receptors CCR4 and CCR6 (>60%), low expression of CCR10, and comparable expression of CXCR3 and CCR2 (Figure 2*D* and Supplementary Figure 2*D*). Microbiota-reactive CD4^+^ T cells also expressed high amounts of CD161, a marker enriched on Th17 cells (Figure 2*D* and *E*). The majority of memory CD4^+^CD154^+^ T cells co-expressed CCR7, CCR4, CD161, and CCR6 in various combinations, some of which also expressed integrin β7 (Figure 2*E, pie chart*). Therefore, circulating microbiota-reactive CD4^+^ T cells are equipped with several homing receptors that promote mucosal access.

When comparing gut microbiota-reactive CD4^+^ T cells with those reactive to non-enteric organisms (including *S aureus*, *M tuberculosis*, and *C albicans*), enteric bacteria-reactive T cells were partially enriched only in CCR4 expression (Figure 2*F*). The homing receptor phenotype of enteric bacteria-reactive T cells is consistent with that of T cells reactive to a broad diversity of microbes located at barrier surfaces.

### Microbiota-Reactive CD4^+^ T Cells Are Enriched in Gut Tissue

**Figure 3:**
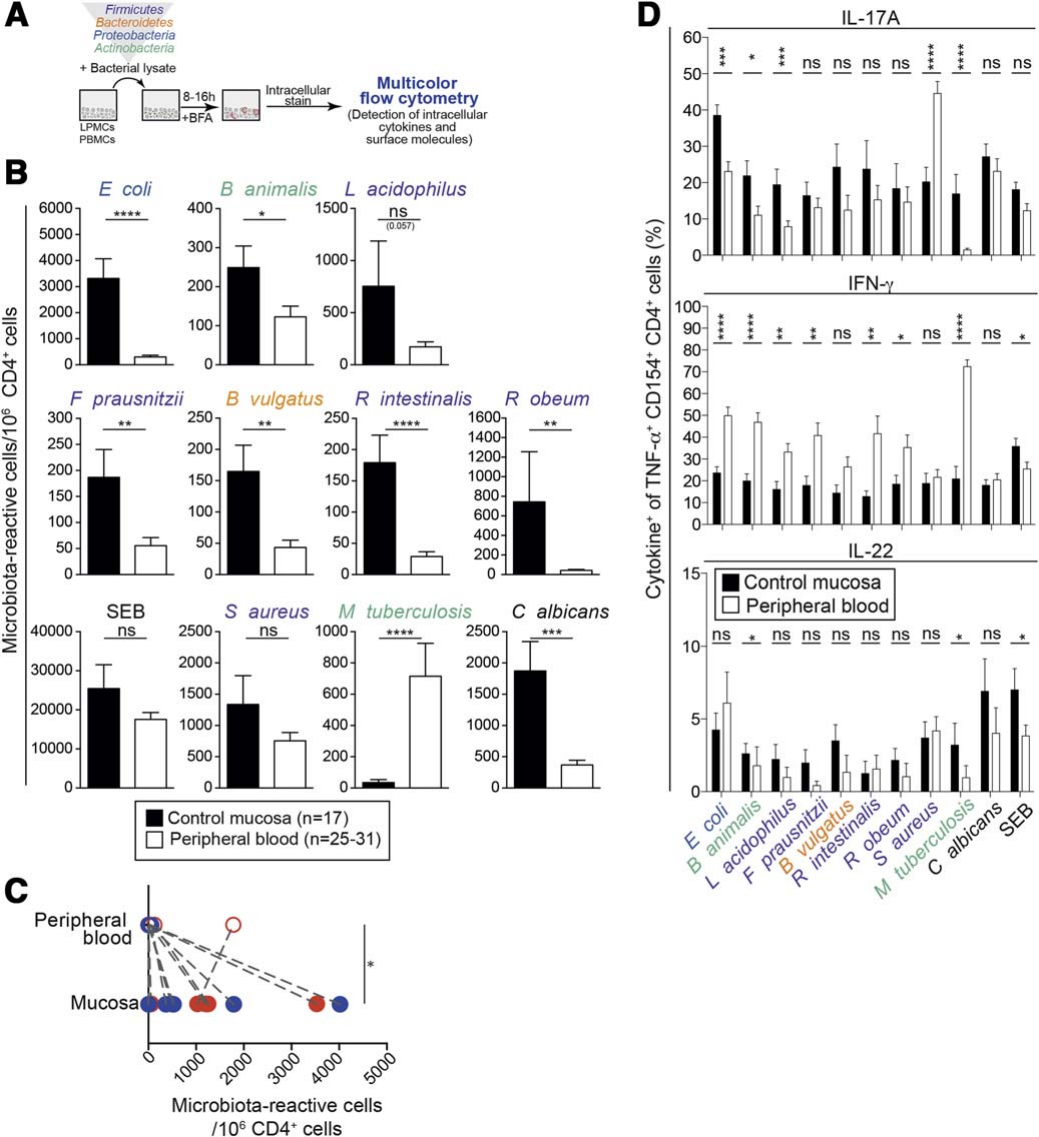
Microbiota-reactive CD4^+^ T cells are enriched in gut tissue. Lamina propria mononuclear cells (LPMCs) were isolated from non-inflamed and tumor-free surgical specimens from colorectal cancer patients. (*A*) Experimental setup. LPMCs were stimulated with heat-inactivated bacteria for 8−12 hours and assessed for intracellular expression of cytokines and CD154. (*B*) Estimated microbiota-reactive cells per million CD4^+^ T cells in adult blood and intestinal tissue from unrelated donors, based on CD154 staining (n = 17 for control mucosa; n = 25−31 for blood). (*C*) Matched LPMCs and PBMCs were stimulated with heat-inactivated *E coli* (*blue symbols*) or *S typhimurium* (*red symbols*), analyzed for CD154 expression, and compared with respect to the estimated microbiota-reactive cells per million CD4^+^ T cells. *Connected dots* represent matched samples. (*D*) Frequencies (±SEM) of IL17A, IFN-gamma, and IL22 production by CD154^+^TNF-α^+^ memory CD4^+^ T cells isolated from LPMCs or PBMCs from unrelated donors after stimulation with heat-inactivated bacteria (n = 10−23 donors). Significance calculated between the respective stimulations in control mucosa and peripheral blood. Statistics: (*B*, *D*) Mann-Whitney test; (*C*) paired *t* test.

**Supplementary Figure 3:**
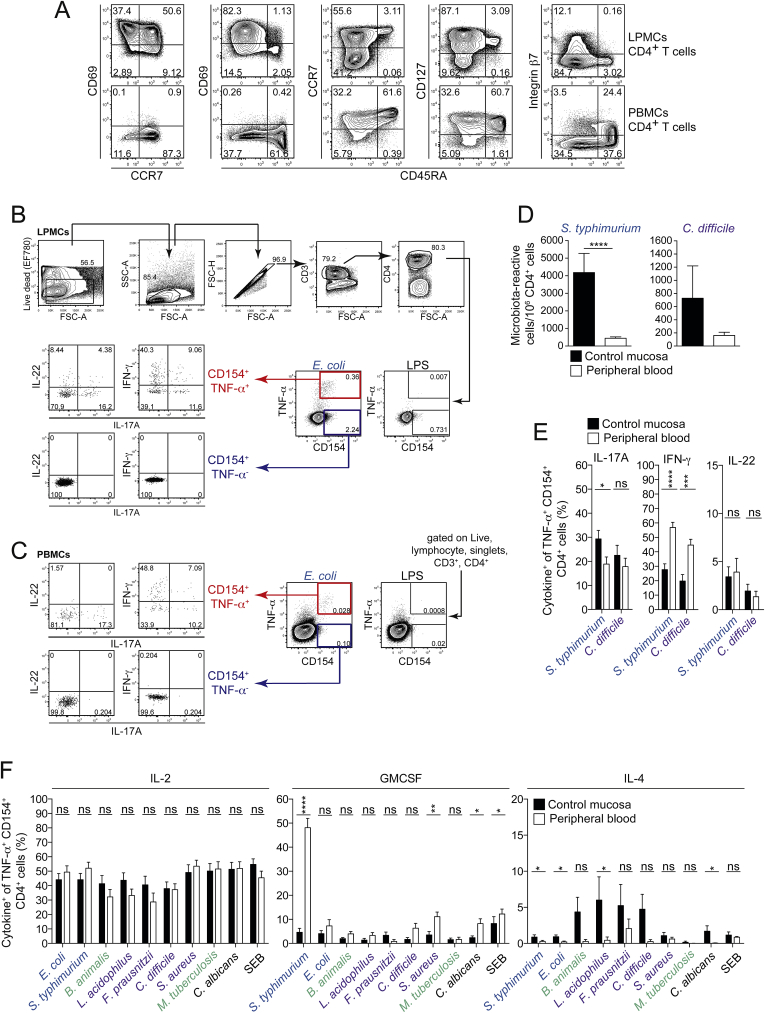
Detection and characterization of microbiota-specific CD4^+^ T cells within gut-resident memory T cells (related to Figure 3). (*A*) Representative flow cytometry plots showing CD45RA, integrin β7, CD69, CCR7, and CD127 expression in gut-resident CD3^+^CD4^+^ T cells. Data representative of 10 independent donors. (*B*, *C*) Gating strategy for identification of enteric bacteria-specific memory CD4^+^ T cells in lamina propria mononuclear cell (LPMCs) and PBMCs after stimulation with heat-inactivated *E coli* in a representative donor. (*D*) LPMCs were stimulated with heat-inactivated bacteria and analyzed for CD154 expression. Estimated gut resident microbiota-reactive cells per million CD4^+^ T cells compared with adult blood is depicted (n = 17 for control mucosa; n = 25−31 for blood). (*E*, *F*) Cytokine production by CD4^+^CD154^+^TNF-α^+^ T cells after short-term stimulation with the indicated heat-inactivated bacteria. Frequencies (±SEM) of 10−23 independent donors. Significance calculated between the respective stimulations in control mucosa and peripheral blood. Statistics: (*D*, *E*, *F*) Mann-Whitney test; ns, not significant; ^∗^*P* ≤ .05; ^∗∗^*P* ≤ .01; ^∗∗∗^*P* ≤ .001; ^∗∗∗∗^*P* ≤ .0001.

The gut harbors >3 × 10^10^ CD4^+^ T cells, but their specificity is unknown.^4^, ^5^ We therefore estimated the abundance of human microbiota-reactive CD4^+^ T cells in the gut by examining non-inflamed colon specimens using the CD154 assay (Figure 3*A*). Lamina propria CD4^+^ T cells showed a dominant effector memory and central memory phenotype and expressed both tissue-resident and gut-related markers, with 80% of cells being CD69^+^ (Supplementary Figure 3*A*).

We next stimulated lamina propria mononuclear cells with microbial lysates or SEB. We combined intracellular CD154 detection with TNF-α staining to increase assay sensitivity, as lamina propria CD4^+^ T cells expressed low amounts of CD154 without stimulation (Supplementary Figure 3*B* and *C*). Compared with peripheral blood frequencies of unrelated donors, there were similar frequencies of *S aureus* and SEB reactivity, and reduced *M tuberculosis*-reactivity in the gut. However, gut CD4^+^ T cells were enriched in reactivity toward intestinal bacteria and *C albicans* (Figure 3*B* and Supplementary Figure 3*D*). Bacteria-reactive cells comprised 150−4000 cells per 10^6^ gut-resident memory CD4^+^ T cells for all enteric bacteria tested. Given that peripheral blood contained 40−500 bacteria-reactive cells per 10^6^ memory CD4^+^ T cells (for each bacteria tested), this suggests that bacteria-reactive T cells are 3- to 8-fold more frequent in gut tissue as compared with those in circulation. The strong enrichment of *S typhimurium* and *E coli* reactivity in the gut was confirmed by assessing CD154 and TNF-α expression in CD4^+^ T cells from donor-matched blood and intestinal tissue (Figure 3*C*). Because the gut harbors up to 3 × 10^10^ memory T cells (vs 5−10 × 10^9^ in blood),^4^ many of which are bacteria-reactive, the absolute number of gut-resident microbiota-reactive CD4^+^ T cells is likely to be at least 10 times greater than that in peripheral blood.

Gut-resident bacteria-reactive (CD154^+^TNF-α^+^) T cells produced high amounts of IFN-gamma, IL17A, and IL2, while production of IL22, granulocyte macrophage colony-stimulating factor, and IL4 was generally low (Figure 3*D* and Supplementary Figure 3*E* and *F*). Interestingly, lamina propria T cells showed increased IL17A expression and reduced IFN-gamma production relative to cells with similar reactivity in peripheral blood (Figure 3*D* and Supplementary Figure 3*E*).

### Enteric Bacteria-Reactive CD4^+^ T Cells Are Clonally Diverse

**Figure 4:**
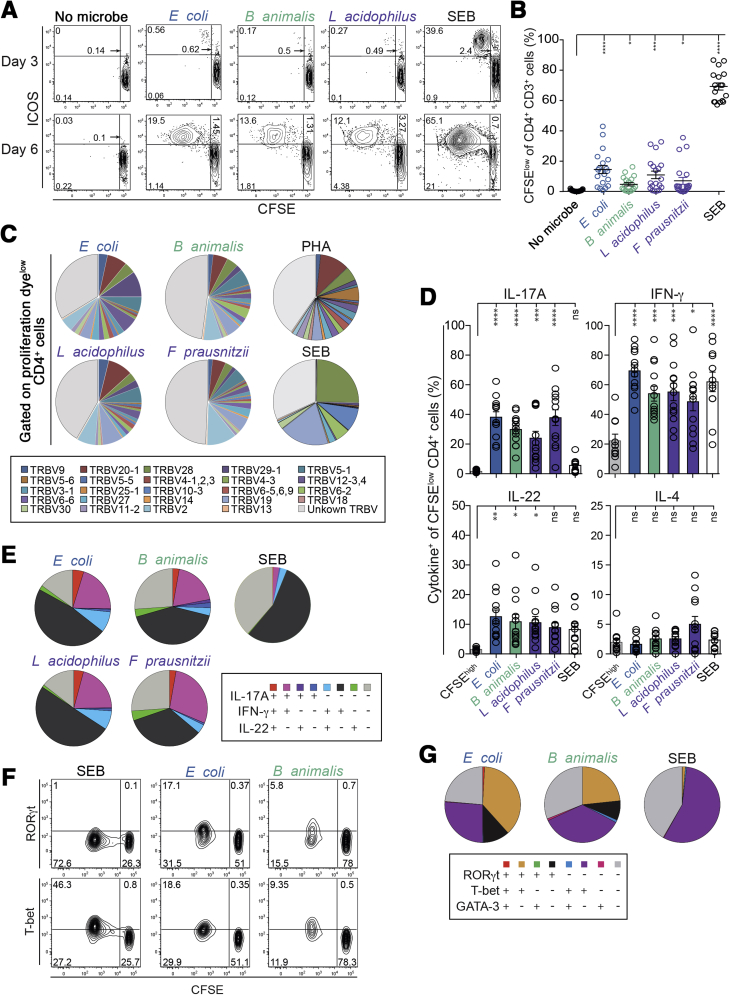
Microbiota-reactive memory CD4^+^ T cells are clonally diverse and functionally heterogeneous. Memory CD4^+^ T cells were labeled with CFSE or violet proliferation dye (VPD)-450 and stimulated with heat-inactivated bacteria in the presence of autologous monocytes. (*A*) CFSE profiles and inducible T-cell costimulator (ICOS) expression on days 3 and 6 of stimulation in a representative donor. (*B*) Percentage of CFSE^low^ CD4^+^ T cells of each individual donor (n = 18). (*C*) Pie charts showing TCR Vβ expression by proliferating VPD^low^ cells measured by Vβ antibody staining on day 7 of stimulation. Average of 3 independent donors is depicted. TCR Vβ usage of the different reactivities of 3 independent donors is summarized in Supplementary Table 2. (*D*) Mean (±SEM) cytokine production frequencies of proliferating CFSE^low^ cells and non-activated CFSE^high^ cells after phorbol myristate acetate/ionomycin stimulation (n = 12−13 independent donors). (*E*) Boolean gating analysis showing each possible combination of IL17A, IFN-gamma, and IL22 production by CFSE^low^ proliferating cells. Data from 9 independent donors. (*F*, *G*) RAR-related orphan receptor γt (RORγt) and T-box expressed in T cells (T-bet) expression in proliferating CFSE^low^ cells measured by intracellular staining on day 7 of stimulation. (*G*) Boolean gating analysis showing each possible combination of RORγt, T-bet, and GATA-binding factor-3 production by CFSE^low^ proliferating cells. Data from 3 independent donors. Statistics: (*B*) 1-way analysis of variance with Sidak’s multiple comparison test; (*D*) 1-way analysis of variance with Bonferroni’s multiple comparison test.

**Supplementary Figure 4:**
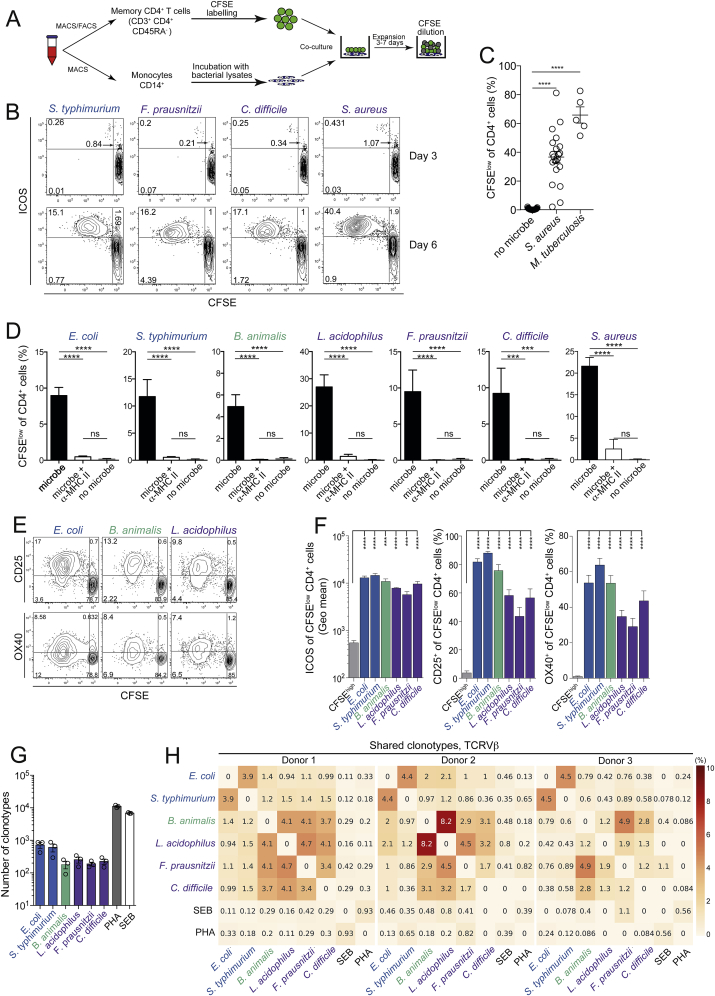
Expansion and detection of microbiota-specific memory CD4^+^ T cells (related to Figure 4). Total memory CD4^+^ T cells were isolated, labeled with CFSE, and cultured with autologous irradiated monocytes in the presence or absence of the indicated heat-inactivated bacteria and blocking antibodies to major histocompatibility complex II (MHCII). (*A*) Experimental setup. (*B*) Shown are the CFSE profiles and ICOS expression on days 3 and 6 of stimulation in a representative donor. (*C*) Frequencies (±SEM) of CFSE^low^ proliferating cells after stimulation with *S aureus* and *M tuberculosis* (n = 5−21). Data representative of 5−10 independent experiments. Each *dot* represents an independent donor. (*D*) Frequencies (±SEM) of CFSE^low^ proliferating CD4^+^ T cells in presence or absence of anti-MHCII. Data representative of 2 independent experiments. (*E, F*) CFSE profiles and expression of CD25 and OX40 on day 6 of stimulation in a representative donor. (*F*) Geometric mean and frequencies (±SEM) of ICOS, CD25, and OX40 expression on CFSE^low^CD4^+^ T cells on day 6. (*G*) Number of unique TCRβ clonotypes detected in bacteria-reactive CD4^+^ T cells. TCR Vβ sequencing was performed from 3 independent donors. Each *circle* represents an independent donor. TCR Vβ usage of the different reactivities of 3 independent donors is summarized in Supplementary Table 3. (*H*) *Heat map* showing the frequency of shared clonotypes between different bacteria-reactive CD4^+^ T-cell responses. SEB and phytohemagglutinin (PHA) expanded memory T cells were used as controls. Data from 3 independent donors. The CDR3 sequences are summarized in Supplementary Table 4. Statistics: (*C*, *D*, *F*) 1-way analysis of variance with Sidak’s multiple comparison test; ns, not significant; ^∗^*P* ≤ .05; ^∗∗^*P* ≤ .01; ^∗∗∗^*P* ≤ .001; ^∗∗∗∗^*P* ≤ .0001.

To assess the clonal diversity of circulating bacteria-reactive memory CD4^+^ T cells, we expanded CFSE-labeled CD4^+^ T cells using whole bacteria and autologous irradiated monocytes as antigen presenting cells (Supplementary Figure 4*A*).^20^ *S aureus-*, *M tuberculosis-*, and SEB-reactive T cells served as controls. Antigen-reactive T cells proliferated in a major histocompatibility complex II−dependent manner and were readily detectible after 3−6 days (Figure 4*A* and Supplementary Figure 4*B−D*). Proliferating cells expressed several activation markers including ICOS, CD25, and OX40 (Figure 4*A* and Supplementary Figure 4*B*, *E*, and *F*). Consistent with the CD154 assay, *S aureus*, *M tuberculosis*, and SEB strongly induced T-cell proliferation (Figure 4*A* and *B* and Supplementary Figure 3*B* and *C*).

**Supplementary Figure 5:**
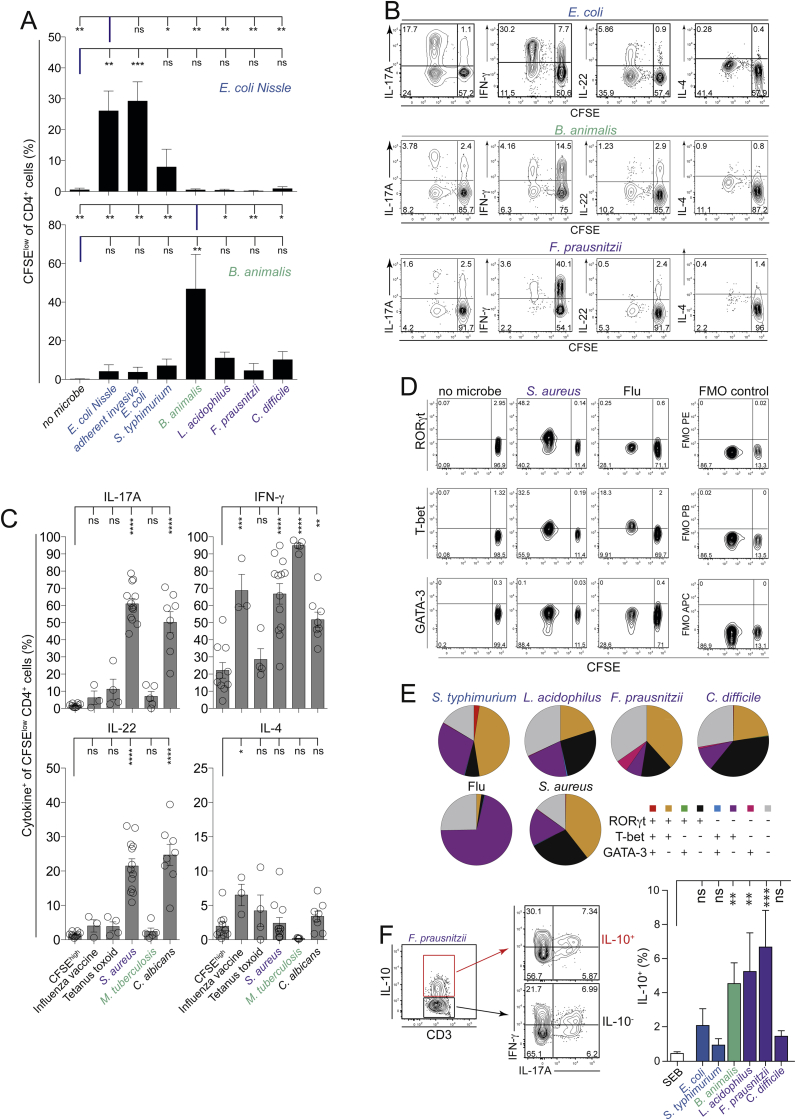
Cross-reactivity, cytokine production, and transcription factor expression by antigen-reactive memory CD4^+^ T cells (related to Figure 5). (*A*) CD154^+^ memory CD4 T cells from PBMCs were sorted after short-term stimulation with *E coli* and *B animalis* lysates and expanded them for 10−14 days with CD3/CD28 and IL2. The *E coli Nissle*- and *B animalis−*reactive T cell lines were CFSE-labeled and then co-incubated with autologous monocytes loaded with the various bacterial lysates. Frequencies (±SEM) of CFSE^low^ proliferating cells after 5 days of stimulation with the indicated bacteria are shown. Data from 3−6 independent donors. (*B*−*F*) Total memory CD4^+^ T cells were isolated, labeled with CFSE and cultured with autologous irradiated monocytes in the presence of the indicated heat-inactivated bacteria or antigens. (*B*, *C*) Production of cytokines by proliferating CFSE^low^ cells measured by intracellular staining after phorbol myristate acetate (PMA)/ionomycin stimulation on day 7 of primary stimulation with monocytes and heat-inactivated bacteria. Frequencies (±SEM) of 3−13 independent donors are depicted. Each *dot* represents an independent donor. (*D*, *E*) Related orphan receptor γt (RORγt), T-box expressed in T cells (T-bet), and GATA-binding factor-3 (GATA-3) expression on day 7 of primary stimulation with monocytes and influenza seasonal vaccination or heat-inactivated *S aureus* in a representative donor. (*E*) Boolean gating analysis showing each possible combination of RORγt, T-bet, and GATA-3 production by CFSE^low^ proliferating cells. Data generated from 3 independent donors. (*F*) Production of IL10 by expanded CFSE^low^ CD4^+^ cells after PMA/ionomycin stimulation. Frequencies (±SEM) and IFN-gamma/IL17A co-expression within IL10^+^ and IL10^−^ cells are depicted from 5−8 independent donors. Statistics: (*A, C*) 1-way analysis of variance with Sidak’s multiple comparison test; (*F*) Kruskal-Wallis test with Dunn’s multiple comparison; ns, not significant; ^∗∗∗^*P* ≤ .001; ^∗∗∗∗^*P* ≤ .0001.

Flow cytometry analysis revealed a diverse TCR Vβ repertoire in bacteria-reactive T cells, similar to polyclonal stimulation with phytohemagglutinin but different to stimulation with SEB, which is known to activate a restricted Vβ repertoire (Figure 4*C*).^30^ To directly assess the clonal diversity of bacteria-reactive CD4^+^ T cells, we isolated CFSE^low^ bacteria-reactive memory T cells and assessed TCR Vβ clonotypes by multiplex polymerase chain reaction and deep sequencing. One hundred and fifty to eight hundred clonotypes were detected for each reactivity (Supplementary Figure 4*G*). The largest clonal diversity was detected among *E coli*- and *S typhimurium−*reactive cells, consistent with frequencies observed in the CD154 assay (see Figure 1*B* and *C* and Supplementary Figure 1*B* and *F*). While closely related species (eg, *E coli* vs *S typhimurium*) had 3%−8% overlap in T-cell clonotypes, little clonotype sharing was observed between T cells reactive to more distantly related bacteria (Supplementary Figure 3*H*). Indeed, *E coli*- and *B animalis−*reactive CD4^+^ T-cell lines were strongly restimulated when cultured with autologous monocytes loaded with *E coli* or *B animalis* lysates, respectively*.* In contrast, *E coli*−reactive T cells responded weakly to the closely related *S typhimurium*, while *B animalis*−reactive T cells responded weakly to *L acidophilus*, *F prausnitzii*, and *C difficile* (Supplementary Figure 5*A*). These data confirm the low degree of cross-reactivity predicted from TCR Vβ sequencing.

### Microbiota-Reactive Memory CD4^+^ T Cells Are Functionally Heterogeneous and Produce Barrier-Promoting Cytokines

To functionally characterize circulating microbiota-reactive memory cells, we analyzed CFSE^low^ cells using flow cytometry after stimulation with enteric bacteria for 6 days. Enteric microbiota-reactive cells produced Th1- and Th17-related cytokines, including IFN-gamma, IL17A, and IL22, but only low amounts of the Th2 cytokine IL4, comparable to cells reactive toward *S aureus* or *C albicans* (Figure 4*D* and Supplementary Figure 5*B* and *C*). In contrast, memory T cells reactive toward SEB, *M tuberculosis*, influenza vaccine components, or tetanus toxoid showed a polarized Th1 profile with low expression of IL17A (Figure 4*D* and Supplementary Figure 5*B* and *C*). Boolean gating revealed a high degree of functional heterogeneity in expanded microbiota-reactive memory T cells, with frequent co-expression of IL17A, IL22, and IFN-gamma (Figure 4*E*). Bacteria-reactive cells co-expressed the transcription factors RAR-related orphan receptor γt and T-box expressed in T cells, which are characteristic of Th17 and Th1 cells, respectively (Figure 4*F* and *G* and Supplementary Figure 5*D* and *E*). Intriguingly, a subset of CD4^+^ T cells reactive to *F prausnitzii*, *L acidophilus*, or *B animalis* produced the immunoregulatory cytokine IL10 in addition to IFN-gamma and IL17A (Supplementary Figure 5*F*)*.* Compared to T cells that are reactive toward *M tuberculosis* or vaccine antigens, enteric microbiota-reactive T cells are functionally distinct and produce barrier-promoting and immunoregulatory cytokines.

### Microbiota-Reactive Memory T Cells Promote Intestinal Stromal and Epithelial Cell Activation

**Figure 5:**
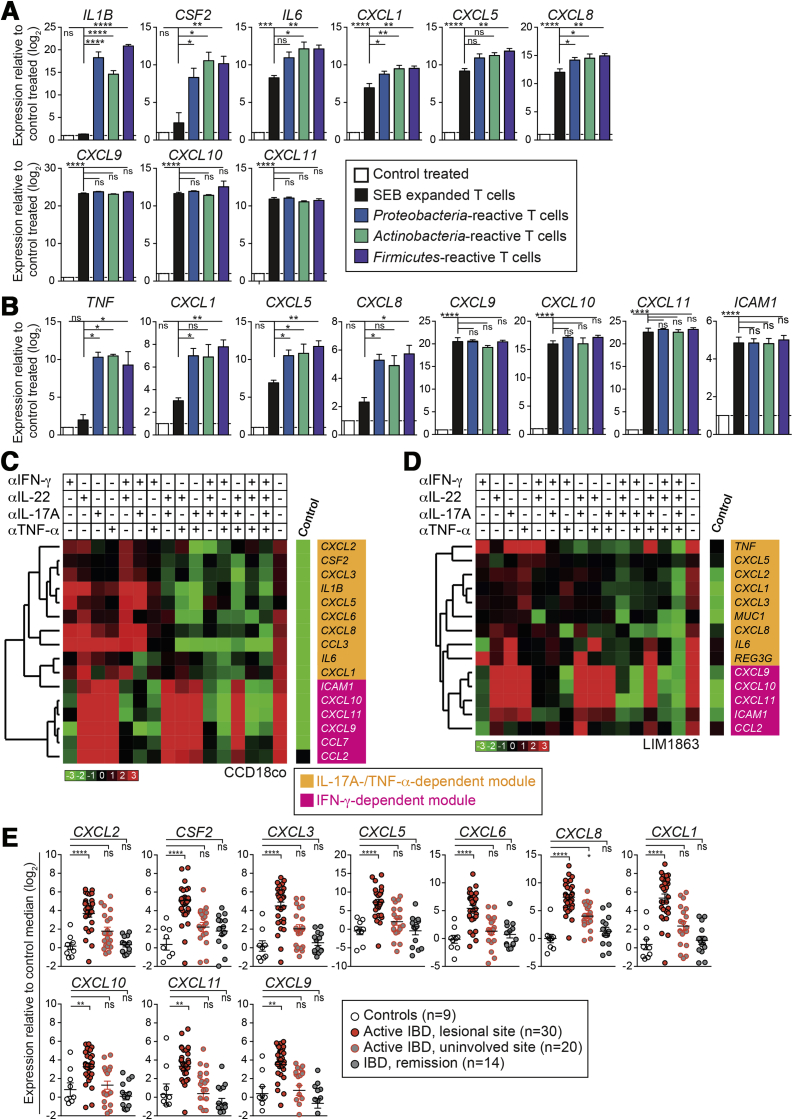
Microbiota-reactive memory T cells promote intestinal stromal and epithelial cell activation. Healthy donor memory CD4^+^ T cells from peripheral blood were labeled with CFSE and stimulated with heat-inactivated bacteria in the presence of autologous monocytes. CD4^+^CFSE^low^ICOS^high^cells were fluorescence-activated cell−sorted on day 7 and expanded for 10−14 days with anti-CD3/CD28 beads. Expanded cells were stimulated at equal numbers with phorbol myristate acetate (PMA)/ionomycin for 24 hours to produce conditioned supernatants. (*A, B*) Cell-free supernatants from different T-cell specificities were used to stimulate CCD18Co intestinal myofibroblasts and LIM1863 colon epithelial cells. Gene expression in stimulated cells was measured by quantitative polymerase chain reaction (qPCR) and normalized to control treatment (media containing PMA/ionomycin alone). Results of independent stimulations were pooled together into the following categories: *Proteobacteria*-reactive T cells (*S typhimurium*− and *E coli*−reactive)*; Actinobacteria*-reactive T cells (*B animalis-*reactive); *Firmicutes*-reactive T cells (*F prausnitzii*− and *L acidophilus*−reactive). Data are from 3 independent T-cell donors. (*C*, *D*) Supernatants from *E coli*−reactive CD4^+^ T cells were used to stimulate CCD18Co (*C*) or LIM1863 (*D*) cells. Supernatants were pretreated with 1 or more cytokine-neutralizing antibodies as indicated. Gene expression was median-normalized, log_2_ transformed, and plotted as a heat map. Data representative of 2−3 independent experiments. (*E*) qPCR analysis of mucosal biopsies from the Oxford IBD cohort, categorized by endoscopic assessment of disease activity. Demographic and clinical characteristics of IBD patients are summarized in Supplementary Table 5. Statistics: (*A, B*, *E*) 1-way analysis of variance with Sidak’s multiple comparison test.

During periods of epithelial damage and exposure to commensals, activation of microbiota-reactive memory T cells could promote protective immune responses. To assess their tissue-modulating capabilities, cell-free supernatants of microbiota-reactive memory T cells were used to stimulate CCD18Co intestinal myofibroblasts and LIM1863 colonic epithelial cells. CCD18Co and LIM1863 cells were then assessed for expression of various immune response genes that were selected a priori to represent responses to major T-cell−derived cytokines. Both cell types responded by expressing several cytokine and chemokine genes known to be induced by IL17A (including *IL1B*, *CSF2*, *IL6*, *CXCL1*, and *CXCL8*), as well as IFN-gamma−inducible genes, including *CXCL9*, *CXCL10*, and *CXCL11* (Figure 5*A* and *B*).^31^ Conversely, supernatants from SEB-stimulated memory T cells (which produce little IL17A) mainly induced IFN-gamma−dependent genes. Thus, stimulation of non-hematopoietic intestinal cells by microbiota-reactive T cells may promote recruitment and activation of myeloid cell populations to facilitate pathogen control and tissue repair.

We next assessed the effects of individual cytokines in *E coli*−reactive T-cell supernatants using combinations of neutralizing antibodies. This experiment revealed distinct IFN-gamma− and IL17A-/TNF-α−dependent groups of response genes in both intestinal epithelial cells and fibroblasts. IFN-gamma blockade strongly reduced expression of several chemokine genes, including *CXCL9*, *CXCL10*, *CXCL11*, *CCL2*, and *CCL7* (IFN-gamma−dependent module; Figure 5*C* and *D*). Intriguingly, single blockade of IL17A, IL22, or TNF-α did not affect stromal or epithelial cell activation (Figure 5*C* and *D*). However, combined blockade of IL17A and TNF-α influenced a large number of genes, including *CSF2*, *IL1B*, *TNF*, *CXCL1*, *CXCL8*, *CXCL5*, *CXCL6*, and *CCL20 (*IL17A/TNF-α−dependent module). Triple blockade of IFN-gamma, IL17A, and TNF-α completely inhibited stromal and epithelial cell activation. IL22 blockade did not affect cytokine or chemokine production, but attenuated induction of the antimicrobial peptide *REG3G* in LIM1863 cells. Given that the products of T-cell−stimulated stromal and epithelial cells are highly expressed in the inflamed mucosa of IBD patients (Figures 5*E* and 7*D*), this signature might reflect the activation of microbiota-reactive T cells after epithelial disruption, a key feature of IBD.

### Microbiota-Reactive CD4^+^ T Cells in Inflamed Intestinal Tissue Show a T-Helper 17−Skewed Phenotype in Patients With Inflammatory Bowel Disease

**Figure 6:**
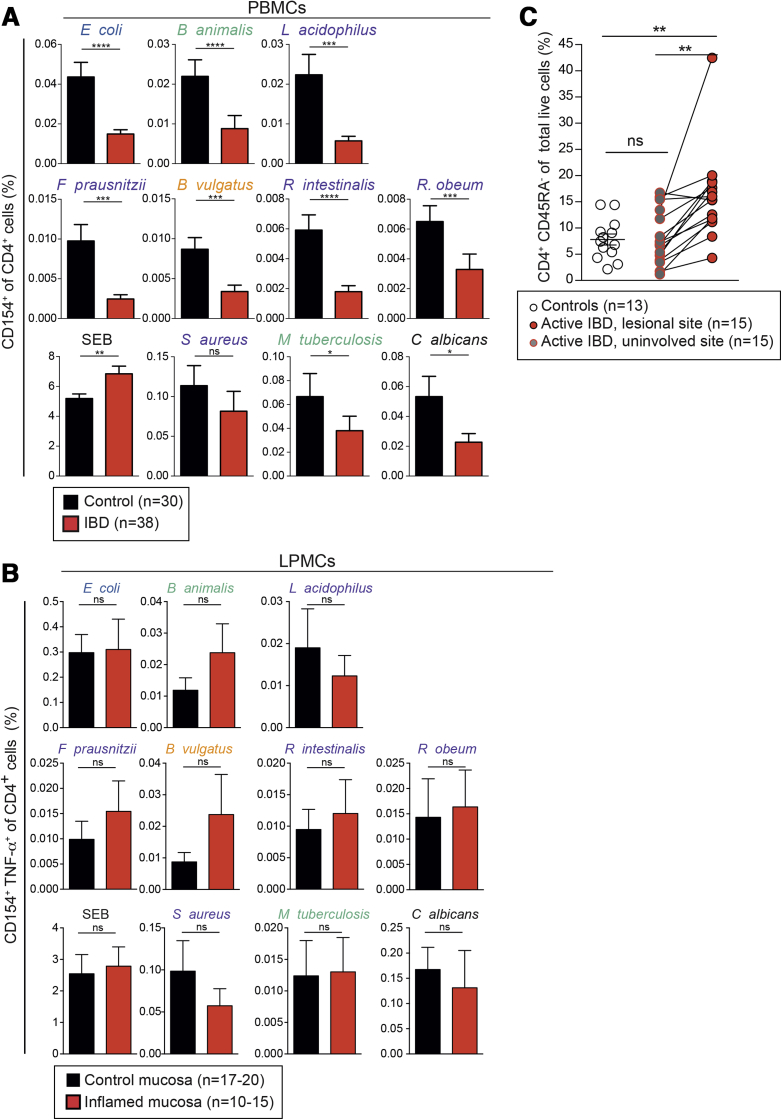
Abundance of circulating and gut-resident enteric bacteria-reactive CD4^+^ T cells in IBD. (*A*) PBMCs from healthy donors or IBD patients were stimulated with the indicated heat-inactivated bacteria and analyzed for CD154 expression. Frequencies (±SEM) of CD154^+^ cells among CD4^+^ T cells are depicted (n = 30−38). Demographic and clinical characteristics of IBD patients are summarized in Supplementary Table 6. (*B*) Lamina propria mononuclear cells (LPMCs) were isolated from inflamed surgical specimens from IBD patients or non-inflamed and tumor-free surgical specimens from colorectal cancer patients. Isolated LPMCs were stimulated with the indicated heat-inactivated bacteria and analyzed for CD154 expression. Frequencies (±SEM) of CD154^+^ cells among CD4^+^ T cells are depicted (n = 10−20). (*C*) LPMCs from endoscopic intestinal biopsies were obtained from healthy controls or matched lesional and non-lesional sites of IBD patients from an independent Oxford cohort (n = 12 controls and 17 IBD). *Dots* represent frequencies of CD45RA^−^CD4^+^ memory T cells among total live LPMCs from different donors; *connected dots* represent matched biopsies. Demographic and clinical characteristics of IBD patients are summarized in Supplementary Table 7. Statistics: (*A, B, C*) Mann-Whitney test; (*C*) 1-way analysis of variance with Sidak’s multiple comparison test.

**Supplementary Figure 6:**
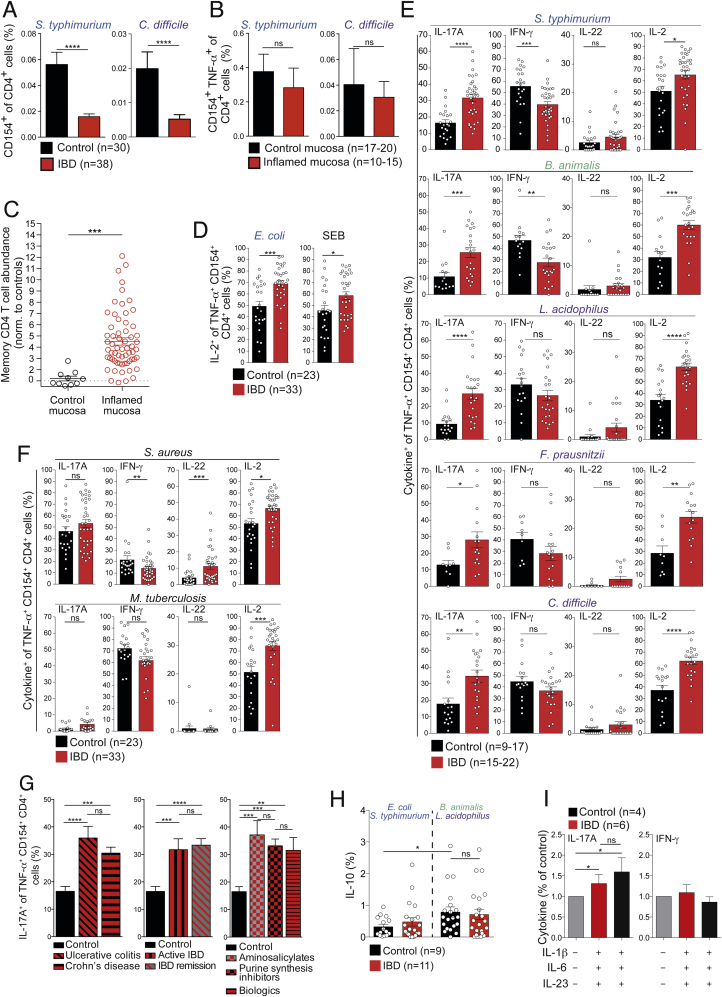
Functional characteristics of enteric bacteria-reactive CD4^+^ T cells in IBD (related to Figure 7). (*A*) PBMCs isolated from healthy donors or IBD patients were stimulated with the indicated heat-inactivated bacteria and analyzed for CD154 expression. Frequencies (±SEM) of reactive CD154^+^ cells among CD4^+^ T cells in peripheral blood are depicted (n = 30−38). Demographic and clinical characteristics of IBD patients are summarized in Supplementary Table 6. (*B*) Lamina propria mononuclear cell (LPMCs) were isolated from inflamed surgical specimens from IBD patients or non-inflamed and tumor-free surgical specimens from colorectal cancer patients. Isolated LPMCs were stimulated with the indicated heat-inactivated bacteria and analyzed for CD154 expression. Frequencies (±SEM) of reactive CD154^+^ TNF-α^+^ cells among CD4^+^ T cells in peripheral blood are depicted (n = 10−20). (*C*) Microarray analysis of intestinal biopsies obtained at endoscopy (n = 6 controls, 24 ulcerative colitis (UC) and 37 Crohn’s disease (CD); Gene Expression Omnibus entry GSE16879). The relative abundance of memory CD4^+^ T cells in control and inflamed IBD tissue was estimated in silico using CIBERSORT (see Supplementary Methods for details). (*D−F*) PBMCs isolated from healthy and IBD patients were stimulated with the indicated heat-inactivated bacteria or SEB and analyzed for CD154 expression and intracellular cytokine expression. Demographic and clinical characteristics of IBD patients are summarized in Supplementary Table 6. (*D*) IL2 expression in CD154^+^ TNF-α^+^ memory CD4^+^ T cells after stimulation with the indicated heat-inactivated bacteria or SEB. Frequencies (±SEM) of 23-33 independent donors. Each dot represent an independent donor. (*E, F*) IL17A, IFN-gamma, IL22, and IL2 expression in CD154^+^ TNF-α^+^ memory CD4^+^ T cells after stimulation with the indicated heat-inactivated bacteria. Each *dot* represents an independent donor. Demographic and clinical characteristics of IBD patients are summarized in Supplementary Table 6. (*G*) IL17A expression in CD154^+^ TNF-α^+^ memory CD4^+^ T cells after stimulation with *S typhimurium*. IBD patients were categorized by disease phenotype (UC, CD), disease activity according to clinical notes, and current treatment. (*H*) Total memory CD4^+^ T cells were isolated from healthy controls or IBD patients blood, labeled with CFSE and cultured with autologous irradiated monocytes in the presence of the indicated heat-inactivated bacteria or antigens. Production of IL10 by expanded CFSE^low^ CD4^+^ cells after phorbol myristate acetate (PMA)/ionomycin stimulation on day 7 of stimulation. Frequencies (±SEM) are depicted from 9−11 independent donors. (*I*) CD4^+^CD45RO^+^CD45RA^−^CD25^−^CD8^−^ memory CD4^+^ T cells were isolated from healthy controls or IBD patients blood, labeled with CFSE, and stimulated with autologous monocytes pulsed with *B animalis* in the presence or absence of the indicated cytokines. Data represent mean (±SEM) fold changes in IL17A or IFN-gamma expression frequencies relative to cells expanded without cytokines. Statistics: (*A−E, G−H*) Mann-Whitney test; (*F*) 1-way analysis of variance with Sidak’s multiple comparison test; ns, not significant; ^∗^*P* ≤ .05; ^∗∗^*P* ≤ 0.01; ^∗∗∗^*P* ≤ .001; ^∗∗∗∗^*P* ≤ .0001.

IBD is thought to arise in part from aberrant adaptive immune responses to microbiota.^8^ Human CD4^+^ T cells in IBD have been functionally characterized mainly by polyclonal stimulation.^32^, ^33^, ^34^ Therefore, we evaluated microbiota-reactive CD4^+^ T-cell responses in IBD patients using the CD154 detection approach. Circulating microbiota-reactive CD4^+^ T-cell frequencies were decreased in IBD patients compared with healthy donors, which might reflect their selective recruitment to the inflamed gut (Figure 6*A* and Supplementary Figure 6*A*). However, intestinal memory CD4^+^ T cells from IBD patients did not display reciprocally higher frequencies of microbial specificity (Figure 6*B* and Supplementary Figure 6*B*). We next calculated the frequency of memory CD4^+^ T cells in inflamed mucosae using flow cytometry. Memory CD4^+^ T cells were present at higher frequencies in inflamed tissue from IBD patients compared with tissue from matched non-lesional sites of IBD patients and healthy controls (Figure 6*C*). These findings were confirmed using a previously published bioinformatics approach known as CIBERSORT in an independent cohort^35^ (Supplementary Figure 6*C*). Based on both approaches, memory CD4^+^ T cells are typically 2- to 4-fold more frequent in inflamed tissue from IBD patients compared with tissue from healthy controls. Because inflamed tissue contains a higher abundance of memory CD4^+^ T cells than healthy mucosa, it can be inferred that gut-resident microbiota-reactive CD4^+^ T cells are similarly enriched in patients with active IBD (Figure 6*C*, Supplementary Figure 6*C*).

**Figure 7:**
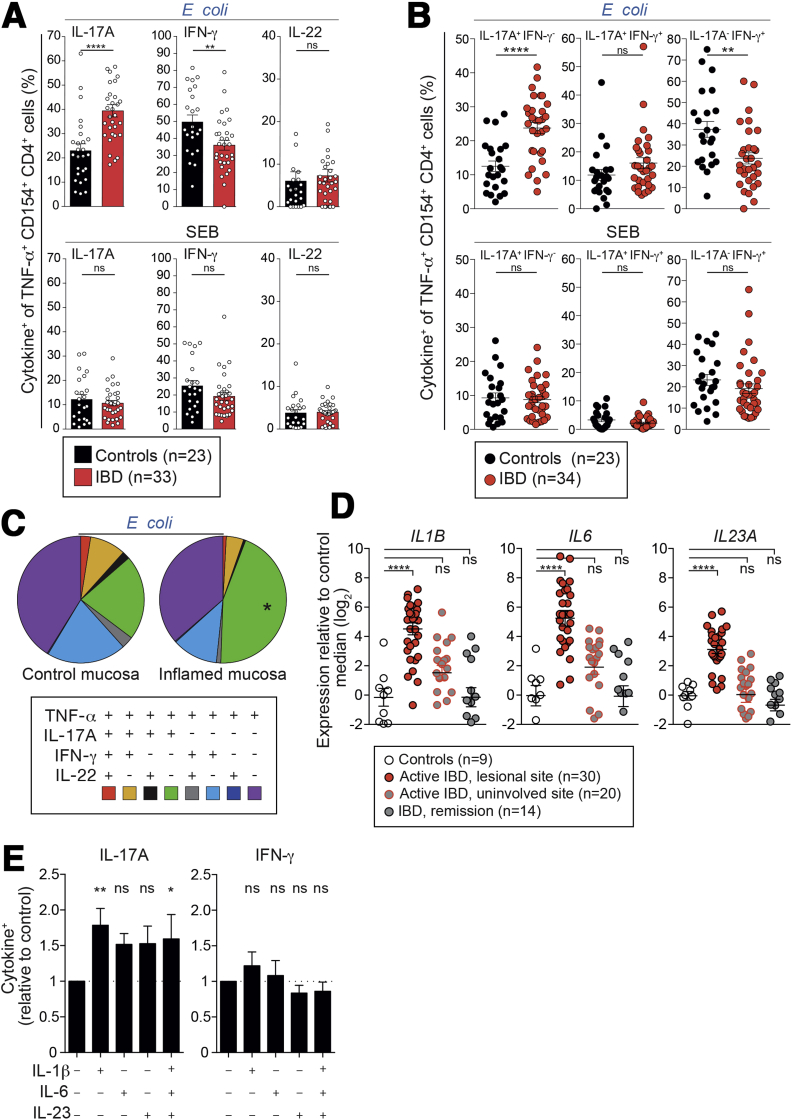
Microbiota-reactive CD4^+^ T cells show a Th17-skewed phenotype in IBD patients. (*A, B*) PBMCs isolated from healthy donors and IBD patients were stimulated with heat-inactivated bacteria or SEB and analyzed for intracellular CD154 and cytokine expression. (*A*) Frequencies (±SEM) of IL17A, IFN-gamma, and IL22 expression in CD154^+^TNF-α^+^ memory CD4^+^ T cells (n = 23−33 independent donors). Demographic and clinical characteristics of IBD patients are summarized in Supplementary Table 6. (*B*) Frequencies (±SEM) of IL17A and IFN-gamma co-expression in CD154^+^TNF-α^+^ memory CD4^+^ T cells after short-term stimulation with heat-inactivated bacteria (n = 23−34 independent donors). (*C*) Lamina propria mononuclear cells (LPMCs) from inflamed IBD surgical specimens or non-inflamed and tumor-free surgical specimens from colorectal cancer patients were stimulated with heat-inactivated *E coli.* Boolean gating shows each possible combination of IL17A, IFN-gamma, and IL22 production by CD154^+^TNF-α^+^ memory CD4^+^ T cells (n = 6 and n = 7 independent donors for IBD and controls, respectively). (*D*) Quantitative polymerase chain reaction analysis of *IL1B, IL6*, and *IL23A* in intestinal mucosal specimens categorized by endoscopic assessment of disease activity. Demographic and clinical characteristics of IBD patients are summarized in Supplementary Table 5. (*E*) CD4^+^CD45RO^+^CD45RA^−^CD25^−^CD8^−^ memory CD4^+^ T cells were isolated from healthy donor blood, labeled with CFSE, and stimulated with autologous monocytes pulsed with *B animalis* in the presence or absence of the indicated cytokines. Data represent mean (±SEM) fold-changes in IL17A or IFN-gamma expression frequencies relative to cells expanded without cytokines. Statistics: (*A, B*, *C*) Mann-Whitney test; (*D*, *E*) 1-way analysis of variance with Sidak’s multiple comparison test.

To evaluate functional alterations in microbiota-reactive CD4^+^ T cells in IBD, intracellular CD154 detection was combined with cytokine analysis. Compared with healthy controls, circulating microbiota-reactive CD4^+^ T cells from IBD patients displayed increased IL17A and IL2 production, but decreased expression of IFN-gamma (Figure 7*A* and Supplementary Figure 6*D* and *E*). Interestingly, increased IL17A production was observed in all enteric bacteria-reactive responses, but not in *S aureus, M tuberculosis*, or SEB responses (Figure 7*A* and Supplementary Figure 6*E* and *F*). These changes were observed in both Crohn’s disease and ulcerative colitis and were independent of disease activity or therapy (Supplementary Figure 6*G*). However, no difference in IL10 production was observed between healthy donors and IBD patients (Supplementary Figure 6*H*). IFN-gamma and IL17A co-expression is thought to identify pathogenic CD4^+^ T cells in mouse colitis models,^36^ so we assessed their co-expression in *E coli*−reactive memory CD4^+^ T cells. Compared with controls, IBD patients displayed significantly increased frequencies of IL-17A^+^IFN-gamma^−^ cells and a marginal increase in IL17A^+^IFN-gamma^+^ cells, while the IL17A^−^IFN-gamma^+^ fraction was reduced significantly (Figure 7*B*). *E coli*−reactive CD4^+^ T cells from inflamed intestinal tissue showed an increase in IL17A single producers similar to that seen in peripheral blood (Figure 7*C*).

Because the Th17-inducing cytokines *IL1B*, *IL6*, and *IL23A* were highly enriched in the inflamed intestinal tissue of IBD patients (Figure 7*D*), we reasoned that they might promote Th17 polarization of bacteria-reactive T cells. Indeed, treatment of microbiota-reactive CD4^+^ T cells from healthy donors and IBD patients with IL1β, IL6, or IL23 for 1 week during stimulation with *E coli, S typhimurium, L acidophilus*, or *B animalis* (CFSE dilution assay) resulted in a 1.5- to 2-fold increase in IL17A production (Figure 7*E* and Supplementary Figure 6*I*).

Taken together, these experiments demonstrate that circulating and gut-resident microbiota-reactive CD4^+^ T cells express increased frequencies of IL17A in IBD. Intestinal tissues from patients with active IBD express gene modules driven by Th1/Th17-derived cytokines, suggesting that bacteria-reactive memory cells could contribute to the tissue response.

## Discussion

The gastrointestinal tract harbors a large and diverse population of commensal bacteria, and how the immune system interacts with them is subject to intense investigation. Here we used 2 different methodologies to characterize microbiota-reactive CD4^+^ T-cell frequencies and phenotypes in the blood and intestinal tissue of healthy individuals and those with IBD. For each bacterial strain tested, the healthy CD4^+^ T-cell repertoire contains reactive cells at a frequency of 40−4000 per million, consistent with other antigen-reactive memory T cells.^37^ Microbiota-reactive CD4^+^ T cells were mainly of a memory phenotype, present in both blood and gut tissue, had a diverse TCR Vβ repertoire, and showed little clonotype sharing. Notably, microbiota-reactive CD4^+^ T cells were functionally heterogeneous in terms of homing receptor expression and effector functions and could stimulate intestinal cells via production of IL17*A*, IFN-gamma, IL22, and TNF-α. In addition, microbiota-reactive CD4^+^ T cells were recruited to sites of inflammation and showed increased IL17A production in patients with IBD.

Characterizing T-cell responses to bacteria is technically challenging due to their complex antigenic makeup. We therefore used the CD154 and CFSE dilution assays, both of which exploit microbial complexity, to provide large numbers of antigens. The combination of CFSE dilution and TCR Vβ sequencing allowed us to quantify clonotype heterogeneity and sharing between different bacteria-reactive T cells. Given the phylogenetic similarity of several bacteria used in this study, the paucity of clonotype sharing was surprising. Nevertheless, enteric bacteria-reactive T cells could be cross-reactive to other antigens not assessed in this study, and may have been primed during immune responses to other targets.^38^ High interclonal and intraclonal functional heterogeneity in human CD4^+^ T-cell responses to microbes and vaccines was observed recently.^19^ However, clonotype sharing between different microbial stimuli has not been studied previously and requires further investigation.

Microbiota-reactive CD4^+^ T cells showed substantial phenotypic and functional heterogeneity. The majority of circulating enteric bacteria-reactive CD4^+^ T cells co-expressed chemokine receptors, including CCR4, CCR6, and CCR7, while a smaller fraction expressed the gut-related homing receptors α4β7 and CCR9. These receptors promote access to secondary lymphoid organs and various mucosal tissues, including the intestine.^4^, ^27^ In addition, circulating and gut-resident microbiota-reactive T cells displayed both Th17 and Th1 characteristics and, in some cases, produced IL10.^39^ Gut-resident cells showed a clear Th17 bias when compared to circulating populations, which was more pronounced in IBD.

**Supplementary Figure 7:**
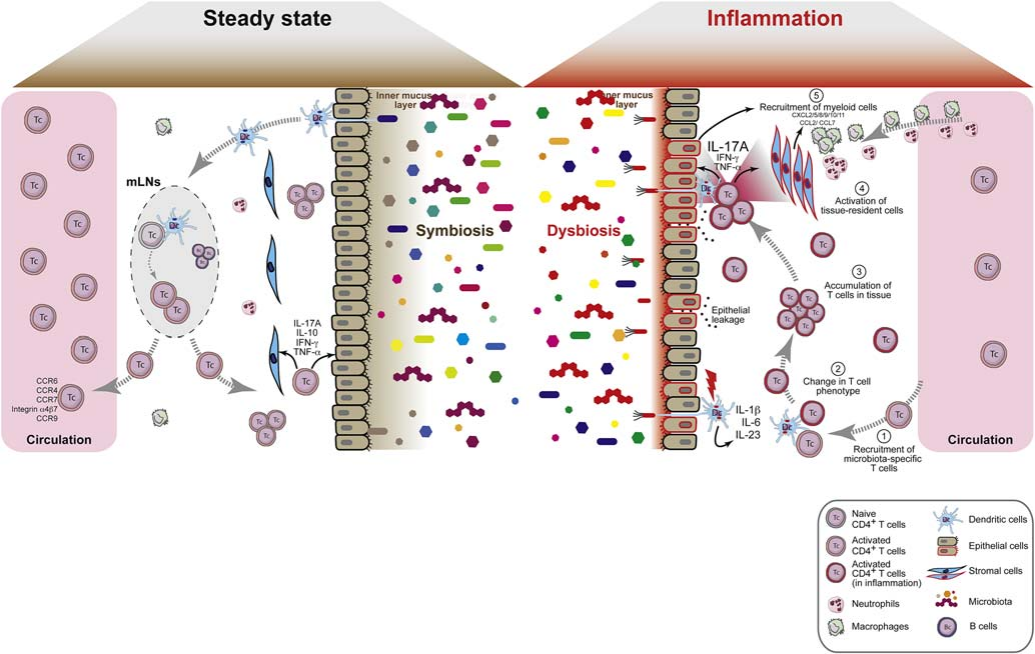
Microbiota-specific memory T-cell responses in intestinal homeostasis and inflammation. Microbiota-specific memory CD4^+^ T cells are abundant in peripheral blood and mucosa. Luminal sampling of intestinal microbiota by dendritic cells and repetitive epithelial breaches after birth might induce CD4^+^ T-cell responses targeted towards intestinal bacteria. Enteric bacteria-specific CD4^+^ T cells produce barrier-protective cytokines, such as IL17A, IFN-gamma, and IL22. Furthermore, certain commensal-specific CD4^+^ T cells also produce IL10. We speculate that the cytokine production by commensal-specific CD4^+^ T cells might play a significant role in supporting gut homeostasis and the mutualistic relationship with intestinal microbiota. In inflammation, epithelial break down and leakage leads to increased availability of luminal antigens and bacterial stimuli, which activate the innate system and the expression of Th17-inducing cytokines (IL1β, IL6, and IL23). Recruited microbiota-specific memory CD4^+^ T cells accumulate in inflamed mucosa and are polarized toward more IL17A production. Activated memory cells express IL17A, TNF-α, and IFN-gamma, which stimulate intestinal cells, such as stromal and epithelial cells, to express various chemokine ligands and cytokines. This may promote recruitment and activation of myeloid cell populations.

Based on our observations, we can speculate that continuous sampling of luminal antigens by intestinal dendritic cells causes low-level stimulation of gut-resident CD4^+^ T cells to produce cytokines that support epithelial integrity, barrier function, and intestinal homeostasis.^40^, ^41^ Indeed, cytokine production by commensal-reactive CD4^+^ T cells might play a more significant role in supporting gut homeostasis than previously thought (Supplementary Figure 7). However, this homeostatic circuit might be disrupted in IBD due to dysbiotic changes and/or perturbed myeloid cell activity, causing inappropriate T-cell activation and a pathogenic imbalance of cytokine production.^8^

While IL17A is frequently cited as a pathogenic cytokine, it is also critical for promoting mucosal barrier function and protection from pathogens.^31^ Absence of IL17A was recently shown to increase epithelial injury and compromise barrier function in mouse models of colitis.^42^, ^43^ Indeed, IL17A is a critical driver of neutrophil recruitment, and its absence could therefore exacerbate mucosal inflammation by facilitating bacterial invasion and dispersal.^44^ Notably, blockade of IL17A in Crohn’s disease caused disease exacerbation, despite being well tolerated and therapeutically effective in psoriasis.^45^ Thus, IL17A likely plays a key tissue-protective role in humans, suggesting that the increased Th17 polarization of microbiota-reactive T cells in IBD patients could reflect an effort to bolster tissue integrity.

Host-microbial homeostasis depends on minimizing contact between micro-organisms and mucosal surfaces via the combined action of epithelial cells, mucus, IgA, antimicrobial peptides, and immune cells.^1^, ^2^ Active immune responses to gut flora have been linked to disease.^8^ However, this concept should be revisited in light of our current findings and the observation that healthy individuals generate antibody responses to commensals.^46^, ^47^ At least 2 plausible mechanisms could explain the genesis of these microbiota-reactive responses. First, mucosal dendritic cells constantly survey the luminal microenvironment and thereafter migrate to secondary lymphoid tissues to initiate B- and T-cell responses.^48^, ^49^ Second, during gastrointestinal infections in mice, immune responses against commensals and pathogens are induced in parallel.^50^ Continuous luminal sampling of intestinal microbiota and periodic epithelial breaches during gastrointestinal infections might provide a plethora of memory T cells with potential reactivity toward newly encountered pathogens.^6^, ^7^ Therefore, contrary to the notion that they promote inflammatory pathology, acquired commensal-reactive T-cell responses may be essential to promote barrier function and IL10-mediated immune regulation—2 cornerstones of intestinal homeostasis*.*

## Supplementary Experimental Procedures

### Human Samples and Cell Isolation

PBMCs were isolated by Ficoll-Paque (GE Healthcare Life Sciences, Little Chalfont, UK) density gradient centrifugation, resuspended in phosphate-buffered saline with 2 mM EDTA and 0.02% bovine serum albumin, and further processed. Gut specimens were obtained from patients with IBD undergoing surgery for severe, chronically active, or complicated disease. Control gut specimens from macroscopically healthy areas were collected from colorectal cancer patients as non-inflammatory controls. One intestinal pinch biopsy was obtained from healthy donors (colorectal cancer screening, or other non-IBD related conditions) or IBD patients during routine endoscopy, from lesional, and non-lesional sites, attending the John Radcliffe Hospital (Oxford, UK). Inflammation status of biopsies was binarized into either inflamed or uninflamed categories based on endoscopic assessment. Additional information about the analyzed IBD patients can be found in Supplementary Tables 5−7.

Lamina propria mononuclear cells were isolated as described previously.^1^ In brief, mucosa was dissected and washed in 1 mM dithiothreitol at room temperature for 15 minutes to remove mucus. Specimens were washed 3 times with 0.75 mM EDTA at 37°C for 45 minutes to detach epithelial crypts and digested overnight with 0.1 mg/mL collagenase D (Roche, Indianapolis, IN). Cells were centrifuged for 30 minutes in a Percoll gradient and collected at the 40%−60% interface. All solutions were supplemented with antibiotics (penicillin/streptomycin, 40 μg/mL gentamicin, and 0.025 μg/mL amphotericin B).

### Flow Cytometry

Cells were stained with the following monoclonal antibodies as described^2^: CD4-BV510 (OKT4), CD4 phycoerythrin (PE)-Dazzle (RPA-T4), CD3-PE-CF594 or –BV510 (UCHT1), CD45RA-BV711 (HI100), CD45RO-BV570 (UCHL1), CD161-eF450 (HP3G10), CCR7-PE (G034H7), CCR2-APC (K036C2), CCR6-BV605 (G034E3), CCR9-PE-Cy7 (L053E8), CXCR3-PE/Cy5 (1C6/CXCR3), CCR4-PE-Cy7 (1G1), CCR10-PE (314305), Integrin β7-FITC/APC (FIB504), CD69-PE-Cy7 (FN50), IL-2-BV650 (MQ1-17H12), TNF-α-PB (MAb11), CD154-FITC/PE-Cy5 (24-31), IL-17A BV711 (eBio64DEC17), IL-22-PE-Cy7 (22URTI), IFN-γ-AF700 (B27), GM-CSF-APC (BVD2-21C11), and IL-4-PE (8D4-8) (eBioscience, Biolegend, San Diego, CA and Becton Dickinson, San Jose, CA). Fluorescein-activated cell sorting panels contained up to 12 fluorochromes. Compensation beads were used for compensations (BD Bioscience, San Jose, CA). TCR Vβ usage was assessed using the IOTest Beta Mark TCR Vbeta Repertoire Kit (Beckman Coulter, Brea, CA). Samples were acquired on FACS LSRFortessa and FACSLSRII (Becton Dickinson); ≥2 × 10^5^ memory CD4^+^ T cells were acquired. Data were analyzed with FlowJo (Tree Star, Ashland, OR) and SPICE. For analysis of cytokine expression by microbiota-specific T cells, a minimum of 20 CD4^+^ CD154^+^ TNF-α^+^ T cells was used; donors with lower events were excluded.

### CD154 Enrichment of Antigen-Specific T Cells

For magnetic cell separation enrichment of CD4^+^CD154^+^ T cells, CD154 Enrichment and Detection Kit was used (Miltenyi Biotec). Briefly, cells were plated at 5 × 10^6^/cm^2^ for 7−12 hours with heat-inactivated bacteria in the presence of anti-CD40 blocking antibody (HB14) and anti-CD28 stimulation antibody (CD28.6). Anti-CD40 blockade prevents down-regulation of CD154, while CD28 co-stimulation optimizes induction of CD154 expression. For major histocompatibility complex II blockade, 10 μg/mL of a pan-HLA class-II blocking antibody (HLA-DR, DP, DQ; [Tü39]) was added 30 minutes before bacterial stimulation. Cord and adult blood analysis was performed identically. Antigen-presenting cell abundance is similar in cord and adult blood.^3^, ^4^

### Cross-Reactivity Assay

CD154^+^ CD4^+^ T cells were isolated using magnetic cell separating enrichment and fluorescein activated cell sorting from total PBMCs after stimulation with *E coli Nissle* and *B animalis* lysates as described. CD154^+^ CD4^+^ T cells were expanded for 10−14 days with IL2 and anti-CD3/CD28 beads (beads/T cell ratio, 1:4, Dynals). The expanded T-cell lines were washed in IL2 free medium and incubated for 12 hours without IL2 in RPMI-1640 supplemented with 2 mM glutamine, 1% (v/v) non-essential amino acids, 1% (v/v) sodium pyruvate, penicillin (50 U/mL), streptomycin (50 mg/mL; all from Invitrogen), and 5% (v/v) human serum (National Blood Service, Bristol, UK). The expanded T-cell lines were labeled with CFSE, and then were co-incubated with autologous monocytes loaded with various bacterial lysates. T cells were co-cultured with the irradiated autologous monocytes at a ratio of 2:1 for 5−7 days. The CFSE dilution was measured at the end of the culture. Autologous CD14^+^ monocytes were isolated from PBMC using anti-CD14 microbeads (Miltenyi Biotec) and frozen until the T-cell co-culture. Monocytes were thawed down, irradiated (45 Gy), and then pre-incubated for 3 hours with bacterial lysates before T-cell co-culture.

### Intracellular Cytokine Analysis

For intracellular cytokine staining, cells were restimulated with phorbol myristate acetate (5 ng/mL) and ionomycin (500 ng/mL; Sigma) or bacterial lysates. Brefeldin A (5 μg/mL; Sigma-Aldrich) was added at 2 hours. After 4−12 hours, cells were stained with fixable viability dye eFluor 780 (eBioscience) and surface markers, fixed with 2% formaldehyde (Merck), and stained for cytokines in buffer containing 0.05% saponin (Sigma-Aldrich). In some assays, cytokine analysis was combined with intracellular CD154 detection, as described previously.^5^, ^6^

### Transcription Factors Staining

Transcription factor expression was analyzed using the FoxP3 staining buffer set (eBioscience) according to manufacturer’s instructions. Briefly, cells were stained with fixable viability dye eFluor 780 (eBioscience) and surface markers, fixed with 1× fixation/permeabilization buffer, followed by antibody staining and washing in 1× permeabilization buffer. T-box expressed in T cells−Pacific Blue (4B10) and GATA-binding factor-3−Alexa-647 (TWAJ) were from eBioscience. Related orphan receptor γt-PE (Q21-559) was from BD Bioscience.

### Preparation of Bacterial Lysates

Different bacteria were cultured in their respective optimal media for 16 hours at 37°C, washed in sterile phosphate-buffered saline and heat-inactivated at 65°C for 1 hour, followed by 3 freeze−thaw cycles. Extremely oxygen−sensitive bacteria were provided by Sylvia Duncan, University of Aberdeen, after heat inactivation. Suspensions were centrifuged at maximum speed for 15 minutes and supernatants collected. Protein concentration was quantified using Nanodrop (Thermo Fisher Scientific, Waltham, MA). The following bacterial strains were used: *B vulgatus* (Bv 1447), *B animalis* subsp *lactis* Bi-07, *C difficile* (OXF1003, Toxin AB^-^), *E coli* (Nissle 1917*), F prauznitzii* (A2-165), *L acidophilus* (NCFM), *R intestinalis* (M50/1), *R obeum* (A2-162), *Salmonella enterica serovar typhimurium* (NCTC 12023), and *S aureus* (NCTC 6571). Additional information regarding the bacterial stains is provided in Supplementary Table 1. Bacterial lysates were titrated in CFSE dilution assay and an optimal concentration was used (5−15 μg/mL). Tetanus toxoid (Calbiochem, San Diego, CA) and influenza seasonal vaccine (OPTAFLU; Novartis, Basel, Switzerland) were used at 5 μg/mL. Heat-killed *M tuberculosis* (H37Ra) and *C albicans* were from InvivoGen (San Diego, CA). Ultrapure lipopolysaccharide-EB from *E coli* 0111:B4 (InvivoGen) was used as a stimulation control. SEB was used at 1 μEBmL (Sigma).

### RNA Extraction, Complementary DNA Synthesis, and Quantitative Polymerase Chain Reaction

Tissue was homogenized using a motor with sterile RNase/DNase-free disposable pestles (both VWR) in RLT buffer (Qiagen, Valencia, CA). Cells were lysed directly in RLT buffer. RNA was isolated using RNeasy Mini kit (Qiagen) or Quick-RNA MiniPrep kit (Zymo Research, Irvine, CA) followed by complementary DNA preparation using High-Capacity cDNA Reverse Transcription Kit (Applied Biosystems, Carlsbad, CA) with random hexamers. Quantitative polymerase chain reaction (PCR) was performed using a CFX96 (Bio-Rad, Hercules, CA) or ViiA7 384-well real-time PCR system (Applied Biosystems) with TaqMan assays (Life Technologies, Carlsbad, CA), and PrecisionPLUS Mastermix (Primerdesign, Southampton, UK). Expression levels were normalized to a housekeeping (hk) gene (*RPLP0*) and expressed as 2ˆ-(CTgene-CThk). Heat maps were made using Cluster 3.0 and Java TreeView (Michael Eisen, Stanford University).

### CIBERSORT Analysis

To calculate enrichment of cell populations using CIBERSORT,^7^ we analyzed the GSE16879 dataset on default settings. For each sample, relative expression of *PTPRC* (CD45, representing relative leukocyte content) was calculated separately using median-normalized microarray data. This value was then multiplied by CIBERSORT cell type scores (eg, proportion of memory CD4^+^ T cells in the total leukocyte fraction) to estimate cell type enrichment levels. Finally, for cell types of interest, fold differences between IBD and control specimens were calculated to estimate relative cell type abundance in active IBD lesions versus healthy tissue.

### T-Cell Receptor Vβ Sequencing and Analysis

Memory T cells (3.6 × 10^6^) were cultured with autologous monocytes pulsed with heat-inactivated bacteria from 3 healthy donors. Expanded CFSE^low^ ICOS^+^CD4^+^ T cells were sorted into DNase/RNase free water with bovine serum albumin (10 mg/mL) in 96-well plates (100 cells/well) and stored at −80°C. TCR Vβ sequence analysis was obtained by a series of 3 nested PCR reactions, as described previously.^8^ Reverse transcription and preamplification were performed with a One-Step RT-PCR kit (Qiagen) using multiplex PCR with multiple Vβ region primers and a Cβ region primer. After the first reaction, an aliquot was used for the second PCR using a set of multiple internally nested TCR Vβ primers and internally nested Cβ region primer with HotStarTaq DNA polymerase kit (Qiagen). In the final PCR reaction, an aliquot of the second PCR was used and amplification was performed using barcoding primers containing the common 23-base sequence (incorporated into the second set of Vβ primers) and a third internally nested Cβ primer and Illumina Pair-End primers. After the third PCR reaction, each PCR product should have a unique set of barcodes incorporated that specifies plate, row, and column and have Illumina Paired-End sequences that enabled sequencing on the Illumina MiSeq platform. The PCR products were combined at equal proportion by volume, run on a 1.2% agarose gel, and a band around 350 to 380 bp was excised and gel purified using a Qiaquick gel extraction kit (Qiagen). Barcoded products were sequenced on the Illumina MiSeq platform.

Sequence reads were trimmed using ‘Trim Galore' software (http://www.bioinformatics.babraham.ac.uk/projects/trim_galore/) with default settings. Reads were de-multiplexed using a custom Python script according to the presence of index sequences. A further custom script then split reads from each well according to the presence of primer sequences used in the PCR amplification. These reads were then analyzed by MICXR, version 1.6 (http://www.nature.com/nmeth/journal/v12/n5/full/nmeth.3364.html) using settings appropriate for the anticipated TCR locus of origin (A or B). MIXCR provided CDR3 sequences along with counts of the number of times each sequence was observed. Detected CDR3 sequences were filtered to exclude those with ≤10 counts per well. There were 48 wells per donor of SEB-stimulated or phytohemagglutinin-stimulated cells while there were 96 wells of bacterially stimulated cells. Furthermore, some wells did not contain any valid TCR sequences after filtering for low read counts, suggesting that amplification or sequencing had failed for those wells. To permit comparisons of the number of TCR sequences between plates with differing numbers of successful wells, counts were scaled to the theoretical maximum of 96 wells.

## Supplementary Material

Supplementary Table 1 Enteric Bacterial Species and Control Species Used in the Study (Related to Figure 1)

Supplementary Table 2 T-Cell Receptor Vβ Usage in Expanded Microbiota-Reactive T Cells Measured by Vβ Antibody Panel from Beckman Coulter (Related to Figure 3)

Supplementary Table 3 T-Cell Receptor Vβ Usage in Expanded Microbiota-Reactive T cells Using Multiplex Polymerase Chain Reaction Assay and Deep Sequencing (Related to Figure 3)

Supplementary Table 4 CDR3 Sequences of Microbiota-Reactive Memory CD4^+^ T Cells (Related to Figure 3)

Supplementary Table 5 Clinical Characteristics of Oxford Cohort Patients Assessed in This Study for Gene Expression Analysis (Related to Figures 5*E* and 7*D*)

Supplementary Table 6 Clinical Characteristics of Oxford Cohort Patients Assessed in This Study for the Analysis of Microbiota-Specific CD4 T Cells (Related to Figure 7*A*, *B*, and Supplementary Figures 6*A*−*H*)

Supplementary Table 7 Clinical Characteristics of Oxford Cohort Patients Assessed in This Study for Cell Accumulation in the Mucosa (Related to Figure 3*B*, *C* and Figure 7*C*).
