# Supplementary material for: Circulating and Tissue-Resident CD4+ T Cells With Reactivity to Intestinal Microbiota Are Abundant in Healthy Individuals and Function Is Altered During Inflammation
Source: Gastroenterology. 2017 Nov;153(5):1320–1337.e16. doi: 10.1053/j.gastro.2017.07.047 (PMC5687320; doi:10.1053/j.gastro.2017.07.047)
Supplement: Supplementary Table 1 — Enteric Bacterial Species and Control Species Used in the Study (Related to Figure 1) [file mmc1.pdf]

Supplementary Table 1. Enteric bacterial species and control species used in the study

Bacterial species	Strain Designation	Family	Phylum	Kingdom	Phylum abundance	Gram stain	Growth characteristics (of the species)	Changes during IBD on Phylum level	Reference
Bacteroides vulgatus	Bv 1447	Bacteroidaceae	Bacteroidetes	Bacteria	~20-40%	Negative	Obligate anaerobe	↓	1, 2, 3, 4
Bifidobacterium animalis subsp. lactis	Bi-07	Bifidobacteriaceae	Actinobacteria	Bacteria	~1-10%	Positive	Obligate anaerobe	↓ (at family level)	1, 2, 3, 4
Clostridium difficile	OXF1003, Toxin AB-	Clostridiaceae	Firmicutes	Bacteria	~50-70%	Positive	Obligate anaerobe	↓	1, 2, 3, 4
Escherichia coli	Nissle 1917	Enterobacteriaceae	Proteobacteria	Bacteria	~5-15%	Negative	Facultative anaerobe	↑	1, 2, 3, 4
Faecalibacterium prauznitzii	A2-165)	Ruminococcaceae	Firmicutes	Bacteria	~50-70%	Positive	Obligate anaerobe	↓	1, 2, 3, 4
Lactobacillus acidophilus	NCFM	Lactobacillaceae	Firmicutes	Bacteria	~50-70%	Positive	Obligate anaerobe	↓	1, 2, 3, 4
Roseburia intestinalis	M50/1	Lachnospiraceae	Firmicutes	Bacteria	~50-70%	Positive	Obligate anaerobe	↓	1, 2, 3, 4
Ruminococcus obeum	A2-162	Lachnospiraceae	Firmicutes	Bacteria	~50-70%	Positive	Obligate anaerobe	↓	1, 2, 3, 4
Salmonella enterica serovar typhimurium	NCTC 12023	Enterobacteriaceae	Proteobacteria	Bacteria	~5-15%	Negative	Facultative anaerobe	↑	1, 2, 3, 4
Staphylococcus aureus	NCTC 6571	Staphylococcaceae	Firmicutes	Bacteria	~50-70%	Positive	Facultative anaerobe	not invovled (at family level)	1, 2, 3, 4
Mycobacterium tuberculosis	H37Ra	Mycobacteriaceae	Actinobacteria	Bacteria	~1-10%	Positive	Obligate aerobe	not invovled (at family level)	1, 2, 3, 4
Candida albicans		Debaryomycetaceae	Saccharomycetes (Class)	Fungi			Facultative anaerobe	↑	1, 2, 3, 4

References

1. Morgan XC, Tickle TL, Sokol H, et al. Dysfunction of the intestinal microbiome in inflammatory bowel disease and treatment. Genome Biol. 2012;13:R79.
2. Kostic AD, Xavier RJ, Gevers D. The microbiome in inflammatory bowel disease: current status and the future ahead. Gastroenterology 2014;146:1489–1499.
3. Sokol H, Leducq V, Aschard H, et al. Fungal microbiota dysbiosis in IBD. Gut 2016.
4. Gevers D, Kugathasan S, Denson LA, et al. The Treatment-Naive Microbiome in New-Onset Crohn's Disease. Cell Host Microbe 2014;15:382–392.
