# Supplementary material for: Circulating and Tissue-Resident CD4+ T Cells With Reactivity to Intestinal Microbiota Are Abundant in Healthy Individuals and Function Is Altered During Inflammation
Source: Gastroenterology. 2017 Nov;153(5):1320–1337.e16. doi: 10.1053/j.gastro.2017.07.047 (PMC5687320; doi:10.1053/j.gastro.2017.07.047)
Supplement: Supplementary Table 2 — T-Cell Receptor Vβ Usage in Expanded Microbiota-Reactive T Cells Measured by Vβ Antibody Panel from Beckman Coulter (Related to Figure 3) [file mmc2.pdf]

Supplementary Table 2. T cell receptor (TCR) Vβ usage in expanded microbiota-specific T cells measured by Vβ antibody panel from Beckman Coulter

(related to Figure 3)

Donors	Microbe	Vβ1	Vβ2	Vβ3	Vβ4	Vβ5.1	Vβ5.2	Vβ5.3	Vβ7.1	Vβ7.2	Vβ8	Vβ9	Vβ11
		TRBV9	TRBV21-1	TRBV28	TRBV29-1	TRBV5-1	TRBV5-6	TRBV5-5	TRBV4-1,2,3	TRBV4-3	TRBV12-3,4	TRBV3-1	TRBV25-1
**Donor A**	*S. typhimurium*	1.98	8.34	3.47	1.61	4.97	0.463	0.451	5.69	0.485	7.15	3.55	0.482
**Donor A**	*E. coli*	5.32	9.6	3.8	1.2	7.78	1.32	0.393	0.892	1.25	4.25	1.86	0.968
**Donor A**	*B. animalis*	1.86	11.1	3.87	0.504	8.59	0.301	0.705	0.888	0.135	1.9	3.03	0.272
**Donor A**	*L. acidophilus*	2.06	11.7	4.68	0.744	7.18	0.513	0.239	0.647	0.216	2.4	1.94	0.275
**Donor A**	*F. prausnitzii*	1.39	8.16	2.38	0.682	7.13	0.321	0.368	1.12	0.581	2.2	2.82	0.374
**Donor A**	*C. difficile*	0.821	9.46	4.61	1.52	9.56	0.219	0.592	1.43	0	1.54	3.27	0
**Donor A**	SEB	0.0745	0.0962	25.9	0.418	0.00517	0.446	0.0707	0.0943	0.07	0.165	0.027	0.0528
**Donor A**	PHA	2.22	13.5	5.99	2.08	1.07	6.21	0.838	0.932	0.673	2.5	3.11	0.44
**Donor B**	*S. typhimurium*	3.39	9.76	5.28	2.27	7.64	0.672	0.721	0.569	0.696	5.5	4.63	0.331
**Donor B**	*E. coli*	3.52	11.9	4.32	1.96	7.97	0.714	0.921	1.02	1.56	5	4.22	0.184
**Donor B**	*B. animalis*	2.36	4.07	7.63	4.29	7.72	0.461	3.41	1.12	1.48	3.23	1.81	0.575
**Donor B**	*L. acidophilus*	8.58	11	3.49	4.26	9.74	0.762	1.81	0.499	2.15	4.19	0.955	0.416
**Donor B**	*F. prausnitzii*	3.06	5.41	6.07	2.15	7.49	0.535	0.748	0.748	1.77	3.85	1.03	0.416
**Donor B**	*C. difficile*	1.95	9.02	2.8	1.46	4.5	0.185	0.781	0.588	0.308	1.44	1.43	0.238
**Donor B**	SEB	0.226	0.175	29.4	0.939	0.0283	0.434	0.107	0.0555	0.0598	0.274	0.077	0.0443
**Donor B**	PHA	0.808	10.1	4.83	0.908	0.698	3.32	0.531	0.363	0.147	1.93	2.64	0.227
**Donor C**	*S. typhimurium*	1.52	1.21	4.72	32.9	0.998	0.31	0.0963	0.84	0	9.8	0.357	0.0977
**Donor C**	*E. coli*	1.34	1.22	4.53	26.3	1.28	0.181	0.167	0.642	0	9.4	0.337	0.136
**Donor C**	*B. animalis*	1.36	1.61	1.58	1.8	1.92	0.378	0.188	1.2	0.0113	1.08	0.286	0.21
**Donor C**	*L. acidophilus*	0.816	5.71	1.26	3.29	1.32	0.333	1.18	1.22	0.00834	2.58	0.943	0.261
**Donor C**	*F. prausnitzii*	0.498	1.51	0.6	1.73	0.585	0.234	0.109	0.269	0.0247	1.07	0.271	0.382
**Donor C**	*C. difficile*	0.471	1.09	0.536	0.778	0.615	0.218	0.145	0.563	0.0046	4.63	0.165	0.208
**Donor C**	SEB	0.191	0.155	19.9	1.43	0.0354	0.482	0.114	0.0692	0.0188	0.234	0.0615	0.0577
**Donor C**	PHA	2.01	10.2	5.36	2.43	0.963	6.44	0.958	0.626	0.00905	4.24	2.61	0.732

Supplementary Table 2. T cell receptor (TCR) Vβ usage in expanded microbiota-specific T cells measured by Vβ antibody panel from Beckman Coulter

**Donors**	**Microbe**	**Vβ13.1**	**Vβ13.2**	**Vβ13.6**	**Vβ14**	**Vβ16**	**Vβ17**	**Vβ18**	**Vβ20**	**Vβ21.3**	**Vβ22**	**Vβ23**	**Unkown Vβ**
		TRBV6-5,6,9	TRBV6-2	TRBV6-6	TRBV27	TRBV14	TRBV19	TRBV18	TRBV30	TRBV11-2	TRBV2	TRBV13	
**Donor A**	*S. typhimurium*	1.75	1.24	1.45	2.72	1.78	4.92	0.398	0.862	1.09	2.28	0.079	42.265
**Donor A**	*E. coli*	2.07	1.73	2.09	3.2	0.731	5.7	0.515	4.25	0.901	5.48	0.267	30.583
**Donor A**	*B. animalis*	0.727	6.54	0.998	1.89	1.09	7.64	0.197	1.98	1.41	15.5	0.955	27.014
**Donor A**	*L. acidophilus*	1.07	1.78	0.726	0.627	0.746	14.5	1.29	0.79	1.09	13	0.212	30.575
**Donor A**	*F. prausnitzii*	1.09	0.556	1.35	1.27	0.896	3.21	0.921	1.39	1.91	19.2	0.463	39.885
**Donor A**	*C. difficile*	1.09	1.35	1.39	2.07	0.369	2.48	1.13	3.5	1.86	11.9	0.0548	38.0542
**Donor A**	SEB	0.11	5.22	0.333	2.02	0.333	14.6	0.0234	2.73	0.0444	0.354	0.177	38.56553
**Donor A**	PHA	3.16	2.82	1.9	2.95	1.14	4.6	1.55	2.42	2.85	2.26	0.37	32.347
**Donor B**	*S. typhimurium*	0.912	3.07	3.71	2.06	0.43	5.21	0.335	0.167	3.12	6.83	0.706	31.111
**Donor B**	*E. coli*	2.47	3.98	2.96	2.71	0.792	5.14	1.41	0.2	3.11	4.82	0.432	27.947
**Donor B**	*B. animalis*	1.49	4.37	4.08	1.37	0.421	6.43	0.812	0.559	0.677	4.04	0.118	36.347
**Donor B**	*L. acidophilus*	2.67	2.82	3.02	2.7	0.148	6.01	0.409	0.36	1.2	6.69	0.076	25.096
**Donor B**	*F. prausnitzii*	1.07	1.17	4.92	6.59	0.972	8.73	0.237	0.917	0.764	14.1	0.115	24.568
**Donor B**	*C. difficile*	2.93	0.638	8.27	1.82	0.2	3.29	0.548	0.298	2.81	7.49	1.94	31.766
**Donor B**	SEB	0.104	7.76	0.724	2.03	0.424	23	0.0317	0.052	0.0674	0.542	0.788	22.657
**Donor B**	PHA	1.94	2	4.05	1.8	0.716	3.37	1.5	0.0536	2.16	0.869	0.223	53.3964
**Donor C**	*S. typhimurium*	1.05	0.133	0.81	4.43	0.084	0.942	0.0269	0.228	0.796	4.51	0.0442	33.9669
**Donor C**	*E. coli*	0.785	0.176	0.878	2.55	0.0657	1.26	0.0797	0.347	0.791	4.57	0.0959	42.6127
**Donor C**	*B. animalis*	1.05	0.317	0.803	0.52	0.068	2.27	0.222	0.979	0.339	1.3	0.133	80.1597
**Donor C**	*L. acidophilus*	1.41	0.233	0.415	1.33	0.0705	3	0.193	2.37	0.898	1.06	0.139	69.27716
**Donor C**	*F. prausnitzii*	0.543	0.52	0.291	1.43	0.0855	3.92	0.0425	1.03	0.33	0.645	0.102	83.5563
**Donor C**	*C. difficile*	0.327	0.092	0.138	0.482	0.0694	1.61	0.109	0.735	0.161	0.411	0.106	86.078
**Donor C**	SEB	0.206	3.48	0.345	3.34	0.381	19.9	0.0309	4.82	0.0836	1.04	0.257	34.3879
**Donor C**	PHA	3.55	1.7	2.16	3.4	1.09	4.67	1.09	3.14	3.11	1.79	0.487	35.36495

Vβ12
TRBV10-3
0.525
3.85
0.904
1
0.333
1.73
8.07
2.07
0.88
0.74
1.13
0.949
2.57
13.3
10
1.42
0.13
0.256
0.216
0.683
0.222
0.258
8.98
1.87
