# Supplementary material for: Circulating and Tissue-Resident CD4+ T Cells With Reactivity to Intestinal Microbiota Are Abundant in Healthy Individuals and Function Is Altered During Inflammation
Source: Gastroenterology. 2017 Nov;153(5):1320–1337.e16. doi: 10.1053/j.gastro.2017.07.047 (PMC5687320; doi:10.1053/j.gastro.2017.07.047)
Supplement: Supplementary Table 4 — CDR3 Sequences of Microbiota-Reactive Memory CD4+ T Cells (Related to Figure 3) [file mmc4.pdf]

CDR3 DNA Seq	Clone count
TGTGCCAGCAGCTTTCGGACTAGCGGCGAGCAGTACTTC	2677
TGTGCCAGCAGCCCCCCCGCGGGGTCCGGGAGCTGTTTTTT	1392
TGTGCCTGGAGTGTTTGGGGGACCCGGAACACTGAAGCTTTCTTT	979
TGTGCCTGGCAGAAACAGGACAACTATGGCTACACCTTC	885
TGTGCCAGCAGCCAAGAGCCAGGGCACAATCAGCCCCAGCATTTT	840
TGTGCCTGGAGTGTGGACAGTGGCAATCAGCCCCAGCATTTT	769
TGTGCCTGGAGTGTACTGGCTGCAGGCCAAGAGACCCAGTACTTC	706
TGTGCCAGCAACAAAGCGGCAAACCAAGAGACCCAGTACTTC	694
TGTGCCTGGAGTGTACGTGACAGGGAGGTCATTGAAGCTTTCTTT	647
TGTGCCTGGAGCAAAACAGGGGGCAGTGAGCAGTTCTTC	635
TGCGCCAGCAGCCAGTTAGGGCAGTACACCGGGGAGCTGTTTTTT	558
TGTGCCAGCAGCTTAGGAACAGAACCATCCTACGAGCAGTACTTC	478
TGTGCCTGGAGTACCCGGACCTACCAGGGAGAATTC	442
TGTGCCAGCAGCTTAGCGAGCCTAGCGGGAGAGCAGTACTTC	440
TGTGCCAGCAGCCCCGGGACAGTCACAGATACGCAGTATTTT	412
TGTGCCTGGAGCGTCGGGCCCGGCCTGAACACTGAAGCTTTCTTT	408
TGTGCCTGGACAAGACTCGGAAACACCATATATTTT	406
TGTGCCTGGAGACCTAGGACCAGCGGGGAGCTGTTTTTT	404
TGTGCCTGGGCGACAGGGGAAAATCAGCCCCAGCATTTT	403
TGTGCCTGGAGTGTCCGCGGAGAGACCCAGTACTTC	391
TGTGCCAGCAGCTCAGGGGCTAGCCCTCAAGAGACCCAGTACTTC	372
TGTGCCTGGAGTGTCGTTCTAGCGGGATTAGAGACCCAGTACTTC	355
TGTGCCAGCAGTCCCTTACTGAACACTGAAGCTTTCTTT	343
TGTGCCAGCAGCTCCTCGAATGAAAAACTGTTTTTT	331
TGCGCCAGCAGCCAAATTGGGGAGACCCAGTACTTC	325
TGTGCCAGCAGCCGGCCAGCAACGAACACTGAAGCTTTCTTT	311
TGTGCCTGCCGTGGCGGACAGGGGAACTATGGCTACACCTTC	307
TGTGCCTGGATAGCGGCGGGGAGAGAGACCCAGTACTTC	306
TGTGCCTGGAGTGGGGTTTCCAACACTGAAGCTTTCTTT	300
TGTGCCAGCAGCCCTCGTCCCCGTGAGCAGTTCTTC	299
TGTGCCAGCAGCGAGGGGACAGGGAACTATGGCTACACCTTC	299
TGTGCCTGGAGGGTAGCGGGAGGCCTTCTAGAGCAGTACTTC	295
TGTGCCAGCAGCCCCCCGGACAGCGGCTTACCCAATCAGCCCCAGCATTTT	288
TGTGCCTGGAGTGTCCTACAAGAGACCCAGTACTTC	288
TGTGCCAGCAGCTTAAACAGGGGCTCCTACGAGCAGTACTTC	282
TGTGCCTGGAGTAGCGGGCTAGCGGGCGGGGAGCTGTTTTTT	275
TGCAGCGTTGAAAGGGGGTCCAATGAGCAGTTCTTC	267
TGTGCCTGGCGTGGGGACAGATACTACGAGCAGTACTTC	267
TGTGCCTGGAGTGTAGGCGGGGGAGGAGATACGCAGTATTTT	262
TGTGCCAGCAGCTCCCGACTAGCCTCCGAGCAGTACTTC	257
TGTGCCAGCGGGACTAGCGGAGGCACAGATACGCAGTATTTT	255
TGTGCCTGGAGACCTACCGGTGCGCGAGATACGCAGTATTTT	254
TGTGCCTGGATGGTGTCCTATGGCTACACCTTC	254

TGTGCCAGCAGCCAAGCGGAAAGCAATCAGCCCCAGCATTTT	254
TGTGCCAGCAGCGCCCGACTAGCGGAAGAGACCCAGTACTTC	248
TGTGCCTGGAGAGACTACCGGGACAGGGGGAACACCTTC	247
TGTGCCAGCAGCTTCGGGGTGTGGAATTCACCCCTCCACTTT	242
TGTGCCAGCAGCTTGGGGCTAGCGGAAGATACGCAGTATTTT	241
TGTGCCAGCAGCTTAGCGGGGGGACTAACCTACGAGCAGTACTTC	240
TGTGCCAGCAGCCTCCCAAGTCCGGACGAGCAGTACTTC	238
TGTGCCAGCAGCTTACTTCCAGGGATGAACACTGAAGCTTTCTTT	237
TGTGCCAGCAGCTTCAGGGGGCTCCGCACAGATACGCAGTATTTT	236
TGTGCCTGGAGAGCCCCCGGGACCTTCCACGAGCAGTACTTC	235
TGTGCCAGCAGCTTAGACGGGACAGGGGATACTGAAGCTTTCTTT	229
TGTGCCAGCAGTCTCTCGATCGACACCGGGGAGCTGTTTTTT	229
TGTGCCAGCAGCGCAGCCGGGTTTTCCTATGGCTACACCTTC	228
TGTGCCAGCAGCGTGGACTATCAAGAGACCCAGTACTTC	225
TGTGCCAGCAGTCTCCAGTCGGGAGCGAAAGCCGGGGAGCTGTTTTTT	224
TGTGCCAGCAGCTTACTTTCTGGGGCCAACGTCCTGACTTTC	222
TGCGCCAGCAGCCAAGTTGCAGGGACCAGCTCTGGGGCCAACGTCCTGACTTTC	218
TGCGCCAGCAGCCAAGATCGGGGGACACGGGATCAGCCCCAGCATTTT	217
TGTGCCAGCAGCTTACCATTCTCCGGCAGTGAGCAGTTCTTC	214
TGTGCCTGGAGTGTGCCCCCCTCCTACAATGAGCAGTTCTTC	209
TGTGCCAGCAGCTTAGTATTCGGGAGGGCCTACAATGAGCAGTTCTTC	208
TGTGCCAGCAGCATAGGGAGCGGCCCTCAGTTCTTC	208
TGTGCCAGCAGCTTTAGTGGCTCCTACAATGAGCAGTTCTTC	207
TGTGCCAGCAGCTCTCCAAATGGACCCGTGAACACTGAAGCTTTCTTT	206
TGTGCCTGGAGCATTCCAGGGGCGGGTCAGCCCCAGCATTTT	206
TGTGCCTGGAGTAGATCACAGGGGGCGAGTCAGCCCCAGCATTTT	204
TGTGCCAGCAGCTTAACGTTTCCGCCGGAGACCCAGTACTTC	204
TGCGCCAGCAGCCGAATAGGGGTGGGAGATACGCAGTATTTT	202
TGTGCCTGGAGCCCCTTGGCAGGTGGGAACACTGAAGCTTTCTTT	195
TGTGCCAGCAGCTCGGGATTTTCCTACGAGCAGTACTTC	190
TGCGCCAGCAGCCAAGATGGGCAGGGTCGGGGTGAGCAGTTCTTC	190
TGTGCCAGCAGCTTAGTGGGGTCCTCTCCCCAAGAGACCCAGTACTTC	189
TGTGCCAGCAGCGGGGACAGCCTCTATGGCTACACCTTC	189
TGTGCCAGCAGCTTGATAGCCCGACTAATCGATGGCTACACCTTC	187
TGCAGCGTTGAAGCGTCCCTCGGTGAGCAGTTCTTC	185
TGTGCCAGCAGCTTAGGCGGAGGGTTTAGCACAGATACGCAGTATTTT	184
TGTGCCAGCAGCTCCCCGACGGCAGGCATGAACACTGAAGCTTTCTTT	183
TGTGCCAGCAGCTTGACAGTACTCACTGAAGCTTTCTTT	182
TGCGCCAGCAGTACAGGCACGAACACCGGGGAGCTGTTTTTT	180
TGTGCCTGGAGTGGGGTGCGACGAAATGAGCAGTTCTTC	180
TGTGCCCCTCTTAGTCCAGGGAGCACTGAAGCTTTCTTT	178
TGTGCCAGCAGGTCACGAGAGGGTCATAGCAATCAGCCCCAGCATTTT	176
TGTGCCAGCAGCTTAGGTCCGCTTTGGAACACCGGGGAGCTGTTTTTT	175
TGTGCCACCAGCAGAGCACATACGAGCTATGGCTACACCTTC	175
TGTGCCAGCAGCTTAGGTTCGGGTTTGCCGTACTTC	174

**TGTGCCTGGAGTGTACTACAGGGTGGCTACACCTTC**	169
**TGTGCCACCAGCAGAGATCTCGGGTCGGGCGGAGAGACCCAGTACTTC**	168
**TGTGCCAGCAGCTTAGGGGGATACTCCTACAATGAGCAGTTCTTC**	168
**TGTGCCAGCAGCCTCCGGACAGGGGGCGGTTATGAAAAACTGTTTTTT**	167
**TGTGCCAGCAGCTTAGCTAGCCGCTCCTACGAGCAGTACTTC**	167
**TGTGCCAGCAGCTCCGATTTGAACACTGAAGCTTTCTTT**	167
**TGTGCCAGCAGCTTAGGTTCGGGAAATAATGAGCAGTTCTTC**	166
**TGCGCCAGCAGCCAAGATACGTCCAGGAGTGAAGCTTTCTTT**	165
**TGTGCCAGCAGCTTAGATAGTAACGAGCAGTACTTC**	164
**TGTGCCAGCAGCCCCGTGAGTTCCCGGGACCTATCCTACGAGCAGTACTTC**	163
**TGTGCCTGGAAGGGTTGGCAGGGCCTCTATGGCTACACCTTC**	163
**TGTGCCAGCAGTTTGTTCACAGATACGCAGTATTTT**	160
**TGTGCCGGGACAGGGAGTATGGGGACCTATGGCTACACCTTC**	160
**TGTGCCACCAGCAGAGATAGGGGAGTCACTGAAGCTTTCTTT**	159
**TGCGCCAGCGATACAGGGGACCCTTCTAACTATGGCTACACCTTC**	157
**TGTGCCAGCAGCAACCCGACAGGGGGCACAGATACGCAGTATTTT**	157
**TGTGCCAGCAGTTACTCGTGGACTAGCGGTTCTGGGGAGCTGTTTTTT**	156
**TGTGCCAGCAGCTCCACCGGGGGGCAGTCACCCCTCCACTTT**	156
**TGTGCCAGCAGCTTAGGGAACAGGCGGCAAGAGACCCAGTACTTC**	154
**TGTGCCAGCAGCCGGGACAGGGAGGGTGGCTACACCTTC**	152
**TGTGCCAGCCTTAGGGGACAGGGGAATCAGCCCCAGCATTTT**	152
**TGTGCCTGGAGTGTACTTTCTGGGGTCCAGCCCCAGCATTTT**	152
**TGCGCCAGCAGCTTGGACAGCGCTGAAGCTTTCTTT**	150
**TGTGCCAGCAGCTTAGGGCCCTTGGCGGGAGCCCCCTACAATGAGCAGTTCTTC**	150
**TGTGCCAGCAGCTTAGAGACTAGCTCTAGAGAGACCCAGTACTTC**	150
**TGTGCCAGCACCCGGGACAGGGTCGACGAGCAGTACTTC**	150
**TGTGCCTGGGCCCCATCAGGGGAGGGGGACAATCAGCCCCAGCATTTT**	149
**TGCGCCAGCAGCCAAGATAGGGGAGCTAACTATGGCTACACCTTC**	149
**TGTGCCAGCAGCCCGAGTACTAGCGTCGATGAGCAGTTCTTC**	148
**TGTGCCTGGAGTGTACTAGCAGGGGTTTATGGCTACACCTTC**	148
**TGTGCCTGGAGTGTAATTGGAGGGAACTATGGCTACACCTTC**	147
**TGTGCCAGCAGCTTAGACCGAGGGGTGCAGTACTTC**	143
**TGCGCCAGCAGCCCCGGGGCGGGTTTGGGCAACTATGGCTACACCTTC**	143
**TGTGCCAGCAGCTGGTCTGCAGCCACAGATACGCAGTATTTT**	140
**TGTGCCTGGAGTCAGGGATGGACTGAAGCTTTCTTT**	140
**TGTGCCAGCAGCTTAGCTACGACAGGGAGGGACATTCAGTACTTC**	139
**TGCGCCAGCAGCCAAGCCGGGACCCCAGATACGCAGTATTTT**	138
**TGTGCCAGCAGCTCGGGTGGACAGGGGATGGATACGCAGTATTTT**	137
**TGTGCCAGCAGCCCGTCCGGGACAGTGGGGGAGCAGTACTTC**	137
**TGTGCCAGCAGCTTAGCGTCCCAGGGGACGCGGAGCGATACGCAGTATTTT**	136
**TGTGCCAGCAGCTTGGGGTCGGTCGGGGAGACCCAGTACTTC**	135
**TGCGCCAGCAGCCATGTATTTCGGATCGGGTCGCGTGGCACTGGGGATACGCAGTATTTT**	135
**TGTGCCAGCAGCTTCGGTCAGGGGGGCACAGATACGCAGTATTTT**	135
**TGTGCCAGCAGCTTGGACAGGGGCACTGAAGCTTTCTTT**	134
**TGTGCCAGCAGCACTAGCTCCTACAATGAGCAGTTCTTC**	134

**TGTGCCAGCAGCTATGATGAAAAACTGTTTTTT**	133
**TGTGCCAGCACCTCGGGAGGGGGCTTGGATACGCAGTATTTT**	132
**TGTGCCAGCAGCTTCCCCGGGACCTCAGGGGGCTACACCTTC**	131
**TGCGCCAGCAGCTTGGGGGCCTACGAGCAGTACTTC**	131
**TGTGCCTGGAGTAGGCGTTCCCAGGAGACCCAGTACTTC**	131
**TGTGCCAGCAGCTCTAGACAGGGCGGTGACACTGAAGCTTTCTTT**	130
**TGTGCCTGGAGTGCCGAACGGGACAGCCATGGCTACACCTTC**	130
**TGTGCCTGGAGTGGTCAGGCGAACTCTGGGGCCAACGTCCTGACTTTC**	129
**TGCGCCAGCAGCCAGGGGACAGGGCGACGGCCTCCGTACCAAGAGACCCAGTACTTC**	129
**TGTGCCAGCAGCCCGCTCGCTGAAGCTTTCTTT**	128
**TGTGCCAGCAGCCTATTTACAGGGACCGGGGAGCTGTTTTTT**	128
**TGTGCCAGCAGCCAAGAACGGGATCAGCCCCAGCATTTT**	128
**TGTGCCAGCAGCCAAGATAGCAATCAGCCCCAGCATTTT**	126
**TGTGCCAGCAGCCAAACAGGGCCATCCGACTATGGCTACACCTTC**	124
**TGCGCCAGCAGCCGCAGACTTGCAACTAATGAAAAACTGTTTTTT**	122
**TGCAGTGCTAGACCGGTCGTTAATCAGCCCCAGCATTTT**	122
**TGTGCCAGCAGCCTGGACAGGGAGGAGACCCAGTACTTC**	122
**TGTGCCACCAGCAGAGGAGGGGACGGATATGGCTACACCTTC**	122
**TGCAGCGTTGAACTTCCCTCACAGGGAGGAGAGACCCAGTACTTC**	121
**TGTGCCTGGAGTGACCCCGGCGGGGGCTCCTACAATGAGCAGTTCTTC**	120
**TGCGCCAGCAGCCAAGATAGAGGAAATCAGCCCCAGCATTTT**	119
**TGCGCCAGCAGCCAAGACGTTCCGGGGAGCTCTGGAAACACCATATATTTT**	118
**TGTGCCTGGGGTGGACTCCCCGGTGCAGATACGCAGTATTTT**	117
**TGCAGCGCCTCGGCCGGGACTAGCGTGGCCTACGAGCAGTACTTC**	116
**TGTGCCAGCAGCTTAGTGGGTCCTGGATCAGGGCTGGGGGACGAGCAGTACTTC**	116
**TGTGCCAGCAGCGTAGCGGGGGGAGGTCAGCCCCAGCATTTT**	115
**TGTGCCTGGAGTGTACAGGGTGGCGGGGAGCTGTTTTTT**	114
**TGTGCCAGCAGCTTAGTCCATCTCCGCAATGAGCAGTTCTTC**	114
**TGTGCCAGCAGCCAGCTGGGGGCAGGCGAGCAGTACTTC**	114
**TGTGCCTGGAGTGGGCGAAATGAAAAACTGTTTTTT**	114
**TGTGCCAGCAGCTCCGGAAGGACAGGGGAGACCCAGTACTTC**	112
**TGTGCCAGCAGCTTAGGAGGACAGGGTGGGAACACTGAAGCTTTCTTT**	111
**TGCAGCGTTCGTCCTAGCGGGAGCCACGAGCAGTACTTC**	110
**TGTGCCAGCAGCGGAGGGGGGTCGAGGAGCAATCAGCCCCAGCATTTT**	109
**TGTGCCAGCAGCTTAGCGGGAAAGACCCAGTACTTC**	108
**TGTGCCAGCAGCCCAGACAGGGGTGGTAGCTATGGCTACACCTTC**	107
**TGTGCCAGCAGCTCCCCCGCCGGGCAGGAAGCTCTTTCTAACTATGGCTACACCTTC**	106
**TGTGCCAGCAGCTCAGACTTTTATCCCTACGAGCAGTACTTC**	106
**TGTGCCTGGACCGATCGGAGGGGGGGTCCCAACGGGCAGTTCTTC**	106
**TGCAGCGTTGAAGTCTACAGTTCGAATGAGCAGTTCTTC**	106
**TGTGCCTGGAGTGTTGGGTCAAACAGCAATCAGCCCCAGCATTTT**	105
**TGTGCCAGCAGACCCGGGACAGGTGGAGACACCATATATTTT**	104
**TGTGCCTGGAGTGTAGGGACTAAGGAGACCCAGTACTTC**	104
**TGTGCCTGGAGAGGCCGGGGGGGGCTGAACACTGAAGCTTTCTTT**	103
**TGTGCCAGCAGCCCCAACCAGGGAAGTGGGGCCAACGTCCTGACTTTC**	103

TGTGCCAGCAGCGCCGGGGGCGACTATGGCTACACCTTC	103
TGTGCCAGCAGCCAAGATTTAGGGGGGGGCATTCAGTACTTC	103
TGTGCCAGCAGCCCAAGGGGGGGACGGACTTTCAATCAGCCCCAGCATTTT	102
TGTGCCAGCAGTTACGACAGGCAGGGGGAACAGTACTTC	102
TGTGCCAGCAACCGGGACGGTAGCTATGGCTACACCTTC	101
TGTGCCAGCAGCGTGACCAGGGTACAGTCTAACTATGGCTACACCTTC	101
TGTGCCAGCAGCCCCTTGGTGGGGGATCAGCCCCAGCATTTT	100
TGTGCCAGCAGCCGATTAGCAGCCAAAAACATTCAGTACTTC	100
TGTGCCAGCAGCTTAGACGGGATTCGGGAGCAGTACTTC	100
TGTGCCAGCAGCTTCAACTCGGGACAGGGGTACGAGCAGTACTTC	99
TGTGCCTGGAAACCGGGACTAGCGTCGAACACCGGGGAGCTGTTTTTT	99
TGTGCCAGCAGCTTAGCCCCAGCGGGGGGTGGCACAGATACGCAGTATTTT	99
TGTGCCAGCAGGCGCCTCCAGGGGCCGAGCTATGGCTACACCTTC	99
TGTGCCTGGACTCTGTCTGGTGGAAACACCATATATTTT	98
TGTGCCAGCAGCTCCGAAACAGGGTCTGGAAACACCATATATTTT	98
TGTGCCAGCAGCTTAACGCGACAGAGCACAGATACGCAGTATTTT	98
TGTGCCAGCAGCTTAGATGGGACAGGGGACACTGAAGCTTTCTTT	97
TGCGCCAGCAGCCAAGACAAAGATACGCAGTATTTT	97
TGTGCCAGCAGCTTTTCGACAGATACGCAGTATTTT	97
TGTGCCAGCAGCTTAGACTTAAAGACTGGCTACACCTTC	96
TGTGCCAGCAGCCCCGGGACTAGCGGGGTAAATGAGCAGTTCTTC	95
TGTGCCAGCAGCCCAACTAGCGGGGGAGATACGCAGTATTTT	95
TGTGCCAGCAGCTTAGTGGGGGCGGGTAGCGGGGACCCCACGCAGTATTTT	95
TGTGCCTGGAGTGTTGGGGAGCAAGAGACCCAGTACTTC	95
TGTGCCAGCAGCAACGGGCGGCGGGGCGGAGTGGGTCCACGAAGAGAGACCCAGTACTT	95
TGTGCCAGCAGCTATGGGGGGGCGCAGACTGAAGCTTTCTTT	93
TGCAGCGTTGGACAGGGGGCGGAAGCTTTCTTT	92
TGTGCCAGCAGCTCAAATCTCCCCGGGACTACCAACACCGGGGAGCTGTTTTTT	91
TGCAGCGTTGAAGATCAAGCGGGGGTGTTCGGCACTGAAGCTTTCTTT	90
TGCAGCGCCGCGGCCCCGGGAGGATGGGAGACCCAGTACTTC	90
TGTGCCAGCAGGCAGGGGATAAACACTGAAGCTTTCTTT	89
TGTGCCAGCAGCGACCCGCAGCTTACGCAGTATTTT	88
TGTGCCAGCAGCCCTAGGACTAGCGGAAACAATGAGCAGTTCTTC	88
TGTGCCTGGAGTGTAGATAACTATGGCTACACCTTC	88
TGCAGTGCTTCGACAGGGGGCCAGCTTGGCTACACCTTC	87
TGCGCCAGCAGCCAAGATGCGAGTGTTTGGGCTAGCAATCAGCCCCAGCATTTT	87
TGTGCCAGCAGCTCCGGAAGGGCTCAGCCCCAGCATTTT	87
TGCAGCGTTACAGGGAATTCCTACAATGAGCAGTTCTTC	85
TGTGCCAGCAGCCCGGGAGTAGCAGATACGCAGTATTTT	85
TGTGCCACCCCTAGGGGTGACAGCGCGAACACCGGGGAGCTGTTTTTT	85
TGTGCCAGCAGGGGGGGGCAGAACACTGAAGCTTTCTTT	85
TGTGCCAGCAGTTCAGCGGAGTACAATGAGCAGTTCTTC	84
TGTGCCAGCAGCTTGGGGTGGATTTCCAATCAGCCCCAGCATTTT	84
TGCAGTGCTAGGGTCCCAGGGGACAGGGACGGTGAGCAGTTCTTC	84
TGCGCCAGCAGCCAAGCCCAGGTGGTTGAGAGCATTCAGTACTTC	84

**TGTGCCACCGGCGTGGGGCAGGAGACCCAGTACTTC**	84
**TGTGCCAGCAGCCTGCCCCCCACAGATACGCAGTATTTT**	83
**TGTGCCAGCAGCTTAGACGCGGGCGAATCCTACGAGCAGTACTTC**	83
**TGCGCCAGCAGCCGCACGGACAGTCAAGAGACCCAGTACTTC**	83
**TGTGCCAGCAGCGCGAGGGTTGACATTCAGTACTTC**	82
**TGCGCCAGCAGCCCTACCCACACAACAGATACGCAGTATTTT**	81
**TGTGCCAGCAGCTTGGATGGGGCGGGAGGAGGAACGAACGGCTACACCTTC**	81
**TGCGCCAGCAGCCAGGGACTAGCGGGATGGGATGAGCAGTTCTTC**	81
**TGTGCCAGCAGCCAAGTCATTGGGGGGCGTAGACCTGAAGCTTTCTTT**	80
**TGTGCCAGCAGCCCAATACTGAACACCGGGGAGCTGTTTTTT**	80
**TGTGCCACCAGCAGAGGCCGGACAGAACCCCTCCACTTT**	80
**TGTGCCAGCAGCTTCGGACGTTACAATGAGCAGTTCTTC**	80
**TGTGCCAGCAGCTTAGCCTACTCAGGGGGCCTCGAGACCCAGTACTTC**	79
**TGTGCCAGCAGCTTAGAGGGACAGGGACCCTACGAGCAGTACTTC**	79
**TGTGCCAGCAGCCTCTCCACAGATACGCAGTATTTT**	78
**TGTGCCAGCAGCTTACCCCCGTGGACAGCAGGCTACACCTTC**	78
**TGTGCCAGCAGCTTCGGACGGTACGACGAGCAGTACTTC**	78
**TGTGCCTGGGCAATCAGGGGCGCCCTAAATTACAATGAGCAGTTCTTC**	77
**TGTGCCAGCAGCTTAGGTGGGAGTACCTACGAGCAGTACTTC**	77
**TGTGCCAGCAGTCAAACAGGGGGAGGGTCTGAAGCTTTCTTT**	77
**TGTGCCAGCAGCTTCCGGCGGGCGGCTAGCACAGATACGCAGTATTTT**	77
**TGTGCCTGGAGTGTAGAAGGGGGAGGACTCCGGGGATCTCAGCCCCAGCATTTT**	76
**TGTGCCAGCAGCTTAGTCAAGGGGGCGGACCAGCCCCAGCATTTT**	76
**TGTGCCAGCAGCCAATGGGGAACAGAAACGACTAGCAATCAGCCCCAGCATTTT**	76
**TGCAGCGCCTCGCTCTCCTGGGGGGGCCAGAACACCGGGGAGCTGTTTTTT**	76
**TGTGCCAGCAGCTTACCTCGAAGGACAGATAATTCACCCCTCCACTTT**	76
**TGTGCCAGCAGGAGGGACGTACATGAGCAGTTCTTC**	76
**TGCGCCAGCAGCCAAGGTACATCCTACAATGAGCAGTTCTTC**	76
**TGCAGCGTTGAGCACTCCTACGAGCAGTACTTC**	76
**TGCGCCAGCACCCCCAGAACTGGCGCAGATACGCAGTATTTT**	75
**TGTGCCAGCAGCATCGGGACTAGCGGGAGTGGGTGGGAGACCCAGTACTTC**	75
**TGTGCCTGGAGTTTCCCCCCCCGGGGACAGGGGGGAAATCAGCCCCAGCATTTT**	74
**TGTGCCAGCAGCCCGGCTAGCGGCAGTCTTGAGCAGTTCTTC**	74
**TGTGCCAGCAGCTCGGGTTGGGAGGGGGGCACTGAAGCTTTCTTT**	73
**TGCGCCAGCAGCCAAGGTCCGGGCGGGAGGTCCTACAATGAGCAGTTCTTC**	73
**TGTGCCTGGAGCCGGGGACAGGGTACCTACGAGCAGTACTTC**	72
**TGTGCCAGCAGCTTAGTAGGAGGGGGTACTAGCTACCAAGAGACCCAGTACTTC**	72
**TGTGCCAGCAGCCGGGACAGAAGCTCCTACGAGCAGTACTTC**	71
**TGCAGTGCTAGAGTCTACAATGAGCAGTTCTTC**	71
**TGCAGCGCCGGGGGGCTAGCGGGAGGTCTGGGGAACAATGAGCAGTTCTTC**	71
**TGCGCCAGCAGCCAAGATGATCGGGTGGGAGGGTCCCAGTTCTACAATGAGCAGTTCTTC**	70
**TGTGCCAGCAGCTTTCAGCGGGGGGCGTACAATGAGCAGTTCTTC**	70
**TGCGCCAGCAGCCAGTCGTGGCAGAGCACAGATACGCAGTATTTT**	70
**TGCGCCAGCAGCCAAGACTCCAGGGTACTAGGTGGCTACACCTTC**	69
**TGCAGCGTTGGCGAGCGGGGGACCCAGTACTTC**	69

**TGTGCCTGGAGTCGGGGGGGCGTAGGCGGGGAGCTGTTTTTT**	68
**TGTGCCAGCAGCCCCGTGGGCGGAGAAGAGACCCAGTACTTC**	68
**TGTGCCAGCAGCTTAGCCGGGACAGGTTCCTACTATGGCTACACCTTC**	68
**TGTGCCAGCAGCTTATATTCAGGGAGGGAGACCCAGTACTTC**	68
**TGTGCCAGCAGCCCCGGACAGGGGGCCAGCGGGGAGCTGTTTTTT**	68
**TGTGCCAGCAGCTTAAATCCCGGGGGCGCTGGCTACACCTTC**	67
**TGCGCCAGCAGCCAAGATACTTCCAGGGGGGAGACCCAGTACTTC**	67
**TGTGCCAGCAGCTTAGTTGACTTCTACGAGCAGTACTTC**	67
**TGTGCCAGCAGTTCGGTCAATCAGCCCCAGCATTTT**	67
**TGTGCCAGCAGCAGGGGACTAGCGAACAATGAGCAGTTCTTC**	66
**TGCAGTGCTAGGGATTCCTCAATCGGCCCGAATCAGCCCCAGCATTTT**	66
**TGTGCCAGCAGCTTAATCGGGTTTAGTTACTTC**	66
**TGTGCCAGCAGCCCCCAGACAGGGGGCTATGGCTACACCTTC**	66
**TGTGCCAGCAGCGACCTAAGGGAGGACTATGGCTACACCTTC**	65
**TGCAGCGCCTCGCCCCCTATAGCGGGAGGGGCCCAGTACTTC**	65
**TGTGCCAGCAGACGCCGGCAGGCTGGCCAAGCTTTCTTT**	65
**TGTGCCAGCAGCCTAGGGGGGTTCAATCAGCCCCAGCATTTT**	65
**TGTGCCAGCAGCTTTGACAGATACAATGAGCAGTTCTTC**	65
**TGCAGCGTGGGACAGGGACCTTTTCAGCCCCAGCATTTT**	65
**TGCAGCACCTCCGGGGGGGGCACAGATACGCAGTATTTT**	64
**TGCAGCGTTGAACCACAAGGGCAGGCGTCGAGAGAGACCCAGTACTTC**	64
**TGTGCCAGCAGCTTAGGTCGGGCGGGAGGGCCAAATGAGCAGTTCTTC**	64
**TGTGCCAGCAGCACCCACCTAGATACCGGGGAGCTGTTTTTT**	64
**TGTGCCAGCAGCCCGAGGGGCGGGAGGGCCGCAGATACGCAGTATTTT**	63
**TGCAGCGTTGCAGACAGCTTCCCGGCAAACATTCAGTACTTC**	63
**TGCGCCAGCAGCCAAGGACTTACGTGGCAAGAGACCCAGTACTTC**	63
**TGTGCCAGCAGCATTCCGACAGGGGGAGCGGGCAATACGCAGTATTTT**	63
**TGTGCCAGCAGCCAAGAGGGACTAGCGGGAGGGGGTTACAATGAGCAGTTCTTC**	62
**TGCAGTGAGGCGGGTGGGAACACTGAAGCTTTCTTT**	62
**TGCAGCGTTAGTGGGCAGGCGGGCAATCAGCCCCAGCATTTT**	62
**TGTGCCAGCAGCTTTGGAAGGGAAGAGACCCAGTACTTC**	62
**TGTGCCACCAGCGGTAGACAGGGATGGGATCAGCCCCAGCATTTT**	62
**TGTGCCAGCAGCTTAAGACAGGGGGCCGGGGAGCTGTTTTTT**	62
**TGTGCCAGCAGTCCCGGGCCGGGGGCCCACTCCTACGAGCAGTACTTC**	61
**TGTGCCACCAGCAGACCCTCACAGGGGTCTCCGGTCGAGCAGTACTTC**	61
**TGTGCCAGCAGCCTACCGACAGATTTCTACGAGCAGTACTTC**	61
**TGTGCCAGCAGTGGGGGACAGGCCGAGACCCAGTACTTC**	59
**TGTGCCAGCAGCTTGGTTTCAGGGCAGTACCCTACCTACGAGCAGTACTTC**	58
**TGTGCCAGCAGTTGGACAGGGGGCGTTTACGAGCAGTACTTC**	58
**TGTGCCAGCAGCTTTCTTCAGGAGCAGTTCTTC**	58
**TGTGCCAGCAGCCAAGATCGTGGAGGGACCTACGAGCAGTACTTC**	58
**TGTGCCAGCAGCTTAACAGGGGGCATTTACGAGCAGTACTTC**	58
**TGTGCCTTCCGGGACACATCCTACGAGCAGTACTTC**	57
**TGCGCCAGCAGCCAAGACCGAAAGCCCGCTGGGGCCAACGTCCTGACTTTC**	57
**TGTGCCAGCAGTTTGGTACAGGGGGTTGAAAAACTGTTTTTT**	57

**TGCAGCGTTGAAGATGGGGGTCATGAGCAGTTCTTC**	57
**TGTGCCTGGACGGCCCCGACCGTCGGGAGCGCCGGGGAGCTGTTTTTT**	57
**TGTGCCAGCAGCTTAGGTCCAGGGGGCCTTTTCTCTGGGGCCAACGTCCTGACTTTC**	56
**TGTGCCAGCAGCACCTATCCACCGGGACAGGGGGCGCGCGAGCAGTTCTTC**	56
**TGTGCCTGGAGTCGGGACAGACAGGGGGCCAACGTCCTGACTTTC**	56
**TGTGCCAGCAGCTTATCCGGGGCGGACATCAATGAGCAGTTCTTC**	56
**TGTGCCTGGAGTGTAAAGGGGGCTGGGGAGACCCAGTACTTC**	56
**TGTGCCAGCAGCTTAGCGGGCAGTAATTCACCCCTCCACTTT**	56
**TGTGCCAGCAGCCCGGGGACAGGGGTCGAGCAGTACTTC**	56
**TGTGCCAGCAGCTCCGGGGTATCCACAGATACGCAGTATTTT**	56
**TGTGCCTGGAGCGACCGGGGACCGGGAGGGTCACCCCTCCACTTT**	56
**TGTGCCTGGACGCGGGGGAACACTAAGGAGCAGTACTTC**	56
**TGTGCCAGCAGCTTAGTACTCGCGGGAGGGTCGTATGAGCAGTTCTTC**	55
**TGTGCCAGCAGCACGAACACAGGTGGCCAGCATTTT**	55
**TGTGCCAGCAGCTCGACTAGCAGCACAGATACGCAGTATTTT**	55
**TGTGCCAGCAGCCCCGGACAGGGAAACAATGAGCAGTTCTTC**	55
**TGTGCCAGCAGCCAGGGGGCGGGACTTTACACAGATACGCAGTATTTT**	55
**TGTGCCACCAGCCGGGGAGGGGGCACTGAAGCTTTCTTT**	55
**TGTGCCAGCAGCTTTAGACTAGACAATGAGCAGTTCTTC**	54
**TGCGCCAGCAGCCCCCGAAGGGCGGGAGGGGACAATGAGCAGTTCTTC**	54
**TGCAGTGCTAGATCCTGGACAGGGCAGGGCAATCAGCCCCAGCATTTT**	54
**TGCAGTGCTTCGACAGGGGGCGCAGATGGCTACACCTTC**	54
**TGTGCCTGGAGGAAGGCGGGAGGTATCTACAATGAGCAGTTCTTC**	54
**TGTGCCAGCAGTCAGGGGGACCAAGCCTACAATGAGCAGTTCTTC**	53
**TGTGCCAGCACCGCGGTAGCGGGAGGTTACAATGAGCAGTTCTTC**	53
**TGTGCCAGCACCTTAGGTCAGGGCTGGACTGAAGCTTTCTTT**	53
**TGTGCCAGCAGCTTATATGGAGACGGACGAAGCACAGATACGCAGTATTTT**	52
**TGCAGCGGCTCGACTCTAGCGAACAATGAGCAGTTCTTC**	52
**TGTGCCAGCAGCGGACCCATACCCTACGAGCAGTACTTC**	52
**TGTGCCTGGGGCGCCACCGGGACTAGCGGGTTGGTTGTC**	52
**TGCAGCGCCGACGCACAGGGCTCGGGGGAGCTGTTTTTT**	52
**TGTGCCAGCAGCCCTCAAACAGGGGGCGGGTCCTACAATGAGCAGTTCTTC**	52
**TGTGCCAGCAGCGTGAGGTCGTCGAAGTTCTTC**	51
**TGTGCCAGCAGCCTAGAGATTAGCAAGACAGATACGCAGTATTTT**	51
**TGCAGTGCTAAGGACCCACTGAGAGATACGCAGTATTTT**	51
**TGCAGCGTTGAAGTAGGAGGGGGAGTACGGTTCTTC**	51
**TGCGCCAGCAGCCAAGAAGCCAGGGGATTAGACACCGGGGAGCTGTTTTTT**	50
**TGTGCCAGCAGCTCGGGACTAGCGAACACCGGGGAGCTGTTTTTT**	50
**TGCGCCAGCAGTCCGGGACTAGCGCGAACCTATCGCGATGAGCAGTTCTTC**	50
**TGCGCCAGCAGCCAAGCTCATCCCGGGACAGGGCAGAATTCACCCCTCCACTTT**	50
**TGTGCCAGCAGCTTCGGGGCACCCGTTGGTGAAGCTTTCTTT**	50
**TGTGCCAGCAGACCTGGGACCGCCCGGGAGGCGGCTTTCTTT**	50
**TGTGCCAGCAGTCGAGGAGTCCAAGAGACCCAGTACTTC**	50
**TGCAGTGCTAGTACACAGGGGGCGAGTGGCTACACCTTC**	50
**TGTGCCTGGTCAACGTTAAATGAGCAGTTCTTC**	50

TGTGCCAGCAGCCCCCGCCAAGGGAACACCGGGGAGCTGTTTTTT	50
TGCGCCAGCAGCCAAGATCCTAGCGGGAGGACAATTTCCTACGAGCAGTACTTC	50
TGTGCCAGCAGCTCCCTCGGGACAGGGTCCGATGAGCAGTTCTTC	49
TGTGCCAGCAGCTTAGTACTAGCGGGAGCCGAGCAGTACTTC	49
TGTGCCAGCAGCTTACTCGCTAGCGGGGGGAATGAGCAGTTCTTC	49
TGTGCCAGCACCCCTGAGGGGGGGAACACTGAAGCTTTCTTT	49
TGTGCCAGCAGCCCGCGACCCACACAGGGGAAAGGCTACACCTTC	49
TGTGCCAGCAGTCACCAATGGGGCGAGCAGTACTTC	49
TGCGCCAGCAGCTTGCTGTCTAACTACGAGCAGTACTTC	49
TGCGCCAGCCGACGGGACGGCTACGAGCAGTACTTC	49
TGCAGTGCCGATGGACTAGCCAACTACGAGCAGTACTTC	49
TGCAGTGCTAGCCCGACCGGGGTGGGTGGGGCCAACGTCCTGACTTTC	48
TGTGCCGGCGGACCTAGCTCCATCAACACCGGGGAGCTGTTTTTT	48
TGCAGTGCTGCGGTCCTTAGCAATCAGCCCCAGCATTTT	48
TGTGCCAGCAGCTTAGCTGGGACTAGCGAGACAGATACGCAGTATTTT	48
TGTGCCAGCAGCATCGGACTAGCTAGAGATACGCAGTATTTT	48
TGTGCCAGCAGCCCTTATGGCGGCAATCAGCCCCAGCATTTT	48
TGTGCCTGGGAGAACTCTGGGGCCAACGTCCTGACTTTC	48
TGCAGTGCTAACCCCACAGGGGAGAGCAATCAGCCCCAGCATTTT	48
TGCAGCGTTGAGGACCCGCCGGGCGCAGATACGCAGTATTTT	48
TGTGCCAGCAGTTACGCGGGAGACTCCTACGAGCAGTACTTC	48
TGCAGCGTGAAGTCCCCCCTAGCGGGAGGGCCCTTGTTCAGCACAGATACGCAGTATTTT	47
TGTGCCAGCAGGGCCGGGACTGACATGAACACTGAAGCTTTCTTT	47
TGCGCCAGCAGCTTGGAAGGACCAGGACAGGGTCGTTCAGATACGCAGTATTTT	47
TGTGCCAGCAGCTTAGCGAGGAATCAGCCCCAGCATTTT	47
TGTGCCAGCAGCTTAAATGGTGGGACCGCCCTGGAGCAGTTCTTC	46
TGTGCCAGCAGCTTACAGCCCGGGACCTTCAATGAGCAGTTCTTC	46
TGTGCCAGCAGCACCGGTCTCCGAGCATGGGAGACCCAGTACTTC	46
TGTGCCAGCAGCTTTGGCGTGTCGTACGAGCAGTACTTC	46
TGCAGCGTCGCCCGGCAGGACCAGCAGTTCTTC	46
TGCGCCAGCAGCCTCAGACGCGTTCCGGGACTAGCGGGAGGAGGGGATGAGCAGTTCTTC	45
TGCGCCAGCAGCTCCCCGGGACAGCCAAATGGCTACACCTTC	45
TGTGCCAGCAGCTTAGCGAGGGTTCGCGAGCAGTACTTC	45
TGCAGTGCCACCGATCCAGACAGTGAGCAGTTCTTC	44
TGTGCCAGCACCCTCACAGGGTCTAGATCTGAAGCTTTCTTT	44
TGCAGTGCTAGAGTTGCGGGAGGGGGACAAGAGACCCAGTACTTC	44
TGTGCCAGCAGTTACGGGGGCCCCGGGACAGCCTACGAGCAGTACTTC	44
TGTGCCAGCAGCCGTGGACAGTACGACGAGCAGTACTTC	44
TGTGCCAGCAGCTTAGAAGGTATTGGGGGACGGAACCAGCCCCAGCATTTT	44
TGCAGCGTTAAGACAGGGGGAGCGAGGCAAGAGACCCAGTACTTC	43
TGCAGCGTAGACAGCACAGATACGCAGTATTTT	43
TGTGCCAGTACCGTCGGGGCTAGCGGTTCTAGCACAGATACGCAGTATTTT	43
TGCAGCGTACTCCGGGACCCTAATTCCTACGAGCAGTACTTC	43
TGTGCCAGCAGCCAAGGGGCTAGCGGGAGGGCCTACGAGCAGTACTTC	43
TGTGCCAGCAGCATAGCGGGGGAATACGAGCAGTACTTC	43

**TGCAGTGCTCCGGAACAGAACACTGAAGCTTTCTTT**	43
**TGCGCCAGCAGTGAAGCCGCAGATACGCAGTATTTT**	42
**TGCGCCAGCAGCCAAGATTCCTCGATAGGCGGGGAGCTGTTTTTT**	41
**TGTGCCAGCCCCCTAGGCGGGAGGAACCCCGGGGAGCTGTTTTTT**	41
**TGTGCCAGCAGCCCAATTAGACAGCCCCAGCATTTT**	41
**TGTGCCTGGATGGGGGAAGATAATTCACCCCTCCACTTT**	41
**TGTGCCAGCAGCTCAGCCGGCGGTGATGGCGTAAAACTGTTTTTT**	41
**TGCAGCGTTGATCTATTCGATACGCAGTATTTT**	40
**TGTGCCAGCAGGGTCGACAGAAACTATGGCTACACCTTC**	40
**TGTGCCAGCAGCGCATGGACAGGGTCGAATCTCAATGAGCAGTTCTTC**	40
**TGTGCCAGCAGCTTAGGGACAGCTAACACCGGGGAGCTGTTTTTT**	40
**TGTGCCAGCAGCTTAGAGGGCAGGTATAACGAGCAGTACTTC**	40
**TGTGCCAGCAGCTTAGCGATAGGAGAGACCCAGTACTTC**	40
**TGTGCCAGCAGCTTAGTCCGGCAACCTCTTGGAGCTTTCTTT**	40
**TGCAGTGCTAGATTTAAACCTGAGCAAGAGACCCAGTACTTC**	40
**TGCGCCAGCAGCCAAGACTATAGGAGCATTTATGGCTACACCTTC**	39
**TGCAGTGCTAGTAATAAGGACAGTCGCTACGAGCAGTACTTC**	39
**TGTGCCAGCAGTTTGGGAGCCGTCCCTGAAGCTTTCTTT**	39
**TGCGCCAGCAGCCAAGAACTCACCCATGAGCAGTTCTTC**	39
**TGTGCCAGCAGTTACTCGATGCTTCCAGGGACTGGGAATTCACCCCTCCACTTT**	39
**TGCAGCGTTGAAGATCGGGGCCAGGGGGGTGGAGGCTACACCTTC**	39
**TGCAGCGTTGAAGAGACCGGGACAGGGACCTACGAGCAGTACTTC**	39
**TGCAGTGCCATCACGGCGGGGGGGTCAGATACGCAGTATTTT**	38
**TGCAGTGCTTCCCCGGGACTAGCGTTGGATACGCAGTATTTT**	38
**TGCGCCAGCAGCGGCAGTTTTTCCTCCTACGAGCAGTACTTC**	38
**TGTGCCAGCAGCTTAGCTTTAGGGGGCAACGGAAACACCATATATTTT**	38
**TGTGCCAGCAGCTCCTATAGCGGGAAGAACACCGGGGAGCTGTTTTTT**	38
**TGCAGTGCTAGTGGAAAGGGAACTCTGCTGACCCAGTACTTC**	38
**TGTGCCAGCAGCCCCCGACAGGCAAGCTATGGCACTGAAGCTTTCTTT**	38
**TGCAGTGCTAGAGATGGGGCAGGGAAGGGCTACACCTTC**	37
**TGTGCCACCTCCATGGGGGGCACGGCCAAAAACATTCAGTACTTC**	37
**TGCAGTGCTAGGGTCCCGGGACAGGCGTATAGCAATCAGCCCCAGCATTTT**	37
**TGCAGTGCTCCCCCCCAAGGAGAGACCCAGTACTTC**	37
**TGTGCCAGCAGCTGGTCTGGACAGGGGAATAATTCACCCCTCCACTTT**	37
**TGCAGCTTTAGGGACCTAGCGAACACCGGGGAGCTGTTTTTT**	37
**TGTGCTAGTGGGAGCGGGTCGAACACCGGGGAGCTGTTTTTT**	36
**TGCAGCGTTGTAAGGGACATGGGCCGGGACGAGCAGTACTTC**	36
**TGTGCCTGGTATCCGGGACAGGCAAACACTGAAGCTTTCTTT**	36
**TGTGCCAGCAGTCCGGGACTAGCAAGCGGGGAGCTGTTTTTT**	36
**TGTGCCAGCAAAGTAAGGGGAAACACTGAAGCTTTCTTT**	36
**TGTGCCAGCAGCTTAGCTAACGGTTACGAGCAGTACTTC**	36
**TGTGCCAGCATCTCATCTGGAAACACCATATATTTT**	36
**TGCGCCAGCAGCTTCAGTCCGGCTAGCACAGATACGCAGTATTTT**	36
**TGCAGTGCTAGAGACAGGGTAGCGACAGAGACCCAGTACTTC**	36
**TGTGCCTGGAGTCGGGGGCGGGAGGGCCCCTACGAGCAGTACTTC**	36

**TGTGCCAGCAGCATGACTAGCGGGAGTGGCGATACGCAGTATTTT**	36
**TGTGCCAGCAGCCAAGGACTAGGCCCAGCAACGCAGTATTTT**	36
**TGTGCCAGCAGCTTAGAACTAGCCTCCTACAATGAGCAGTTCTTC**	36
**TGCAGCGGGGACGGAATGAACACTGAAGCTTTCTTT**	35
**TGCAGTGGGGTTCCCCGGAACACGCAGTATTTT**	35
**TGCGCCAGCAGCCAAGAGGACGGCGGGAGGCCGACCTACGAGCAGTACTTC**	35
**TGTGCCAGCAGCCAATACGAGACCCAGTACTTC**	35
**TGCGCCAGCAGCCAAGATGCTTCGGGGACAGGGGGCAAAAATGAGCAGTTCTTC**	35
**TGCGCCAGCAGCTTGGGCACACAAGAGACCCAGTACTTC**	35
**TGTGCCACCAGCTTGGTTTTATCGTACACTGAAGCTTTCTTT**	35
**TGTGCCAGCAGCTTAGCTTACAGGGGGGGATCTAGCTCTGGAAACACCATATATTTT**	35
**TGTGCCAGCAGCCTGACAGGGGGAGAGACCCAGTACTTC**	35
**TGTGCCAGCAGCACTCGGACAGGGGGGGATCAGCCCCAGCATTTT**	35
**TGCAGCGTTGACGCCGGGACAGGGGGCGTCACTGAAGCTTTCTTT**	34
**TGTGCCACCAGCCCCCTAGCGGGAGGCCCCACCGGGGAGCTGTTTTTT**	34
**TGTGCCAGCAGCGTAGCGGCGGACGCCTCCTACGAGCAGTACTTC**	34
**TGTGCCAGCAGCTTAGCGGGACTCCGTGGCGATGAGCAGTTCTTC**	34
**TGCAGTGCTAGCTTCGGACAGGTCTTCACAGATACGCAGTATTTT**	34
**TGTGCCAGCAGCTTAGCGCGAGGTAGGAGTGAGCAGTTCTTC**	34
**TGTGCCAGCAGACCCATCGGACAGGGTTACGAGCAGTACTTC**	34
**TGTGCCAGCAGCTCCCGGGGTAGCGGGAGTTCGGGCCGGCCAGATACGCAGTATTTT**	34
**TGTGCCTGGCACCGGACCGCGACGATGTACAATGAGCAGTTCTTC**	34
**TGCGCCAGCAGCCAAGATAGGGTGCGGGTCGCCCCAGATACGCAGTATTTT**	34
**TGTGCCAGCAGCTCTGGGTCTGAAGCTTTCTTT**	33
**TGTGCCAGCAGCTACAGGACAGGGTCGAGCACTGAAGCTTTCTTT**	33
**TGTGCCTGGAGTGTACGGGAACGGGAGACCCAGTACTTC**	33
**TGTGCCTGGAGTCCACTCCGCGAGCAGTACTTC**	33
**TGTGCCAGCAGTCCCGGGACAGGGGTCAATCAGCCCCAGCATTTT**	33
**TGTGCCAGCAGCTTGAATCCGGGAGGAACCTACGAGCAGTACTTC**	33
**CGTGCCAGCAGCTTTCCATTACCAATCAGCCCCAGCATTTT**	33
**TGTGCCAGCAGCCCCGGGACAGCTAAGAACACTGAAGCTTTCTTT**	33
**TGCAGTGCCACCGGGACAGGGATCGAGACCCAGTACTTC**	33
**TGTGCCAGCAGCCCCGTAGGACAGGGCTTTTTCTCTGGGGCCAACGTCCTGACTTTC**	33
**TGCGCCAGCAGCTTGTACGGCCTCGCACGAGAGACCCAGTACTTC**	33
**TGTGCCAGCAGCCAAGACCCTGGACAGGGGAATGAAAAACTGTTTTTT**	33
**TGCGCCAGCAGAATGGTACCCTCCGGGAACACTGAAGCTTTCTTT**	33
**TGCAGCTGGGACACTGAAGCTTTCTTT**	33
**TGTGCCAGCAGCGAAGTGCCGCAGGGGCCCCAGCATTTT**	33
**TGTGCCAGCAGCCCCCAACAGACCCAGTACTTC**	32
**TGCGCCAGCAGCCAAGGGGTGGGGGCGGGGGACAATGAGCAGTTCTTC**	32
**TGTGCCTGGAGTTTAAGGGGGGATTCACCCCTCCACTTT**	32
**TGTGCCAGCAGCCGGATAACAGAGTCTGAGCTGTTTTTT**	32
**TGCGCCAGCAGCCAAGAGGCCAGGGGGGGTACAGATACGCAGTATTTT**	32
**TGCAGCGTTGAAGACCGGGACAGGGGAAATCTCTACGAGCAGTACTTC**	32
**TGTGCCAGCAGCAATAGCGGGCACTACGAGCAGTACTTC**	32

**TGTGCCAGCAGCTTAGTGCAGGGGTCGGACAGACCCTACGAGCAGTACTTC**	32
**TGCAGCGTTTTGAGGGACTATGAAGGAGAGACCCAGTACTTC**	32
**TGTGCCAGCAGCTTAGTGGCTAGCGGGAGTAACGAGCAGTACTTC**	32
**TGTGCCAGCAGCTTATACGGGGGGGGGCGAGAAACTGAAGCTTTCTTT**	32
**TGTGCCACCAGCTCCACGGGACTTCAAGAGACCCAGTACTTC**	31
**TGCAGCCCTAGCGGGAGCTCCTACAATGAGCAGTTCTTC**	31
**TGTGCCAGCAGCCCGACGGATGAAAAACTGTTTTTT**	31
**TGCGCCAGCAGCTTGGCGGACTACACAGATACGCAGTATTTT**	31
**TGTGCCAGCAGAATGAGACTAGCGGGAGGGGCAGATACGCAGTATTTT**	31
**TGCAGTGCTACCTACCGGGACAGGGGGCCGGGGGAGCAGTACTTC**	31
**TGTGCCAGCAGCCGGATGACAGGGGTGCAAGAGACCCAGTACTTC**	31
**TGTGCCAGCAGTACCGGCGGCAGCAACTATGGCTACACCTTC**	31
**TGCAGCGTTGAACAGGGGGCGCTGGGCACAGATACGCAGTATTTT**	31
**TGCAGTGCCATAAGGGACAGCGGGAACTATGGCTACACCTTC**	31
**TGCGCCAGCAGCTTGGAGGTCCTGACTTTC**	30
**TGTGCCAGCAGCTTTTACCGGGACAGGGACTATGGCTACACCTTC**	30
**TGCAGTGCTAGAGATCCAGGGGGCACTGAAGCTTTCTTT**	30
**TGCGCCAGCAGCTTTTTGGGCTACGAGCAGTACTTC**	30
**TGTGCCAGCAGCCAGGAAACGAGTGCTTTCTTT**	30
**TGTGCCAGCAGCCTGAATAACTATGAGTACACCTTC**	30
**TGCGCCAGCAGCCAAGGTCGAACAGGGGGCCACTATGGCTACACCTTC**	30
**TGTGCCAGCACTTCTTTGGGGGGAGGGTTGAACACTGAAGCTTTCTTT**	30
**TGTGCCAGCAGCCCGGGACAGGCGCTCGGGGAGCTGTTTTTT**	30
**TGCGCCAGCAGCCAAGATTGGGGGGGCGAGTCACCCCTCCACTTT**	30
**TGTGCCAGCAGCAGTACTAATTCAGATACGCAGTATTTT**	30
**TGTGCCAGCAGCAAGGGACTAGCGACAAGCGGGGAGCTGTTTTTT**	30
**TGTGCCAGCAGCTCCCTCCAAGCGGGAGGCCGGAATGAGCAGTTCTTC**	30
**TGTGCCAGCAGCCCGCAGGGCCCCTATGGCTACACCTTC**	30
**TGTGCCAGCAGCATCCTGGGTGGGACAGGGTCCTACGAGCAGTACTTC**	29
**TGTGCCAGCAGCATCCTCCCCCGGGGCTTTTTAGCCAACAATGAGCAGTTCTTC**	29
**TGCAGTGCTAGTGACGAGGGGAGGTCATACGAGCAGTACTTC**	29
**TGTGCCAGCAGCTTAGGATACAGGGGAGGAGAACAAGAGACCCAGTACTTC**	29
**TGTGCCAGCAGTTACCGCAGCTCCACAGATACGCAGTATTTT**	29
**TGTGCCAGCAGCTTAAGGGGGGACAGGGACACTGAAGCTTTCTTT**	29
**TGTGCCAGCAGCTTAGCCTTCGGTCAGGGGGCTGGTGGCTACACCTTC**	29
**TGCAGTGCTAGCACTAGCGTCCCTCCTGAGGAGACCCAGTACTTC**	29
**TGCAGCGTACAACGACTAGCGGGAGAAGAAGACAATGAGCAGTTCTTC**	29
**TGCGCCAGCAGCTTGGGGATTTCCAGAGAAGGTGGCTACACCTTC**	29
**TGCGCCAGCAGCCAAGCCCGGGGGGAGACCCAGTACTTC**	29
**TGCAGCGTAGCCCAGGGGTCAGATACGCAGTATTTT**	29
**TGCGCCAGCAGCCTATCTATGGGCCAAGAGACCCAGTACTTC**	29
**TGTGCCTGGGGGGGGGCAGGGAATCAGCCCCAGCATTTT**	29
**TGTGCCAGCAGCTACCAAATGACAGGCTATGGCTACACCTTC**	29
**TGCAGCGTTGCGCGACAGGGGGCGAGAGAGCAGTACTTC**	29
**TGCAGTGCTATCCCGAGGGATACGCAGTATTTT**	28

TGTGCCACCAGCAGAGGAGGGAGTCGGCAAGAGACCCAGTACTTC	28
TGCAGTGTCCAAGAATACAATGAGCAGTTCTTC	28
TGCAGCGTTCGTCCGACTAGCGGGGGAGCAGATACGCAGTATTTT	28
TGTGCCAGCAGCCAAGGGCCCTGGGACAGGGTTGAATATGGCTACACCTTC	28
TGCAGTGCTATGGGGGTCCCGGGACAGGGGGCGGTTGAGCAGTTCTTC	28
TGTGCCAGCAGTTTAACTAGCGGGATCCACAATGAGCAGTTCTTC	28
TGCGCCAGCAGCTTGGCCGAAGGGGACGCTCCTGGCCAGTACTTC	28
TGCAGTGCTAGAAGGACTAGCGTCTACGAGCAGTACTTC	27
TGTGCCAGCAGCTTAGACGGAGGGGACAGGAATGAGCAGTTCTTC	27
TGCGCCAGCAGCCCTCCGACTAGATTTAATGAGCAGTTCTTC	27
TGTGCCACCAGCGCCAGACAGGGAACCGAGCAGTACTTC	27
TGTGCCAGCAGCCCCGGACAGGGTGGGTATGGCTACACCTTC	27
TGTGCCAGCAGCTTTGCAGATACGCAGTATTTT	27
TGTGCCAGCAGCCTAACGGGGGGCACTAATGAAAAACTGTTTTTT	27
TGTGCCAGCAGACCCGGGACAGGGGGATCCTACGAGCAGTACTTC	27
TGTGCCTGGAGCGCAATGGAGGATTCACCCCTCCACTTT	27
TGCAGTGCTAGCGGGGGACAGGGGTTCTCCACAGATACGCAGTATTTT	27
TGTGCCAGCAGCCCCAACATAGGGGACTCTGGGGCCAACGTCCTGACTTTC	27
TGTGCCAGCAGCCCCACGGATGAGACCCAGTACTTC	27
TGTGCCAGCAGTTACACTAGCGGGAGGCTAGATACGCAGTATTTT	27
TGTGCCAGCAGCCCCTTTATGGGCAACACTGAAGCTTTCTTT	27
TGCAGTGCTGGACAGTCTAGCAATCAGCCCCAGCATTTT	27
TGTGCCAGCAGTTACTCGCGAACCAATCAGCCCCAGCATTTT	27
TGTGCCAGCAGCTTAGGGGGGACGCTAAACGGCTACACCTTC	26
TGCGCCAGCACCCTAGGGGGACTAGTGACCTACAATGAGCAGTTCTTC	26
TGCAGTGCCCCCCTTAGACTAGCGGGAGGGTACACAGATACGCAGTATTTT	26
TGCGCCAGCAGCTTGGGTGACACAACCCGGGAGCCCCAGCATTTT	26
TGTGCCAGCAGCTTAGTGCGGGCGGGAGGCCGAGAGACCCAGTACTTC	26
TGTGCCACCAGCAAAGGACAGAACACAGATACGCAGTATTTT	26
TGTGCCAGCAGCGTCGGACTAGCGGTTAGGGATGAGCAGTTCTTC	26
TGTGCCAGCAGCTTAACAGCTAGCGGGAGGGCCCTTTTC	26
TGTGCCAGCAGCCCCTGGACAGGGGGAACTGAAAAACTGTTTTTT	26
TGTGCCTGGGGGGGACGTAGGTCCGAGCAGTACTTC	26
TGTGCCAGCATTTACGGGACAGCTTCCTACGAGCAGTACTTC	25
TGTGCCAGCACCCTGACAGGACAGGTCAGCTCCTACGAGCAGTACTTC	25
TGTGCCAGCAGCTTAAGTAGGTCCGCCACCGGGGAGCTGTTTTTT	25
TGTGCCAGCAGCTTACCGGGTTACAATGAGCAGTTCTTC	25
TGTGCCAGCAGTCGTAGACTAGCGGGATCGGCAGATAGCTCCTACGAGCAGTACTTC	25
TGTGCCAGCAGCCCGACCACAGGGGGGAGGACAGATACGCAGTATTTT	25
TGTGCCAGCATGGGCGGGACCGCCTACGAGCAGTACTTC	25
TGCAGTGCTAGTGCCCCTGGCTACAATGAGCAGTTCTTC	25
TGTGCCAGCAGTGAACAGGGATCTGGCAAAAACATTCAGTACTTC	24
TGCAGCGTTTGTCCGGGACTAGCAGCGACAGATACGCAGTATTTT	24
TGTGCCAGCAGCTATGGGGCTAGCGTCGGGGAGCTGTTTTTT	24
TGCGCCAGCAGCCTCGTGAGTTCGAACAACTATGGCTACACCTTC	24

**TGTGCCAGCAGCTTCTTAGCGGGAGGATTCGCAGATACGCAGTATTTT**	24
**TGTGCCTGGAGCGGGATCCGGCGAAACAATGAGCAGTTCTTC**	24
**TGTGCCAGCAGTTTATGGGACGGGGCGAACACCGGGGAGCTGTTTTTT**	24
**TGCAGCGTTGAAGTCAGTTACGAGCAGTACTTC**	24
**TGTGCCAGCAGACAAGCGGGGTCATCCTACAATGAGCAGTTCTTC**	24
**TGTGCCAGCAGCCAAGATTGGGGACTAGCGGGCGGCAATGAGCAGTTCTTC**	23
**TGTGCCACCAGCAGAGATGGTAGCTATGGCTACACCTTC**	23
**TGCAGTGCTAGCGATCCCCTAGGAGAGACCCAGTACTTC**	23
**TGTGCCAGCAGCTTAAGTCAGGTAATGAACACTGAAGCTTTCTTT**	23
**TGTGCCAGCAGTTACGGGCTAGCGGGCAAAAACATTCAGTACTTC**	23
**TGTGCCAGCAGCTCCGGACAGGCCCTAAATTATGAAAAACTGTTTTTT**	23
**TGTGCCAGCAGCGTAGACCGGGGGGCTATGGGGAATGAGCAGTTCTTC**	23
**TGTGCCAGCAGGGGACAGGGGGCGGTGGCCCTCCACTTT**	23
**TGCAGCGGGACTAGCGGGGCCGATGAGCAGTTCTTC**	23
**TGTGCCAGCAGCGATACTAGCGACGCGCAAGAGACCCAGTACTTC**	23
**TGTGCCAGCAGCTTAGTAGCGGCCCTCAGGGATACGCAGTATTTT**	23
**TGTGCCACTCTACGGGAGCAAAACACTGAAGCTTTCTTT**	23
**TGTGCCAGCAGCCGTTCTGTCGACAATGAGCAGTTCTTC**	23
**TGTGCCAGCACCCGGGTTAGCGTGAACACTGAAGCTTTCTTT**	23
**TGCAGTGCTAGATCCGACAGTACGAACACTGAAGCTTTCTTT**	23
**TGTGCCTGGAGCCGGCTAGCGGGGGTCTACGAGCAGTACTTC**	23
**TGCAGCGTTGGGGTGGGGGGAGATACTGAAGCTTTCTTT**	22
**TGTGCCACCACCTTTTCTAGCGGGCGGGGGGCGAGCACAGATACGCAGTATTTT**	22
**TGCGCCAGCAGCCAAGTCTCTCTATCTCGGGACAGGAACACTGAAGCTTTCTTT**	22
**TGTGCCAGCATCCCGCGGGGAGGGTACGGTTCACCCCTCCACTTT**	22
**TGTGCCAGCAGCCAAGCAGGGAACTACGAGCAGTACTTC**	22
**TGTGCCTGGGGACAGGGGGCAGGGAAGTCTTCGGAGACCCAGTACTTC**	22
**TGCAGCGTTGAATATTCCGGGGATCAGCCCCAGCATTTT**	22
**TGTGCCAGCAGCCAAGGGCGGTACTCTGAAGCTTTCTTT**	22
**TGCAGTGCGAGCGGCAGGGTGGAGTGGGCTTTCTTT**	22
**TGCGCCAGCAAGGACAGTCACACTGAAGCTTTCTTT**	22
**TGTGCCAGCAGCTTGGAACAGGGGAGGGGGAACACTGAAGCTTTCTTT**	22
**TGCAGTGCCGAAGGCAGGGGAGAGACTGAAGCTTTCTTT**	22
**TGTGCCAGCAGTCTACAGCCGGGACAGGGGTACGAGCAGTACTTC**	22
**TGCGCCAGCAGCGCTGGACAGGCCGCTTCAGATACGCAGTATTTT**	21
**TGCGCCAGCAGCCAAGATGAGGGAAAGACCCAGTACTTC**	21
**TGTGCCAGCAGCCCTTTCGGGGGCAGGGAGGAGACCCAGTACTTC**	21
**TGCGCCAGCAGCGATAACGGTCCAGCGTTCTTC**	21
**TGTGCCTGGAGTGGGCAGCCGAACACCGGGGAGCTGTTTTTT**	21
**TGTGCCACCAGCAACGGACAGGGGGTTCAAGAGACCCAGTACTTC**	21
**TGCAGTGCTCCGACTAGTTCAGGCGAGCAGTACTTC**	21
**TGTGCCAGCAGCTTAGCGGGAGGGGAGACCCAGTACTTC**	21
**TGCAGTGCTAGAGATCTCAGACCTGGGGCCAACGTCCTGACTTTC**	21
**TGCAGTGCTCCGGGAAGGAACACCGGGGAGCTGTTTTTT**	21
**TGTGCCAGCACCAAGGGCAGCTCCTACGAGCAGTACTTC**	21

**TGTGCCAGCAGCTTAGAGGCGGGGTTAGGTAATGAAAAACTGTTTTTT**	21
**TGTGCCAGCAGTTACGTCCGGACAGGGGGCTCTGGCTATGGCTACACCTTC**	21
**TGCAGTGCTGAATACAGGGACCAGGAAGCTTTCTTT**	21
**TGTGCCAGCAGCTTAGGTGGAAACACCATATATTTT**	21
**TGTGCCAGCAGTTACAACACAGGGGGGAGGACTGAAGCTTTCTTT**	21
**TGTGCCAGCAGCGTAGCGGGGACGCCTGAAGCTTTCTTT**	21
**TGTGCCTGGAGTGTAACGGGTAGGGACGGGGAGCTGTTTTTT**	20
**TGTGCCAGCAGCCTGGACAGGTACAGATCGCCAAACACTGAAGCTTTCTTT**	20
**TGTGCCAGCAGCCCTTATCAGGGTACAGATACGCAGTATTTT**	20
**TGCAGTGCTAGAGATCCCAGGACAGGGCAAAAGACCCAGTACTTC**	20
**TGCAGTACCAGCTCGAGCCAGAGCTCCTATAATTCACCCCTCCACTTT**	20
**TGCGCCAGCAGCTCCACCAATGAGCAGTTCTTC**	20
**TGCAGTGCTAGAGGGACTAGCGCTTTTCAAGAGACCCAGTACTTC**	20
**TGTGCCAGCGGCTACGACAGGGGCGTCTCCGAGCAGTACTTC**	20
**TGTGCCAGCAGCTTACGTTACTCGGCAGGGCTGGCCGGGGAGCTGTTTTTT**	20
**TGTGCCACCAGAGGAACAGGCAGCAACTCCTACGAGCAGTACTTC**	20
**TGTGCCAGCAGTTACGGCCGGCATAAGAACACTGAAGCTTTCTTT**	20
**TGCGCCAGCAGCTTGGTGGAGCCTCAAGAGACCCAGTACTTC**	20
**TGTGCCAGCAGTGGGACAGTTCTCAATGAGCAGTTCTTC**	20
**TGCAGTGGAGCCAAATCTGGGGCCAACGTCCTGACTTTC**	20
**TGTGCCAGCAGATTTGACAGTAGAAATTCACCCCTCCACTTT**	20
**TGTGCCAGCACCTATGGAGCAGGGACTGAGACCCAGTACTTC**	20
**TGTGCCAGCAGTTTCGAAGGGAACACTGAAGCTTTCTTT**	20
**TGCAGCGGGTCGACTAAACCCCTCTCAGATACGCAGTATTTT**	20
**TGCAGCGTTGAACCCGAAAGGATTTCAGATACGCAGTATTTT**	19
**TGCAGTGCTAGAGATAGGGGAGCGGAGAGCACAGATACGCAGTATTTT**	19
**TGTGCCAGCAGTTACTCGGATTTCAAGGGCAAGCAGTACTTC**	19
**TGTGCCAGCAGGATGACAGGGAAGAACACTGAAGCTTTCTTT**	19
**TGCAGTGCCGGAATAGGGGGTGAGCAGTTCTTC**	19
**TGCGCCAGCAGCCGTAGCGGGGCCTACAATGAGCAGTTCTTC**	19
**TGCAGTGCTGAAGACAGATCCTACAATGAGCAGTTCTTC**	19
**TGTGCCAGCAGTCCCTCGGACTGGGATGATACGCAGTATTTT**	19
**TGCAGTGCTAGTTTACAGGGACGAGCCGCCGAGCAGTACTTC**	19
**TGTGCCAGCAGCTTGAGGTTGTCTAGCGGGAGGTTTGTGGGGGATACGCAGTATTTT**	19
**TGTGCCTGGAGTGTGAGTGGGGCGGGAGAGACCCAGTACTTC**	19
**TGTGCCAGCACCCCGGGACAGAACACCGGGGAGCTGTTTTTT**	19
**TGTGCCAGCAGCGGACGGACAGGGGGAACCATATTCTACGAGCAGTACTTC**	19
**TGTGCCAGCAGCCCTGGGGACGGATCTAATGAAAAACTGTTTTTT**	19
**TGTGCCAGCAGCCATGACACGAAGCTTTCTTT**	19
**TGTGCCAGCAGCTTAATAGACAGCCTTAATTCACCCCTCCACTTT**	19
**TGTGCCAGCAGCTTCACGTCCGGGACAGGGAGTGGAAACACCATATATTTT**	19
**TGCGCCAGCAGCTTTACAGGGCAGACCTACAATGAGCAGTTCTTC**	19
**TGTGCCAGCAGCTCCATAGCGGGAGAGGGTAGGAATGAGCAGTTCTTC**	19
**TGTGCCAGCAGCTGGAGTCGGACCGGGGAGCTGTTTTTT**	18
**TGCAGTGCGGCGGGACAGTTGAACACTGAAGCTTTCTTT**	18

TGTGCCAGCAGCCCGCTCTCAGGGCTGGGCACCGGGGAGCTGTTTTTT	18
TGTGCCAGCAGCTCTCTTTCCGGGACAGGCGCGGGTTGGGCCAAAAACATTCAGTACTTC	18
TGTGCCAGCAGCTTAAGGGGGACAACCCAAGAGACCCAGTACTTC	18
TGCGCCAGCAGTGTAGGGGGCACTGAAGCTTTCTTT	18
TGCAGTGCTAGAGTTCCGACCACTAGCGGGAGGATCAACGAGCAGTACTTC	18
TGTGCCAGCAGCTTAGGGTTGGGCGGGAGGCACGGAGGGGGGCAGTTCTTC	18
TGCAGTGCTCTAAGCGGGAGAGGTCGTAATGAGCAGTTCTTC	18
TGTGCCAGCAGCCAAGGCCCACCCGGGATTTTTCAAGATTCACCCCTCCACTTT	18
TGTGCCAGCACCTTTCGCACCGGGGAGCTGTTTTTT	18
TGTGCCAGCAGCTTCACGGGGTTGAACACTGAAGCTTTCTTT	18
TGCAGTGCTAGAGATCAGGCAGCCACAATAGAAGAGACCCAGTACTTC	18
TGTGCCAGCAGCGCATCTGGGGCCAACGTCCTGACTTTC	18
TGTGCCAGCAGCTTCAGGGGAGGGCCCCACGAGCAGTACTTC	17
TGTGCCAGCAGCTTCCTGACTAGCGGGGCTTCCTACGAGCAGTACTTC	17
TGCAGCGTTGAGACAGGGGGCTCTGGAAACACCATATATTTT	17
TGCAGCGTTGATGGGACAGGGTCTGGCAATGAGCAGTTCTTC	17
TGTGCCAGCAGCTTAGAGGGGAATCGATCACCCCTCCACTTT	17
TGCGCCAGCAGCTTAGTAAGCGGACACAATGAGCAGTTCTTC	17
TGTGCCAGCAGCGCAGGGGGGGATGAGCAGTTCTTC	17
TGTGCCGTCTTAGCGGAGACCCAGTACTTC	17
TGCAGTCCCCGCGGAGCGGGAGGGCCCAGGGGCTACGAGCAGTACTTC	17
TGTGCCAGCAGTTACCGAAGCACAGATACGCAGTATTTT	17
TGCGCCAGCAGCTTCGTAAACATGAAGGCGAATCAGCCCCAGCATTTT	17
TGCAGTGCTAGAGGGGGACCCCCCTACAATGAGCAGTTCTTC	17
TGCAGTGCTGGACAGTCGAGCCGAGAGACCCAGTACTTC	17
TGTGCCAGCAGTTGGGACAGGGCTGGGTACACCGGGGAGCTGTTTTTT	17
TATGCCTGGAGTACCCGGACCTACCAGGGAGAATTC	17
TGTGCCAGCAGCCTAGCTCCACAAGAGACCCAGTACTTC	17
TGTGCCAGCAGCTTATACCGGAACACCGGGGAGCTGTTTTTT	17
TGCAGCGTTGAAGGGGGGAGGGCCCCCACAGATACGCAGTATTTT	17
TGCAGCGTTCTGGACAGTAATTCACCCCTCCACTTT	17
TGTGCCAGCAGCTCCGGACTGAGCACAGATACGCAGTATTTT	17
TGTGCCAGCAGTTTGTTGGGCTTGCCCCTCCACTTT	16
TGTGCCAGCAGTTACGTCACAGGGGTGGACGAGCAGTACTTC	16
TGTGCCAGCAGTTACTCGAGAGAAGCGATCTCTGGAAACACCATATATTTT	16
TGTGCCAGTCAACAGGGGGACACTGAAGCTTTCTTT	16
TGTGCCAGCAGTTACTCGAGGCAGGGGGGCTACGAGCAGTACTTC	16
TGTGCCAGCAGCGCCGTACAGGGGTGGTCAGGAAATGAGCAGTTCTTC	16
TGTGCCAGCAGCCCCGCCGGCGGGACTACCTACGAGCAGTACTTC	16
TGTGCCAGCAGACCTAGAGTAAGCGGGAGGAGGACGAACACCGGGGAGCTGTTTTTT	16
TGTGCCAGCAGTGATAGGGGCTATGAGCAGTTCTTC	16
TGTGCCTGGAGTGTACGGTCGACAGTCTATAATTCACCCCTCCACTTT	16
TGCAGTGCCGGGGGGGCAGACTACGAGCAGTACTTC	15
TGTGCCAGCAGCCAAGCGCCAAAAGAGACCCAGTACTTC	15
TGTGCCAGCAGCGCTACTAGCGAACCATCCTACAATGAGCAGTTCTTC	15

TGCAGTGCGACACCCGAAACGGACCACGACTACGAGCAGTACTTC	15
TGTGCCAGCGCAGGCATGAGCTCCTACGAGCAGTACTTC	15
TGTGCCAGCAGAACGAACTTGGGCACTGAAGCTTTCTTT	15
TGCAGTGCTAGAAGGACAGGGGATCACACAGATACGCAGTATTTT	15
TGTGCCAGCGGCAGGGAGTACGAGCAGTACTTC	15
TGCAGTGCTAGGCAGGGGGGGACAGAGGGGCCCGAGCAGTACTTC	15
TGTGCCAGCAGCCTCCGACTCCCGGGGGATGAGCAGTTCTTC	15
TGTGCCTGGAGGGGCAGGGGGCCTGGAAACACCATATATTTT	15
TGCAGTGCCGCCCTGGAGCTAGCGGCCACAGATACGCAGTATTTT	15
TGCAGTGCTAGCGACAACTATGGCTACACCTTC	15
TGTGCCAGCAGCGACGGGGGTGCGCGGGATCGTACGCAGTATTTT	15
TGTGCCTGGAGTGCTGGGGGCCGAGAGACCCAGTACTTC	15
TGCAGCGCCCCCGGGACAGGGAGAACAGATACGCAGTATTTT	15
TGTGCCAGCAGCTTAACGGGGGGCGCAGAGACCCAGTACTTC	15
TGCAGTGCTAGAGATCGAGCGGGAAGGAACGAGCAGTACTTC	15
TGTGCCTGGACACCAGGAGCGCTTATTGAAGCTTTCTTT	15
TGTGCCAGCAGTTTTCGAGTTGCCTATGGCTACACCTTC	15
TGTGCCAGCAGTTACAGTCGCGTTTTTGGGGATGAGCAGTTCTTC	15
TGCAGTGCTCCTAGGACCCTGGGGGGGGAGCAGTACTTC	15
TGCGCCAGCAGCCAAGGCCTCCGGGGCAATGAGCAGTTCTTC	15
TGTGCCAGCAGTTACGGACTTGGGGGGGACACTGAAGCTTTCTTT	15
TGTGCCAGCAGCTTCCAGCTAGCGTCTTCCTACAATGAGCAGTTCTTC	15
TGTGCCAGCAGCTTAGGTGCGATGTACGAGCAGTACTTC	15
TGTGCCAGCAAAAGGACAGGGGGCTCCTACGAGCAGTACTTC	15
TGTGCCACCAGTTTGAGGGAGAACACTGAAGCTTTCTTT	15
TGCGCCAGCAGCTTCAGACCGAACCCCAACACAGATACGCAGTATTTT	15
TGCGCCAGCAGCTTGGCCGCCCAGGGGATGGACACTGAAGCTTTCTTT	15
TGTGCCACCAGCAGAGATCGTCAACCCGGTAGGGGGACAGCGAACACCGGGGAGCTGTT	15
TGCAGTGCTAGAGATATGAGCGGTTCTGTTAATACGCAGTATTTT	14
TGTGCCAGCAGCTTCCTCCCGACTAGCGGGAGGTACGAGCAGTACTTC	14
TGCAGTGCTAGGACTAGCAATGAGCAGTTCTTC	14
TGCAGTGCTAGTAAGTCGCAGGGATCAGCCCAGCATTTT	14
TGTGCCACCAGCAGAGCCCTAGCGGGAGGGCAAGAGACCCAGTACTTC	14
TGTGCCAGCAGGTCCGGGACTAGCGGCGGTCCCCACGAGCAGTACTTC	14
TGTGCCACCAGCAGAGACACCCCCGCCGGACAGGGTGGTTCCTACGAGCAGTACTTC	14
TGTGCCAGCAGCTTATTGGGGGGCTCAGAGACCCAGTACTTC	14
TGCAGTGCTAGAGATGAGGGAGTACGGCTGTTTTTT	14
TGTGCCAGCAGCTCCTTAAGGCCCGGGCTGAGCGACGAGCAGTACTTC	14
TGTGCCAGCAGCCAGGTGCGGACTGAGGCTTTCTTT	14
TGCGCCAGCAACAAAGAATCACAGGGCTACGAGCAGTACTTC	14
TGTGCCAGCGGACAGGGGGCGAGCACAGATACGCAGTATTTT	14
TGTGCCAGCAGCATACAGGGGGCCGCCCTCTATGGCTACACCTTC	14
TGTGCCAGCAGTTACTCGTGCGGGGAATCGTTGAAGCTTTCTTT	14
TGCGCCAGCAGCCCGGGGACAGGGGTTTACGAAGATACGCAGTATTTT	14
TGTGCCTGGAGTGTACCTGGGGGCAATGAGCAGTTCTTC	14

TGCGCCAGCAGCCCCTCCACGTCTAGCGGGAAGATAAATGAGCAGTTCTTC	14
TGTGCCAGCAGTTCCCAAGTCTACGAGCAGTACTTC	14
TGCGCCAGCAGCCCCGAGGGGCCCACTGAAGCTTTCTTT	14
TGCAGTGCAGGGCTAGCGGGAGCAGATACGCAGTATTTT	14
TGCAGCGTTGAAGATCGGCAGGGGAACACTGAAGCTTTCTTT	14
TGCAGTGCCCAACAGCCGGGGGGTGAAGCTTTCTTT	14
TGTGCCAGCAGCTTTCCGGGACATAAATACGAGCAGTACTTC	14
TGCAGTGCGCTCCCAGGACTTGGGTCGGATGAGCAGTTCTTC	13
TGCAGCCTCCGGGGGGATTACGGCACAGATACGCAGTATTTT	13
TGCAGCGGTCTACAGGGCAGGGAAACTGAAGCTTTCTTT	13
TGTGCCAGCAGCTCGGGGGGTGAGCAGTTCTTC	13
TGTGCCTGGAGTACCCGGACAGGGGGCAATCGTGCAACTAATGAAAAACTGTTTTTT	13
TGCAGCGTCTCCGATGCCTACGAGCAGTACTTC	13
TGCAGTGCGATTAGGACAGGGGAGAATGGCTACACCTTC	13
TGCAGCGTTGGGGTCGGCCGAACTGGCTACACCTTC	13
TGCGCCAGCAGCCCCGGACAGAGAAAGGATACGCAGTATTTT	13
TGCAGTGCCGGACTAGCGGGAGCAGATACGCAGTATTTT	13
TGCGCCAGCAGCTTGCGGGGTCAAGCGAACACCGGGGAGCTGTTTTTT	13
TGTGCCAGCAGCTTCCTCAGAGATGCCTACACCTTC	13
TGCGCCAGCAGCTTGGCCAGGGTAGGGGTGAACAATGAGCAGTTCTTC	13
TGTGCCAGCAGTTCCCAGACTAGCAACTCCTACGAGCAGTACTTC	13
TGCGCCAGCAGCTGGTGGGGCTCCTACAATGAGCAGTTCTTC	13
TGCGCCAGCAGCTTGGTTGGCAGCAGCTCTGGGGCCAACGTCCTGACTTTC	13
TGTGCCACCAGCAGGCAGTCCCAGGGACTAGCGGGGACAGATACGCAGTATTTT	13
TGTGCCAGCAGCTTAGGGTCTGGATTTGCGCAGACCCAGTACTTC	13
TGTGCCAGGAAGTCCTACGAGCAGTACTTC	13
TGTGCCACCAGCAGAGGACAGGGGGCGGCCGATGGCTACACCTTC	13
TGCGCCAGCAGTTCCGAGGGGGGAATTGAAGCTTTCTTT	13
TGTGCCAGCAGCCAATTCCAGCTCATGAACACTGAAGCTTTCTTT	13
TGCAGTGCTAGAGCGGGGGCCGGGAATCAGCCCCAGCATTTT	13
TGTGCCACCAGCAGAGGGGGGGACGGGGCAGATACGCAGTATTTT	13
TGCAGTGCTAGCGGGACTAGCACAGATACGCAGTATTTT	13
TGCAGTGCTATAGCAGCTGGGGCCAACGTCCTGACTTTC	13
TGTGCCAGCAGACGGACAGTGGATGGGGCCAACGTCCTGACTTTC	13
TGCAGTGCTCCCGCCGGGGGCATCTATGGCTACACCTTC	13
TGTGCCAGCAGCTTAGAAGGGTCCGGGGAGCTGTTTTTT	12
TGCAGTGCTAGGAGCACTAGCGGGACCCACGAGCAGTACTTC	12
TGCGCCAGCAGCCCACTAGCGGGAGTGAATAATGAGCAGTTCTTC	12
TGTGCCAGCCTGCGGGGGGGCACCGGGGAGCTGTTTTTT	12
TGCGCCAGCAGCTTGGAGGGGCACGCGACCCATGAGCAGTTCTTC	12
TGTGCCAGCAGCACGCAATGGGGCAGGGGTTTTTCCTACGAGCAGTACTTC	12
TGTGCCAGCAGCGTGTTTGGCTGGGGGCCACAAGAGACCCAGTACTTC	12
TGTGCCAGCAGCTTAGCGGAGGTCAGACTCTCTGGAAACACCATATATTTT	12
TGTGCCAGCAGCCTTGGGTCACAGCGGGGTCCGCAGTATTTT	12
TGTGCCAGCAGCTTAGCGGGAGGGCCCTACAATGAGCAGTTCTTC	12

TGTGCCAGCAGTTACGGGAATTCCCTGAACACTGAAGCTTTCTTT	12
TGTGCCAGCAGCCAAGTGGCGGGGGGCGGGACTGAAGCTTTCTTT	12
TGTGCCACCAGCAGAGATCGGGAACAAACCGAGCAGTTCTTC	12
TGTGCCAGCAGCTCTCCCGTGGGACAGCTCCTACGAGCAGTACTTC	12
TGTGCCACCAGCAGAGAGGCGTCTAGCGGGCAATACGAGCAGTACTTC	12
TGTGCCAGCAGCTTATCCGTGGGCTTGGCTAGCGGGATAACCTACGAGCAGTACTTC	12
TGCAGTGCTAGAGAGGGCGGGCTTACCTACGAGCAGTACTTC	12
TGCAGTGCTAGAAGGGGGACCCCCTCGGCTTTCTTT	12
TGTGCCAGCAGTCCCCCCGGGACAGATACGCAGTATTTT	12
TGTGCCAGCAGTCATGGACACCATGGTGGAGAGACCCAGTACTTC	12
TGCAGTGCTAGAGATCAGGTCCAAGAGACCCAGTACTTC	12
TGTGCCAGCAGCCCGACAGGGGGCTCTGGCTACACCTTC	12
TGTGCCAGCAGTCGGGACCTTGACTCCTACGAGCAGTACTTC	12
TGCAGTGGTGCTAGAATTGCTGAAGCTTTCTTT	12
TGCGCCAGCAGAATCACAGGCGGATACGAGCAGTACTTC	12
TGTGCCAGCAGCCCCGGACTAGCGGGAGATCACGAGCAGTACTTC	12
TGCAGCGTTGAATCGGGCCGGGGGGTCCTGCCGCAGTACTTC	12
TGCAGTGCGGGACGGACAGGGGGCGGTGAAGCTTTCTTT	12
TGCGCCAGCAGTGAAACTAACTATGGCTACACCTTC	12
TGTGCCAGCAGTAGCCCGGGACAGCCCTACGAGCAGTACTTC	12
TGCAGTGCTGAATTCGGGACAGCTAATGAAAAACTGTTTTTT	12
TGTGCCAGCAGCCGGACAGGGGGCTCCTACAATGAGCAGTTCTTC	12
TGCAGCGGTCGGACTAGCGCCCCAGATACGCAGTATTTT	11
TGTGCCAGCAGCGTAGGAGGGACAGCGAACACTGAAGCTTTCTTT	11
TGCAGTGCGTTGGACAGGCCCTTATACAATGAGCAGTTCTTC	11
TGCGCCAGCAGCTTGGCTCAGGTCGGGGACTATGGCTACACCTTC	11
TGTGCCAGCAGCGCGAGGGGCTCTGGAAACACCATATATTTT	11
TGTGCCAGCAGCCCTAGCGGTTCCTACAATGAGCAGTTCTTC	11
TGTGCCAGCAGCTCGTGACTGATCAGCCCCAGCATTTT	11
TGTGCCAGCAGCTGGACAGGGGCGGGAAAACTGTTTTTT	11
TGTGCCAGCAGCCCCGGGACGCCTGGCAATCAGCCCCAGCATTTT	11
TGTGCCAGCAGCTTACTCTATGGGACTTCAGATACGCAGTATTTT	11
TGTGCCAGCAGCAGCCGAGGTGGGAGCTCAGCTACGCAGTATTTT	11
TGTGCCAGCAGCTTAGCGCGCAGCGCTAATGAAAAACTGTTTTTT	11
TGCGCCAGCAGGGAGGGGTCTGACTACGAGCAGTACTTC	11
TGTGCCAGCAGCTTATTCCTCTCTGGGGCCAACGTCCTGACTTTC	11
TGCGCCAGCAGCTTCCGGGACAGCACAGATACGCAGTATTTT	11
TGCAGTGCTACCCTCCCGGAGCGGGATTTCTACGAGCAGTACTTC	11
TGTGCCAGCAGCGTCCGAGGAGGAAGGACTTTCTTT	11
TGCGCCAGCAGCCAAGAAGAGCGGGAAGAATGAGCAGTTCTTC	11
TGTGCCAGCAGTCCCCTGAGGGCCTTTAGTGCGGGAGCTGGAGAGCAGTTCTTC	11
TGTGCCAGCAGTTACTCGGACGGGAATAATTCACCCCTCCACTTT	11
TGTGCCAGCAGTTACTCGGCAGGGGGCTCCTACAATGAGCAGTTCTTC	11
TGTGCCAGCAGTTACTCTCTACGGCTAGCGGGAGAAAGCGAGCAGTACTTC	11
TGCAGTGCTAGTAGGGGACAGGGTTTCTACGAGCAGTACTTC	11

**TGTGCCAGCGCACAGGGACTAGAGAATGAGCAGTTCTTC**	11
**TGCAGTGCTAGAAGGGACTCGAACACCGGGGAGCTGTTTTTT**	11
**TGCAGTGCTACTGGGGAAAAGAGTTGGAATTCACCCCTCCACTTT**	11
**TGTGCCAGCAGCTTTCGCGGGAGGAGCACAGATACGCAGTATTTT**	11

CDR3 AA Seq
CASSFRTSGEQYF
CASSPPRGVRELFF
CAWSVWGTRNTEAFF
CAWQKQDNYGYTF
CASSQEPGHNQPQHF
CAWSVDSGNQPQHF
CAWSVLAAGQETQYF
CASNKAANQETQYF
CAWSVRDREVIEAFF
CAWSKTGGSEQFF
CASSQLGQYTGELFF
CASSLGTEPSYEQYF
CAWSTRTYQGEF
CASSLASLAGEQYF
CASSPGTVTDTQYF
CAWSVGPGLNTEAFF
CAWTRLGNTIYF
CAWRPRTSGELFF
CAWATGENQPQHF
CAWSVRGETQYF
CASSSGASPQETQYF
CAWSVVLAGLETQYF
CASSPLLNTEAFF
CASSSSNEKLFF
CASSQIGETQYF
CASSRPATNTEAFF
CACRGGQGNYGYTF
CAWIAAGRETQYF
CAWSGVSNTEAFF
CASSPRPREQFF
CASSEGTGNYGYTF
CAWRVAGGLLEQYF
CASSPPDSGLPNQPQHF
CAWSVLQETQYF
CASSLNRGSYEQYF
CAWSSGLAGGELFF
CSVERGSNEQFF
CAWRGDRYYEQYF
CAWSVGGGGDTQYF
CASSSRLASEQYF
CASGTSGGTDTQYF
CAWRPTGARDTQYF
CAWMVSYGYTF

CASSQAESNQPQHF
CASSARLAEETQYF
CAWRDYRDRGNTF
CASSFGVWNSPLHF
CASSLGLAEDTQYF
CASSLAGGLTYEQYF
CASSLPSPDEQYF
CASSLLPGMNTEAFF
CASSFRGLRTDTQYF
CAWRAPGTFHEQYF
CASSLDGTGDTEAFF
CASSLSIDTGELFF
CASSAAGFSYGYTF
CASSVDYQETQYF
CASSLQSGAKAGELFF
CASSLLSGANVLTF
CASSQVAGTSSGANVLTF
CASSQDRGTRDQPQHF
CASSLPFSGSEQFF
CAWSVPPSYNEQFF
CASSLVFGRAYNEQFF
CASSIGSGPQFF
CASSFSGSYNEQFF
CASSSPNGPVNTEAFF
CAWSIPGAGQPQHF
CAWSRSQGASQPQHF
CASSLTFPPETQYF
CASSRIGVGDTQYF
CAWSPLAGGNTEAFF
CASSSGFSYEQYF
CASSQDGQGRGEQFF
CASSLVGSSPQETQYF
CASSGDSLYGYTF
CASSLIARLIDGYTF
CSVEASLGEQFF
CASSLGGGFSTDTQYF
CASSSPTAGMNTEAFF
CASSLTVLTEAFF
CASSTGTNTGELFF
CAWSGVRRNEQFF
CAPLSPGSTEAFF
CASRSREGHSNQPQHF
CASSLGPLWNTGELFF
CATSRAHTSYGYTF
CASSLGSGLPYF

CAWSVLQGGYTF
CATSRDLGSGGETQYF
CASSLGGYSYNEQFF
CASSLRTGGGYEKLFF
CASSLASRSYEQYF
CASSSDLNTEAFF
CASSLGSGNNEQFF
CASSQDTSRSEAFF
CASSLDSNEQYF
CASSPVSSRDLSYEQYF
CAWKGWQGLYGYTF
CASSLFTDTQYF
CAGTGSMGTYGYTF
CATSRDRGVTEAFF
CASDTGDPSNYGYTF
CASSNPTGGTDTQYF
CASSYSWTSGSGELFF
CASSSTGGQSPLHF
CASSLGNRRQETQYF
CASSRDREGGYTF
CASLRGQGNQPQHF
CAWSVLSGVQPQHF
CASSLDSAEAFF
CASSLGPLAGAPYNEQFF
CASSLETSSRETQYF
CASTRDRVDEQYF
CAWAPSGEGDNQPQHF
CASSQDRGANYGYTF
CASSPSTSVDEQFF
CAWSVLAGVYGYTF
CAWSVIGGNYGYTF
CASSLDRGVQYF
CASSPGAGLGNYGYTF
CASSWSAATDTQYF
CAWSQGWTEAFF
CASSLATTGRDIQYF
CASSQAGTPDTQYF
CASSSGGQGMDTQYF
CASSPSGTVGEQYF
CASSLASQGTRSDTQYF
CASSLGSVGETQYF
CASSHVFRIGSRGTGDTQYF
CASSFGQGGTDTQYF
CASSLDRGTEAFF
CASSTSSYNEQFF

CASSYDEKLFF
CASTSGGGLDTQYF
CASSFPGTSGGYTF
CASSLGAYEQYF
CAWSRRSQETQYF
CASSSRQGGDTEAFF
CAWSAERDSHGYTF
CAWSGQANSGANVLTF
CASSQGTGRRPPYQETQYF
CASSPLAEAFF
CASSLFTGTGELFF
CASSQERDQPQHF
CASSQDSNQPQHF
CASSQTGPSDYGYTF
CASSRRLATNEKLFF
CSARPVVNQPQHF
CASSLDREETQYF
CATSRGGDGYGYTF
CSVELPSQGGETQYF
CAWSDPGGGSYNEQFF
CASSQDRGNQPQHF
CASSQDVPGSSGNTIYF
CAWGGLPGADTQYF
CSASAGTSVAYEQYF
CASSLVGPGSGLGDEQYF
CASSVAGGGQPQHF
CAWSVQGGGELFF
CASSLVHLRNEQFF
CASSQLGAGEQYF
CAWSGRNEKLFF
CASSSGRTGETQYF
CASSLGGQGGNTEAFF
CSVRPSGSHEQYF
CASSGGGSRSNQPQHF
CASSLAGKTQYF
CASSPDRGGSYGYTF
CASSSPAGQEALSNYGYTF
CASSSDFYPYEQYF
CAWTDRRGGPNGQFF
CSVEVYSSNEQFF
CAWSVGSNSNQPQHF
CASRPGTGGDTIYF
CAWSVGTKETQYF
CAWRGRGGLNTEAFF
CASSPNQGSGANVLTF

CASSAGGDYGYTF
CASSQDLGGGIQYF
CASSPRGGRTFNQPQHF
CASSYDRQGEQYF
CASNRDGSYGYTF
CASSVTRVQSNYGYTF
CASSPLVGDQPQHF
CASSRLAAKNIQYF
CASSLDGIREQYF
CASSFNSGQGYEQYF
CAWKPGLASNTGELFF
CASSLAPAGGGTDTQYF
CASRRLQGPSYGYTF
CAWTLSGGNTIYF
CASSSETGSGNTIYF
CASSLTRQSTDTQYF
CASSLDGTGDTEAFF
CASSQDKDTQYF
CASSFSTDTQYF
CASSLDLKTGYTF
CASSPGTSGVNEQFF
CASSPTSGGDTQYF
CASSLVGAGSGDPTQYF
CAWSVGEQETQYF
CASSNGRRGGVGPRRETQYF
CASSYGGAQTEAFF
CSVGQGAEAFF
CASSSNLPGTTNTGELFF
CSVEDQAGVFGTEAFF
CSAAAPGGWETQYF
CASRQGINTEAFF
CASSDPQLTQYF
CASSPRTSGNNEQFF
CAWSVDNYGYTF
CSASTGGQLGYTF
CASSQDASVWASNQPQHF
CASSSGRAQPQHF
CSVTGNSYNEQFF
CASSPGVADTQYF
CATPRGDSANTGELFF
CASRGGQNTEAFF
CASSSAEYNEQFF
CASSLGWISNQPQHF
CSARVPGDRDGEQFF
CASSQAQVVESIQYF

CATGVGQETQYF
CASSLPPTDTQYF
CASSLDAGESYEQYF
CASSRTDSQETQYF
CASSARVDIQYF
CASSPTHTTDTQYF
CASSLDGAGGGTNGYTF
CASSQGLAGWDEQFF
CASSQVIGGRRPEAFF
CASSPILNTGELFF
CATSRGRTEPLHF
CASSFGRYNEQFF
CASSLAYSGGLETQYF
CASSLEGQGPYEQYF
CASSLSTDTQYF
CASSLPPWTAGYTF
CASSFGRYDEQYF
CAWAIRGALNYNEQFF
CASSLGGSTYEQYF
CASSQTGGGSEAFF
CASSFRRAASTDTQYF
CAWSVEGGGLRGSQPQHF
CASSLVKGADQPQHF
CASSQWGTETTSNQPQHF
CSASLSWGGQNTGELFF
CASSLPRRTDNSPLHF
CASRRDVHEQFF
CASSQGTSYNEQFF
CSVEHSYEQYF
CASTPRTGADTQYF
CASSIGTSGSGWETQYF
CAWSFPPRGQGGNQPQHF
CASSPASGSLEQFF
CASSSGWEGGTEAFF
CASSQGPGGRSYNEQFF
CAWSRGQGTYEQYF
CASSLVGGGTSYQETQYF
CASSRDRSSYEQYF
CSARVYNEQFF
CSAGGLAGGLGNNEQFF
CASSQDDRVGGSQFYNEQFF
CASSFQRGAYNEQFF
CASSQSWQSTDTQYF
CASSQDSRVLGGYTF
CSVGERGTQYF

CAWSRGGVGGELFF
CASSPVGGEETQYF
CASSLAGTGSYYGYTF
CASSLYSGRETQYF
CASSPGQGASGELFF
CASSLNPGGAGYTF
CASSQDTSRGETQYF
CASSLVDFYEQYF
CASSSVNQPQHF
CASSRGLANNEQFF
CSARDSSIGPNQPQHF
CASSLIGFSYF
CASSPQTGGYGYTF
CASSDLREDYGYTF
CSASPPIAGGAQYF
CASRRRQAGQAFF
CASSLGGFNQPQHF
CASSFDRYNEQFF
CSVGQGPFQPQHF
CSTSGGGTDTQYF
CSVEPQGQASRETQYF
CASSLGRAGGPNEQFF
CASSTHLDTGELFF
CASSPRGGRAADTQYF
CSVADSFPANIQYF
CASSQGLTWQETQYF
CASSIPTGGAGNTQYF
CASSQEGLAGGGYNEQFF
CSEAGGNTEAFF
CSVSGQAGNQPQHF
CASSFGREETQYF
CATSGRQGWDQPQHF
CASSLRQGAGELFF
CASSPGPGAHSYEQYF
CATSRPSQGSPVEQYF
CASSLPTDFYEQYF
CASSGGQAETQYF
CASSLVSGQYPTYEQYF
CASSWTGGVYEQYF
CASSFLQEQFF
CASSQDRGGTYEQYF
CASSLTGGIYEQYF
CAFRDTSYEQYF
CASSQDRKPAGANVLTF
CASSLVQGVEKLFF

CSVEDGGHEQFF
CAWTAPTVGSAGELFF
CASSLGPGGLFSGANVLTF
CASSTYPPGQGAREQFF
CAWSRDRQGANVLTF
CASSLSGADINEQFF
CAWSVKGAGETQYF
CASSLAGSNSPLHF
CASSPGTGVEQYF
CASSSGVSTDTQYF
CAWSDRGPGGSPLHF
CAWTRGNTKEQYF
CASSLVLAGGSYEQFF
CASSTNTGGQHF
CASSSTSSTDTQYF
CASSPGQGNNEQFF
CASSQGAGLYTDTQYF
CATSRGGGTEAFF
CASSFRLDNEQFF
CASSPRRAGGDNEQFF
CSARSWTGQGNQPQHF
CSASTGGADGYTF
CAWRKAGGIYNEQFF
CASSQGDQAYNEQFF
CASTAVAGGYNEQFF
CASTLGQGWTEAFF
CASSLYGDGRSTDTQYF
CSGSTLANNEQFF
CASSGPIPYEQYF
CAWGATGTSGLVV
CSADAQGSGELFF
CASSPQTGGGSYNEQFF
CASSVRSSKFF
CASSLEISKTDTQYF
CSAKDPLRDTQYF
CSVEVGGGVRFF
CASSQEARGLDTGELFF
CASSSGLANTGELFF
CASSPGLARTYRDEQFF
CASSQAHPGTGQNSPLHF
CASSFGAPVGEAFF
CASRPGTAREAAFF
CASSRGVQETQYF
CSASTQGASGYTF
CAWSTLNEQFF

CASSPRQGNTGELFF
CASSQDPSGRTISYEQYF
CASSSLGTGSDEQFF
CASSLVLAGAEQYF
CASSLLASGGNEQFF
CASTPEGGNTEAFF
CASSPRPTQGKGYTF
CASSHQWGEQYF
CASSLLSNYEQYF
CASRRDGYEQYF
CSADGLANYEQYF
CSASPTGVGGANVLTF
CAGGPSSINTGELFF
CSAAVLSNQPQHF
CASSLAGTSETDTQYF
CASSIGLARDTQYF
CASSPYGGNQPQHF
CAWENSGANVLTF
CSANPTGESNQPQHF
CSVEDPPGADTQYF
CASSYAGDSYEQYF
CSVKSPLAGGPLFSTDTQYF
CASRAGTDMNTEAFF
CASSLEGPGQGRSDTQYF
CASSLARNQPQHF
CASSLNGGTALEQFF
CASSLQPGTFNEQFF
CASSTGLRAWETQYF
CASSFGVSYEQYF
CSVARQDQQFF
CASSLRRVPGLAGGGDEQFF
CASSSPGQPNGYTF
CASSLARVREQYF
CSATDPDSEQFF
CASTLTGSRSEAFF
CSARVAGGGQETQYF
CASSYGGPGTAYEQYF
CASSRGQYDEQYF
CASSLEGIGGRNQPQHF
CSVKTGGARQETQYF
CSVDSTDTQYF
CASTVGASGSSTDTQYF
CSVLRDPNSYEQYF
CASSQGASGRAYEQYF
CASSIAGEYEQYF

CSAPEQNTEAFF
CASSEAADTQYF
CASSQDSSIGGELFF
CASPLGGRNPGELFF
CASSPIRQPQHF
CAWMGEDNSPLHF
CASSSAGGDGVKLFF
CSVDLFDTQYF
CASRVDRNYGYTF
CASSAWTGSNLNEQFF
CASSLGTANTGELFF
CASSLEGRYNEQYF
CASSLAIGETQYF
CASSLVRQPLGAFF
CSARFKPEQETQYF
CASSQDYRSIYGYTF
CSASNKDSRYEQYF
CASSLGAVPEAFF
CASSQELTHEQFF
CASSYSMLPGTGNSPLHF
CSVEDRGQGGGGYTF
CSVEETGTGTYEQYF
CSAITAGGSDTQYF
CSASPGLALDTQYF
CASSGSFSSYEQYF
CASSLALGGNGNTIYF
CASSSYSGKNTGELFF
CSASGKGTLLTQYF
CASSPRQASYGTEAFF
CSARDGAGKGYTF
CATSMGGTAKNIQYF
CSARVPGQAYSNQPQHF
CSAPPQGETQYF
CASSWSGQGNNSPLHF
CSFRDLANTGELFF
CASGSGSNTGELFF
CSVVRDMGRDEQYF
CAWYPGQANTEAFF
CASSPGLASGELFF
CASKVRGNTEAFF
CASSLANGYEQYF
CASISSGNTIYF
CASSFSPASTDTQYF
CSARDRVATETQYF
CAWSRGREGPYEQYF

CASSMTSGSGDTQYF
CASSQGLGPATQYF
CASSLELASYNEQFF
CSGDGMNTEAFF
CSGVPRNTQYF
CASSQEDGGRPTYEQYF
CASSQYETQYF
CASSQDASGTGGKNEQFF
CASSLGTQETQYF
CATSLVLSYTEAFF
CASSLAYRGGSSSGNTIYF
CASSLTGGETQYF
CASSTRTGGDQPQHF
CSVDAGTGGVTEAFF
CATSPLAGGPTGELFF
CASSVAADASYEQYF
CASSLAGLRGDEQFF
CSASFGQVFTDTQYF
CASSLARGRSEQFF
CASRPIGQGYEQYF
CASSSRGSGSSGRPDTQYF
CAWHRTATMYNEQFF
CASSQDRVRVAPDTQYF
CASSSGSEAFF
CASSYRTGSSTEAFF
CAWSVRERETQYF
CAWSPLREQYF
CASSPGTGVNQPQHF
CASSLNPGGTYEQYF
RASSFPL_NQPQHF
CASSPGTAKNTEAFF
CSATGTGIETQYF
CASSPVGQGFFSGANVLTF
CASSLYGLARETQYF
CASSQDPGQGNEKLFF
CASRMVPSGNTEAFF
CSWDTEAFF
CASSEVPQGPQHF
CASSPQQTQYF
CASSQGVGAGDNEQFF
CAWSLRGDSPLHF
CASSRITESELFF
CASSQEARGGTDTQYF
CSVEDRDRGNLYEQYF
CASSNSGHYEQYF

CASSLVQGSDRPYEQYF
CSVLRDYEGETQYF
CASSLVASGSNEQYF
CASSLYGGGRETEAFF
CATSSTGLQETQYF
CSPSGSSYNEQFF
CASSPTDEKLFF
CASSLADYTDTQYF
CASRMRLAGGADTQYF
CSATYRDRGPGEQYF
CASSRMTGVQETQYF
CASSTGGSNYGYTF
CSVEQGALGTDTQYF
CSAIRDSGNYGYTF
CASSLEVLTF
CASSFYRDRDYGYTF
CSARDPGGTEAFF
CASSFLGYEQYF
CASSQETSAFF
CASSLNNYEYTF
CASSQGRTGGHYGYTF
CASTSLGGGLNTEAFF
CASSPGQALGELFF
CASSQDWGGESPLHF
CASSSTNSDTQYF
CASSKGLATSGELFF
CASSSLQAGGRNEQFF
CASSPQGPYGYTF
CASSILGGTGSYEQYF
CASSILPRGFLANNEQFF
CSASDEGRSYEQYF
CASSLGYRGGEQETQYF
CASSYRSSTDTQYF
CASSLRGDRDTEAFF
CASSLAFGQGAGGYTF
CSASTSVPPEETQYF
CSVQRLAGEEDNEQFF
CASSLGISREGGYTF
CASSQARGETQYF
CSVAQGSDTQYF
CASSLSMGQETQYF
CAWGGAGNQPQHF
CASSYQMTGYGYTF
CSVARQGAREQYF
CSAIPRDTQYF

CATSRGGSRQETQYF
CSVQEYNEQFF
CSVRPTSGGADTQYF
CASSQGPWDRVEYGYTF
CSAMGVPGQGAVEQFF
CASSLTSGIHNEQFF
CASSLAEGDAPGQYF
CSARRTSVYEQYF
CASSLDGGDRNEQFF
CASSPPTRFNEQFF
CATSARQGTEQYF
CASSPGQGGYGYTF
CASSFADTQYF
CASSLTGGTNEKLFF
CASRPGTGGSYEQYF
CAWSAMEDSPLHF
CSASGGQGFSTDTQYF
CASSPNIGDSGANVLTF
CASSPTDETQYF
CASSYTSGRLDTQYF
CASSPFMGNTEAFF
CSAGQSSNQPQHF
CASSYSRTNQPQHF
CASSLGGTLNGYTF
CASTLGGLVTYNEQFF
CSAPLRLAGGYTDTQYF
CASSLGDTTREPQHF
CASSLVRAGGRETQYF
CATSKGQNTDTQYF
CASSVGLAVRDEQFF
CASSLTASGRALF
CASSPWTGGTEKLFF
CAWGGRRSEQYF
CASIYGTASYEQYF
CASTLTGQVSSYEQYF
CASSLSRSATGELFF
CASSLPGYNEQFF
CASSRRLAGSADSSYEQYF
CASSPTTGGRTDTQYF
CASMGGTAYEQYF
CSASAPGYNEQFF
CASSEQGSGKNIQYF
CSVCPGLAATDTQYF
CASSYGASVGELFF
CASSLVSSNNYGYTF

CASSFLAGGFADTQYF
CAWSGIRRNNEQFF
CASSLWDGANTGELFF
CSVEVSYEQYF
CASRQAGSSYNEQFF
CASSQDWGLAGGNEQFF
CATSRDGSYGYTF
CSASDPLGETQYF
CASSLSQVMNTEAFF
CASSYGLAGKNIQYF
CASSSGQALNYEKLFF
CASSVDRGAMGNEQFF
CASRGQGAVALHF
CSGTSGADEQFF
CASSDTSDAQETQYF
CASSLVAALRDTQYF
CATLREQNTEAFF
CASSRSVDNEQFF
CASTRVSVNTEAFF
CSARSDSTNTEAFF
CAWSRLAGVYEQYF
CSVGVGGDTEAFF
CATTFSSGRGASTDTQYF
CASSQVSLSRDRNTEAFF
CASIPRGGYGSPLHF
CASSQAGNYEQYF
CAWGQGAGKSSETQYF
CSVEYSGDQPQHF
CASSQGRYSEAFF
CSASGRVEWAFF
CASKDSHTEAFF
CASSLEQGRGNTEAFF
CSAEGRGETEAFF
CASSLQPGQGYEQYF
CASSAGQAASDTQYF
CASSQDEGKTQYF
CASSPFGGREETQYF
CASSDNGPAFF
CAWSGQPNTGELFF
CATSNGQGVQETQYF
CSAPTSSGEQYF
CASSLAGGETQYF
CSARDLRPGANVLTF
CSAPGRNTGELFF
CASTKGSSYEQYF

CASSLEAGLGNEKLFF
CASSYVRTGGSGYGYTF
CSAEYRDQEAFF
CASSLGGNTIYF
CASSYNTGGRTEAFF
CASSVAGTPEAFF
CAWSVTGRDGELFF
CASSLDRYRSPNTEAFF
CASSPYQGTDTQYF
CSARDPRTGQKTQYF
CSTSSSQSSYNSPLHF
CASSSTNEQFF
CSARGTSAFQETQYF
CASGYDRGVSEQYF
CASSLRYSAGLAGELFF
CATRGTGSNSYEQYF
CASSYGRHKNTEAFF
CASSLVEPQETQYF
CASSGTVLNEQFF
CSGAKSGANVLTF
CASRFDSRNSPLHF
CASTYGAGTETQYF
CASSFEGNTEAFF
CSGSTKPLSDTQYF
CSVEPERISDTQYF
CSARDRGAESTDTQYF
CASSYSDFKGKQYF
CASRMTGKNTEAFF
CSAGIGGEQFF
CASSRSGAYNEQFF
CSAEDRSYNEQFF
CASSPSDWDDTQYF
CSASLQGRAAEQYF
CASSLRLSSGRFVGDTQYF
CAWSVSGAGETQYF
CASTPGQNTGELFF
CASSGRTGGTIFYEQYF
CASSPGDGSNEKLFF
CASSH_HEAFF
CASSLIDSLNSPLHF
CASSFTSGTGSGNTIYF
CASSFTGQTYNEQFF
CASSSIAGEGRNEQFF
CASSWSRTGELFF
CSAAGQLNTEAFF

CASSPLSGLGTGELFF
CASSSLSGTGAGWAKNIQYF
CASSLRGTTQETQYF
CASSVGGTEAFF
CSARVPTTSGRINEQYF
CASSLGLGGRHGGGQFF
CSALSGRGRNEQFF
CASSQGPPGIFQDSPLHF
CASTFRTGELFF
CASSFTGLNTEAFF
CSARDQAATIEETQYF
CASSASGANVLTF
CASSFRGGPHEQYF
CASSFLTSGASYEQYF
CSVETGGSGNTIYF
CSVDGTGSGNEQFF
CASSLEGNRSPLHF
CASSLVSGHNEQFF
CASSAGGDEQFF
CAVLAETQYF
CSPRGAGGPRGYEQYF
CASSYRSTDTQYF
CASSFVNMKANQPQHF
CSARGGPPYNEQFF
CSAGQSSRETQYF
CASSWDRAGYTGELFF
YAWSTRTYQGEF
CASSLAPQETQYF
CASSLYRNTGELFF
CSVEGGRAPTDTQYF
CSVLDSNSPLHF
CASSSGLSTDTQYF
CASSLLGLPLHF
CASSYVTGVDEQYF
CASSYSREAISGNTIYF
CASQQGDTEAFF
CASSYSRQGGYEQYF
CASSAVQGWSGNEQFF
CASSPAGGTTYEQYF
CASRPRVSGRRTNTGELFF
CASSDRGYEQFF
CAWSVRSTVYNSPLHF
CSAGGADYEQYF
CASSQAPKETQYF
CASSATSEPSYNEQFF

CSATPETDHDYEQYF
CASAGMSSYEQYF
CASRTNLGTEAFF
CSARRTGDHTDTQYF
CASGREYEQYF
CSARQGGTEGPEQYF
CASSLRLPGDEQFF
CAWRGRGPGNTIYF
CSAALELAATDTQYF
CSASDNYGYTF
CASSDGGARDRTQYF
CAWSAGGRETQYF
CSAPGTGRTDTQYF
CASSLTGGAETQYF
CSARDRAGRNEQYF
CAWTPGALIEAFF
CASSFRVAYGYTF
CASSYSRVFGDEQFF
CSAPRTLGGEQYF
CASSQGLRGNEQFF
CASSYGLGGDTEAFF
CASSFQLASSYNEQFF
CASSLGAMYEQYF
CASKRTGGSYEQYF
CATSLRENTEAFF
CASSFRPNPNTDTQYF
CASSLAAQGMDTEAFF
CATSRDRQPGRGTANTGELFF
CSARDMSGSVNTQYF
CASSFLPTSGRYEQYF
CSARTSNEQFF
CSASKSQGSAQHF
CATSRALAGGQETQYF
CASRSGTSGGPHEQYF
CATSRDTPAGQGGSYEQYF
CASSLLGGSETQYF
CSARDEGVRLFF
CASSSLRPGLSDEQYF
CASSQVRTEAFF
CASNKESQGYEQYF
CASGQGASTDTQYF
CASSIQGAALYGYTF
CASSYSC_GIVEAFF
CASSPGTGVYEDTQYF
CAWSVPGGNEQFF

CASSPSTSSGKINEQFF
CASSSQVYEQYF
CASSPEGPTEAFF
CSAGLAGADTQYF
CSVEDRQGNTEAFF
CSAQQPGGEAFF
CASSFPGHKYEQYF
CSALPGLGSDEQFF
CSLRGDYGTDTQYF
CSGLQGRETEAFF
CASSSGGEQFF
CAWSTRTGGNRATNEKLFF
CSVSDAYEQYF
CSAIRTGENGYTF
CSVGVGRTGYTF
CASSPGQRKDTQYF
CSAGLAGADTQYF
CASSLRGQANTGELFF
CASSFLRDAYTF
CASSLARVGVNNEQFF
CASSSQTSNSYEQYF
CASSWWGSYNEQFF
CASSLVGSSSGANVLTF
CATSRQSQGLAGTDTQYF
CASSLGSGFAQTQYF
CARKSYEQYF
CATSRGQGAADGYTF
CASSSEGGIEAFF
CASSQFQLMNTEAFF
CSARAGAGNQPQHF
CATSRGGDGADTQYF
CSASGTSTDTQYF
CSAIAAGANVLTF
CASRRTVDGANVLTF
CSAPAGGIYGYTF
CASSLEGSGELFF
CSARSTSGTHEQYF
CASSPLAGVNNEQFF
CASLRGGTGELFF
CASSLEGHATHEQFF
CASSTQWGRGFSYEQYF
CASSVFGWGPQETQYF
CASSLAEVRLSGNTIYF
CASSLGSQRGPQYF
CASSLAGGPYNEQFF

CASSYGNSLNTEAFF
CASSQVAGGGTEAFF
CATSRDREQTEQFF
CASSSPVG_SSYEQYF
CATSREASSGQYEQYF
CASSLSVGLASGITYEQYF
CSAREGGLTYEQYF
CSARRGTPSAFF
CASSPPGTDTQYF
CASSHGHHGGETQYF
CSARDQVQETQYF
CASSPTGGSGYTF
CASSRDLDSYEQYF
CSGARIAEAFF
CASRITGGYEQYF
CASSPGLAGDHEQYF
CSVESGRGVLPQYF
CSAGRTGGGEAFF
CASSETNYGYTF
CASSSPGQPYEQYF
CSAEFGTANEKLFF
CASSRTGGSYNEQFF
CSGRTSAPDTQYF
CASSVGGTANTEAFF
CSALDRPLYNEQFF
CASSLAQVGDYGYTF
CASSARGSGNTIYF
CASSPSGSYNEQFF
CASSS*_DQPQHF
CASSWTGAGKLFF
CASSPGTPGNQPQHF
CASSLLYGTSDTQYF
CASSSRGGSSATQYF
CASSLARSANEKLFF
CASREGSDYEQYF
CASSLFLSGANVLTF
CASSFRDSTDTQYF
CSATLPERDFYEQYF
CASSVRGGRTFF
CASSQEE_GKNEQFF
CASSPLRAFSAGAGEQFF
CASSYSDGNNSPLHF
CASSYSAGGSYNEQFF
CASSYSLRLAGESEQYF
CSASRGQGFYEQYF

CASAQGLENEQFF
CSARRDSNTGELFF
CSATGEKSWNSPLHF
CASSFRGRSTDTQYF
