# Supplementary material for: Circulating and Tissue-Resident CD4+ T Cells With Reactivity to Intestinal Microbiota Are Abundant in Healthy Individuals and Function Is Altered During Inflammation
Source: Gastroenterology. 2017 Nov;153(5):1320–1337.e16. doi: 10.1053/j.gastro.2017.07.047 (PMC5687320; doi:10.1053/j.gastro.2017.07.047)
Supplement: Supplementary Table 5 — Clinical Characteristics of Oxford Cohort Patients Assessed in This Study for Gene Expression Analysis (Related to Figures 5E and 7D) [file mmc5.pdf]

Supplementary Table 5. Clinical characteristics of Oxford cohort patients assessed in this study for gene expression analysis.

Characteristic	Control (*n*=13)	UC (*n*=31)	CD (*n*=27)
**Male/female**	3/9	13/17	11/16
**Median (IQR) age at sampling (years)**	58 (41–65)	48 (35–62)	37 (24–61)
**Median (IQR) age at diagnosis (years)**	n/a	31 (24–39)	25 (18–46)
**Median (IQR) disease duration (years)**	n/a	9 (3–23)	9 (6–13)
**Median (IQR) C-reactive protein (mg/l)**	n/a	6.0 (1.1–14.9)	11.1 (3.8–46.3)
**Median (IQR) peripheral blood leukocytes (10^9/l)**	8.8 (8.3–9.0)	8.3 (7.0–9.7)	8.0 (6.5–10.7)
**Current medication at sampling**			
**5-Aminosalicylates**		21	1
**Corticosteroids**		8	1
**Azathioprine/6-mercaptopurine**		7	10
**Infliximab/adalimumab**		1	9
**Unknown**		4	7

Demographic and clinical characteristics of IBD patients analysed in **Figure 5E** and **7D**
