# Supplementary material for: Circulating and Tissue-Resident CD4+ T Cells With Reactivity to Intestinal Microbiota Are Abundant in Healthy Individuals and Function Is Altered During Inflammation
Source: Gastroenterology. 2017 Nov;153(5):1320–1337.e16. doi: 10.1053/j.gastro.2017.07.047 (PMC5687320; doi:10.1053/j.gastro.2017.07.047)
Supplement: Supplementary Table 6 — Clinical Characteristics of Oxford Cohort Patients Assessed in This Study for the Analysis of Microbiota-Specific CD4 T Cells (Related to Figure 7A, B, and Supplementary Figures 6A−H) [file mmc6.pdf]

Supplementary Table 6. Clinical characteristics of Oxford cohort patients assessed in this study for the analysis of microbiota-specific CD4 T cells.

Characteristic	All patients (*n*=44)	UC (*n*=20)	CD (*n*=24)
**Male/female**	24/20	10/10	14/10
**Median (IQR) age at sampling (years)**	41 (31.5-54.75)	42 (34-55.75)	38.5 (31-52)
**Median (IQR) age at diagnosis (years)**	31 (21-47)	39 (25-55)	26.5 (19.5-37.75)
**Median (IQR) disease duration (years)**	7 (2-15)	4 (1-12)	12 (6-15.75)
**Median (IQR) C-reactive protein (mg/l)**	2.1 (0.9-4.425)	1.3 (0.85-3.56)	2.6 (0.92-4.65)
**Median (IQR) peripheral blood leukocytes (10^9/l)**	7.065 (5.4-8.11)	7.1 (5.35-9.11)	6.9 (5.33-8)
**Current medication at sampling**			
**5-Aminosalicylates**		10	2
**Corticosteroids**		4	2
**Azathioprine/6-mercaptopurine**		3	12
**Infliximab/adalimumab**		2	10
**Unknown**		3	1

Demographic and clinical characteristics of IBD patients analysed in **Figure 7A, B** and **Supplementary 6A-H**
