# Supplementary material for: Circulating and Tissue-Resident CD4+ T Cells With Reactivity to Intestinal Microbiota Are Abundant in Healthy Individuals and Function Is Altered During Inflammation
Source: Gastroenterology. 2017 Nov;153(5):1320–1337.e16. doi: 10.1053/j.gastro.2017.07.047 (PMC5687320; doi:10.1053/j.gastro.2017.07.047)
Supplement: Supplementary Table 7 — Clinical Characteristics of Oxford Cohort Patients Assessed in This Study for Cell Accumulation in the Mucosa (Related to Figure 3B, C and Figure 7C). [file mmc7.pdf]

Supplementary Table 7. Clinical characteristics of Oxford cohort patients assessed in this study for cell accumulation in the mucosa.

Characteristic	Control (*n*=13)	UC (*n*=8)	CD (*n*=9)
**Male/female**	6/7	6/2	4/5
**Median (IQR) age at sampling (years)**	32.5 (25–45.5)	29.5 (25.75–39.5)	24 (18.5–29.5)
**Median (IQR) age at diagnosis (years)**	25 (22-46)	25 (17.5–28.5)	23.5 (19.5–33.75)
**Median (IQR) disease duration (years)**	n/a	4 (1–10.5)	1 (0–6)
**Median (IQR) C-reactive protein (mg/l)**	1.75 (0.15-8.3)	0.9 (0.4–6.3)	20.7 (6.3–43.2)
**Median (IQR) peripheral blood leukocytes (10^9/l)**	7.02 (5.8–8.3)	6.73 (6.018–8.54)	6.12 (5.35–11.68)
**Current medication at sampling**			
**5-Aminosalicylates**		4	2
**Corticosteroids**		1	0
**Azathioprine/6-mercaptopurine**		1	4
**Infliximab/adalimumab**		0	1
**Unknown or no treatment**		2	4

Demographic and clinical characteristics of IBD patients analysed in **Figure 3B, C** and **Figure 7C**
