# Supplementary material for: Circulating and Tissue-Resident CD4+ T Cells With Reactivity to Intestinal Microbiota Are Abundant in Healthy Individuals and Function Is Altered During Inflammation
Source: Gastroenterology. 2017 Nov;153(5):1320–1337.e16. doi: 10.1053/j.gastro.2017.07.047 (PMC5687320; doi:10.1053/j.gastro.2017.07.047)
Supplement: Supplementary Table 3 — T-Cell Receptor Vβ Usage in Expanded Microbiota-Reactive T cells Using Multiplex Polymerase Chain Reaction Assay and Deep Sequencing (Related to Figure 3) [file mmc3.pdf]

Supplementary Table 3. T cell receptor (TCR) Vβ usage in expanded microbiota specific T cells using multiplex PCR assay and deep sequencing
(related to Figure 3)

	S. typhimurium	***E. coli***	***B. animalis***	***L. acidophilus***	***F. prausnitzii***	***C. difficile***	**SEB**	**PHA**	**Vβ**
TRBV9	1.617783985	1.462695991	1.669805399	1.107458038	0.883673765	0.521920668	0.397526502	0.700678783	['1']
TRBV20-1	10.59124767	11.1543723	9.780288763	10.24398685	8.083441982	9.251043841	0.309187279	8.430041603	['2']
TRBV28	1.0358473	1.346943071	2.209667294	3.910711196	2.013617268	0.417536534	21.37809187	0.569301511	['3']
TRBV29-1	5.52839851	5.713985057	5.599497803	6.29866759	5.606258149	7.215553236	0.618374558	4.072695424	['4']
TRBV5-1	8.216945996	5.819214985	4.959196485	3.4781104	2.491670288	6.3282881	0.530035336	4.510619663	['5.1']
TRBV5-6	0.442271881	0.904977376	0.69052103	0.19034435	0.8402144	0.339248434	0.044169611	0.131377272	['5.2']
TRBV5-5	0.453910615	0.904977376	0.69052103	0.19034435	0.8402144	0.339248434	0.044169611	0.131377272	['5.3']
TRBV4-1	1.0358473	1.062822267	0.715630885	0.103824191	1.115457048	1.957202505	0.088339223	1.401357565	['7.1']
TRBV4-2	3.852420857	3.335788698	3.992467043	2.993597508	6.156743445	5.414926931	0.706713781	4.050799212	['7.1']
TRBV4-3	3.86405959	3.388403662	4.017576899	2.993597508	6.156743445	5.401878914	0.706713781	4.13838406	['7.1', '7.2']
TRBV4-3	3.86405959	3.388403662	4.017576899	2.993597508	6.156743445	5.401878914	0.706713781	4.13838406	['7.1', '7.2']
TRBV12-3	2.420856611	2.462380301	1.820464532	2.422564458	0.86918731	0.978601253	0.132508834	1.22618787	['8']
TRBV12-4	2.420856611	2.462380301	1.820464532	2.422564458	0.86918731	0.978601253	0.132508834	1.22618787	['8']
TRBV3-1	0.186219739	0.410396717	0.615191463	0.519120955	0.376647834	0.130480167	0	0	['9']
TRBV25-1	0.034916201	0.021045985	0.037664783	0	0.014486455	0	0.088339223	0	['11']
TRBV10-3	0	0.021045985	0	0	0	0	0.088339223	0	['12']
TRBV6-1	0.477188082	0.820793434	1.858129316	1.69579512	1.839779806	0.247912317	0.618374558	0.262754543	['13.1']
TRBV6-5	3.526536313	3.893507313	4.268675455	3.928015228	4.389395915	2.518267223	1.192579505	5.517845413	['13.1']
TRBV6-6	3.479981378	3.809323372	4.18079096	3.8587991	4.244531363	2.466075157	1.192579505	5.386468141	['13.1', '13.6']
TRBV6-9	0	0	0.012554928	0	0	0.013048017	0	0	['13.1']
TRBV6-2	0.53538175	0.894454383	1.305712492	1.055545942	0.782268579	0.874217119	7.155477032	0.634990147	['13.2']
TRBV6-6	3.479981378	3.809323372	4.18079096	3.8587991	4.244531363	2.466075157	1.192579505	5.386468141	['13.1', '13.6']
TRBV27	0.151303538	0.168367884	0.640301318	0.46720886	0.970592496	0.013048017	4.06360424	0.021896212	['14']
TRBV14	1.163873371	0.936546354	0.376647834	2.145699948	0.608431117	0.365344468	0.132508834	1.248084081	['16']
TRBV19	0.034916201	0.031568978	0.062774639	0.034608064	0.173837462	0.013048017	0.574204947	0	['17']
TRBV18	0.104748603	0.242028833	0.138104206	0.346080637	0.217296827	0.130480167	0	0.065688636	['18']
TRBV30	4.67877095	4.198674103	4.105461394	6.765876449	4.679125018	6.537056367	29.99116608	6.481278739	['20']
TRBV11-2	1.175512104	1.11543723	2.071563089	3.374286209	0.796755034	0.495824635	0.265017668	0.372235603	['21.3']
TRBV2	0.698324022	1.410081027	0.263653484	0.46720886	0.217296827	0.287056367	0.044169611	0	['22']
TRBV13	0.058193669	0.073660949	0.025109856	0	0.014486455	0.052192067	0	0.021896212	['23']
TRBV1	0	0	0	0	0	0	0	0	UNK
TRBV7-1	0	0	0	0	0	0	0	0	UNK
TRBV7-2	12.13919926	11.38587814	10.80979284	5.917978889	9.054034478	16.03601253	1.899293286	16.70680972	UNK
TRBV8-1	0	0	0	0	0	0	0	0	UNK
TRBV5-2	0	0	0	0	0	0	0	0	UNK
TRBV6-4	0.034916201	0.168367884	0.025109856	0	0	0	1.01590106	0	UNK
TRBV6-3	0.53538175	0.894454383	1.305712492	1.055545942	0.782268579	0.874217119	7.155477032	0.634990147	UNK
TRBV7-3	4.213221601	3.683047459	4.143126177	4.031839419	4.621179197	2.583507307	1.899293286	3.656667397	UNK
TRBV8-2	0	0	0	0	0	0	0	0	UNK
TRBV5-3	0.244413408	0.199936862	0.050219711	0.017304032	0.014486455	0.03914405	0.309187279	0.043792424	UNK
TRBV10-1	0	0	0	0	0	0	0	0	UNK
TRBV11-1	1.047486034	1.125960223	1.644695543	3.270462018	0.753295669	0.456680585	0.220848057	0.394131815	UNK
TRBV12-1	0	0	0	0	0	0	0	0	UNK
TRBV10-2	0.081471136	0.052614964	0.037664783	0	0.173837462	0	5.344522968	0.153273484	UNK
TRBV12-2	0	0	0	0	0	0	0	0	UNK
TRBV7-4	0.046554935	0.11575292	0.025109856	0.051912096	0.02897291	0.052192067	0.750883392	0.10948106	UNK
TRBV5-4	0.675046555	0.926023361	0.502197112	1.401626579	1.434159061	1.696242171	0.176678445	0.131377272	UNK
TRBV7-5	0	0	0	0	0	0	0	0	UNK
TRBV6-7	0	0	0	0	0	0	0	0	UNK
TRBV7-6	1.745810056	2.230874461	1.694915254	1.12476207	1.941184992	3.444676409	1.369257951	3.590978761	UNK
TRBV5-8	0.675046555	0.947069347	0.527306968	1.540058834	1.463131972	1.761482255	0.176678445	0.131377272	UNK
TRBV6-8	0	0	0	0	0	0	0	0	UNK
TRBV7-8	4.166666667	3.314742713	4.369114878	2.595604776	6.634796465	3.405532359	1.766784452	4.92664769	UNK
TRBV7-7	1.641061453	2.051983584	1.556811048	1.12476207	1.839779806	3.314196242	1.369257951	3.284431793	UNK
TRBV5-7	0.40735568	0.820793434	0.665411174	0.19034435	0.724322758	0.326200418	0.044169611	0.10948106	UNK
TRBV7-9	3.514897579	4.082921183	3.892027621	4.499048278	3.215993047	3.001043841	0.35335689	3.70045982	UNK
TRBV11-3	1.198789572	1.199621172	2.084118016	3.374286209	0.796755034	0.495824635	0.265017668	0.416028027	UNK
TRBV12-5	0.162942272	0.242028833	0.11299435	0.103824191	0	0.03914405	0	0.10948106	UNK
TRBV15	2.118249534	1.125960223	0.351537979	0.692161274	0.622917572	1.200417537	0.35335689	1.707904533	UNK
TRBV16	0.046554935	0.021045985	0	0.899809656	0	0	0	0.043792424	UNK
TRBV17	0	0	0	0	0	0	0	0	UNK
TRBV21-1	0	0	0	0	0	0	0	0	UNK
TRBV22-1	0	0	0	0	0	0	0	0	UNK
TRBV23-1	0	0	0	0	0	0	0	0	UNK
TRBV24-1	0.174581006	0.147321898	0.075329567	0.242256446	0.246269738	0.11743215	3.136042403	0.021896212	UNK
TRBV26	0	0	0	0	0	0	0	0	UNK
